# Wnt signaling mediates oncogenic synergy between Akt and Dlx5 in T-cell lymphomagenesis by enhancing cholesterol synthesis

**DOI:** 10.1038/s41598-020-72822-w

**Published:** 2020-09-28

**Authors:** Yinfei Tan, Eleonora Sementino, Zemin Liu, Kathy Q. Cai, Joseph R. Testa

**Affiliations:** 1 Cancer Biology Program, Fox Chase Cancer Center, Philadelphia, PA 19111 USA; 2 Genomics Facility, Fox Chase Cancer Center, Philadelphia, PA 19111 USA; 3 Histopathology Facility, Fox Chase Cancer Center, Philadelphia, PA 19111 USA

**Keywords:** Cancer, Genetics

## Abstract

The *Dlx5* homeobox gene was first implicated as an oncogene in a T-ALL mouse model expressing myristoylated (Myr) Akt2. Furthermore, overexpression of Dlx5 was sufficient to drive T-ALL in mice by directly activating Akt and Notch signaling. These findings implied that Akt2 cooperates with Dlx5 in T-cell lymphomagenesis. To test this hypothesis, *Lck*-*Dlx5*;*Lck*-*MyrAkt2* transgenic mice were generated. MyrAkt2 synergized with Dlx5 to greatly accelerate and enhance the dissemination of T-lymphomagenesis. RNA-seq analysis performed on lymphomas from *Lck-Dlx5;Lck-MyrAkt* mice revealed upregulation of genes involved in the Wnt and cholesterol biosynthesis pathways. Combined RNA-seq and ChIP-seq analysis of lymphomas from *Lck-Dlx5;Lck-MyrAkt* mice demonstrated that β-catenin directly regulates genes involved in sterol regulatory element binding transcription factor 2 (Srebf2)-cholesterol synthesis. These lymphoma cells had high *Lef1* levels and were highly sensitive to β-catenin and Srebf2-cholesterol synthesis inhibitors. Similarly, human T-ALL cell lines with activated NOTCH and AKT and elevated *LEF1* levels were sensitive to inhibition of β-catenin and cholesterol pathways. Furthermore, *LEF1* expression positively correlated with expression of genes involved in the cholesterol synthesis pathway in primary human T-ALL specimens. Together, these data suggest that targeting β-catenin and/or cholesterol biosynthesis, together with AKT, could have therapeutic efficacy in a subset of T-ALL patients.

## Introduction

T-cell acute leukemia/lymphoma (T-ALL) is thought to be derived from the malignant transformation of immature thymic T cells^1^. Normal thymic T cells undergo development under the orchestrated and tight control of cellular pathways such as Notch and Wnt signaling. Disturbances of Notch and Wnt pathways result in T-ALL in mice and humans^2^. Human T-ALL samples frequently have activating *NOTCH1* mutations^3^. Mutant NOTCH1 has a 1- to threefold increase in HES1 reporter activity^4^. Dysregulation of homeobox genes such as HOXA and HOX11 is also a hallmark of human T-ALL^5,6^. In mice, up-regulated expression of active *Notch3* has been shown to cause T-ALL^7^. In some T-ALLs, recombination activating gene (Rag)-induced genomic instability that results in recurring T-cell receptor alpha (*Tcra*)-*Myc* translocations can underlie this process^8^. This same rearrangement has also been observed in some of our *Lck-MyrAkt2* transgenic mice, in which the Lck promoter was used to direct expression of myristoylated (Myr), constitutively active Akt2 in immature T lymphocytes^9^. Recently, ~ 85% of cases of childhood T-ALL have been shown to have upregulation of β-catenin and Wnt target genes^10^. Also, the active form of β-catenin is sufficient to induce T-ALL without the involvement of NOTCH, by stalling T-cell development at the double-positive (DP) stage^11^. A recent report demonstrated that leukemic stem cells in T-ALL require activated Wnt signaling^12^. Although β-catenin transactivates its target genes via binding to TCF and LEF, interestingly, the depletion of *Tcf7* causes T-ALL in mice through the upregulation of *Lef1*^13^. Moreover, a recent study demonstrated that the β-catenin pathway is critical to NOTCH1-initiated T-ALL by binding with NOTCH1 to the *MYC* enhancer^14^.

Activation of AKT signaling is another major driving force in T-ALL. *Pten*-null mice develop T-ALL which requires Rag-mediated V(D)J recombination^15^. β-catenin plays an essential role in Pten loss-induced T-ALL, as haploinsufficiency of the β-catenin gene, *Ctnnb1*, abolishes *Pten*-null-induced lymphomagenesis^16^. We found that expression of constitutively activated Akt2 (*MyrAkt2*) in immature T lymphocytes can induce T-ALL in mice via chromosome rearrangement-mediated upregulation of Dlx5 or Myc^17^. Human T-ALL cell lines lacking PTEN expression are highly sensitive to the PI3K pathway inhibitor LY294002^18^. One investigation uncovered alterations of *PI3K*, *AKT* and *PTEN* genes in about 48% of T-ALL patient samples^19^. Another study revealed that clinical T-ALL samples have constitutive AKT activity via posttranslational inactivation of PTEN, rather than by gene alteration^20^. The relationship between NOTCH and PTEN are intertwined. Although human T-ALL cell lines harboring *NOTCH1* and *PTEN* mutations failed to respond to NOTCH inhibitors, primary murine T-ALLs were sensitive to such inhibitors^21^. Moreover, in mice, Notch cooperates with Akt signaling, as Pten loss accelerates *Notch* mutation-induced T-ALL^21^. However, the mechanism of such cooperativity remains unknown.

We recently reported that thymocyte-specific overexpression of the homeobox gene *Dlx5* induces T-ALL in mice by directly activating *Notch1*, *Notch3* and *Irs2* transcription, which results in the upregulated Notch and Akt signaling. Moreover, the resulting tumors frequently acquired *Notch1* mutations and were sensitive to Notch inhibitors. Additionally, *Pten* was frequently inactivated in these tumors, which suggests that Notch activation and Pten loss cooperate tumorigenically in these T-ALLs^22^. To address whether the Akt pathway cooperates with the Dlx5-Notch pathway in murine T-ALL development, we crossed *Lck-MyrAkt2* mice to *Lck-Dlx5* mice. We herein report that these doubly transgenic *Lck-Dlx5;Lck-MyrAkt2* mice rapidly develop disseminated thymic lymphomas with upregulation of Wnt signaling leading to enhanced cholesterol synthesis. To our knowledge, this is the first report linking Notch and Akt crosstalk directly to β-catenin activation and cholesterol synthesis in T-cell lymphomagenesis.

## Results

### *MyrAkt2* cooperates with *Dlx5* to accelerate murine T-ALL

**Figure 1:**
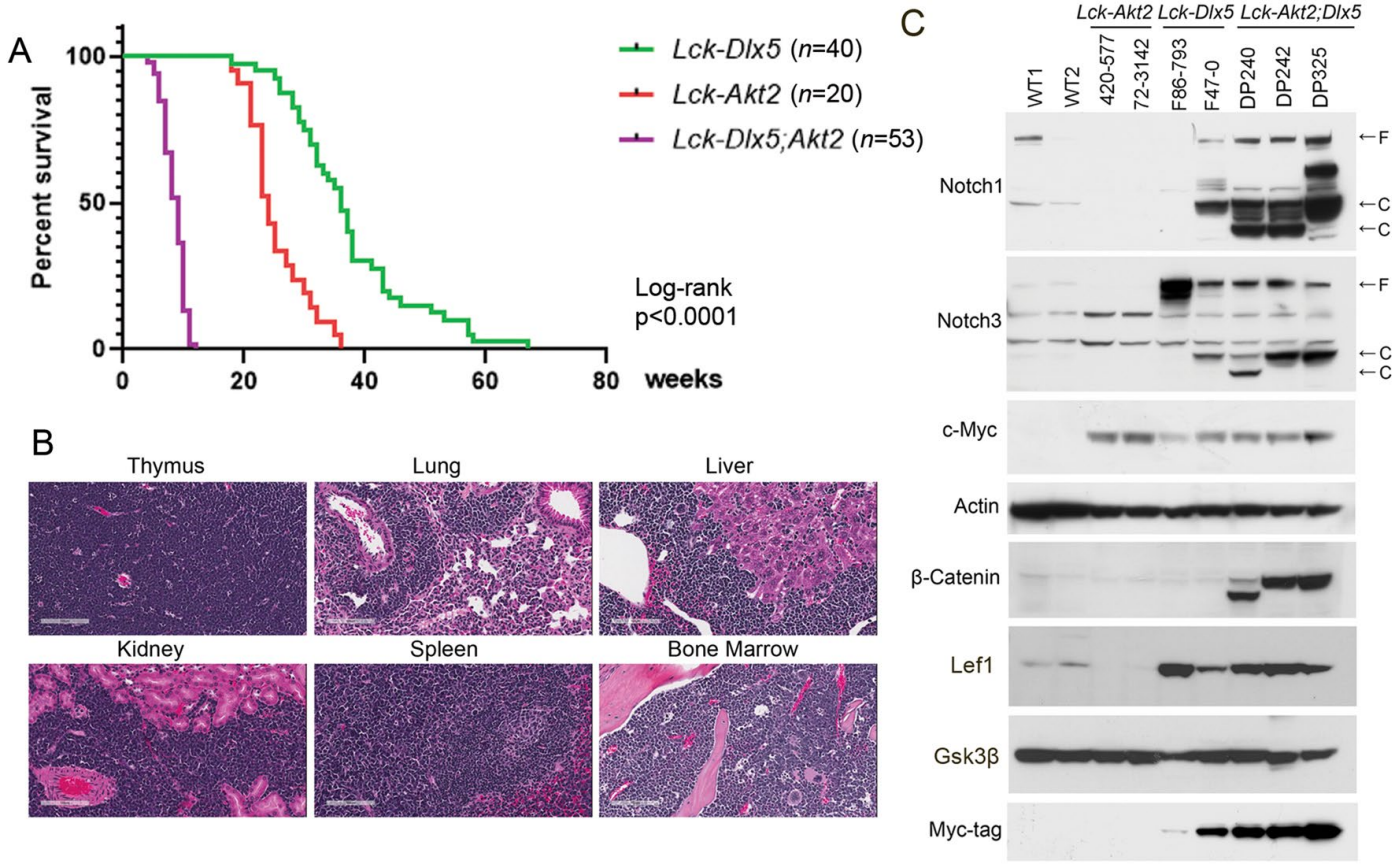
MyrAkt2 cooperates with Dlx5 to accelerate T-cell lymphomagenesis. (**A**) Survival curves of *Lck-MyrAkt2, Lck-Dlx5*, and *Lck-Dlx5;Lck-MyrAkt2* transgenic mice dying due to T-ALL. The number of animals for each genotype was as follows: *Lck-MyrAkt2*: 20 mice, *Lck-Dlx5*: 40 mice, and *Lck-Dlx5;Lck-MyrAkt2*: 53 mice. GraphPad was used to perform a log-rank (Mantel-Cox) test to compare the three survival curves, and the differences between each of the curves was highly statistically significant, *P* < 0.0001. (**B**) H&E staining depicting T-ALL infiltration and dissemination in an *Lck-Dlx5;Lck-MyrAkt2* mouse. (**C**) Immunoblot demonstrating expression of Notch1, Notch3, Myc-tagged Dlx5, Lef1, β-catenin, and c-Myc in lymphomas from *Lck-MyrAkt2*, *Lck-Dlx5*, and *Lck-Dlx5;Lck-MyrAkt2* mice compared to that of normal thymic T cells from wild-type (WT) mice.

To test whether constitutive activation of Akt cooperates with the Dlx5-Notch axis to accelerate T-ALL development, *Lck-Dlx5* transgenic mice were crossed with *Lck-MyrAkt2* mice. Tumor onset was greatly accelerated in *Lck-Dlx5;Lck-MyrAkt2* transgenic mice, with median survival being only 8 weeks versus 24 weeks in *Lck-MyrAkt2* mice and 39 weeks in *Lck-Dlx5* mice (Fig. 1A). Pathological analysis revealed that the T-cell lymphomas from *Lck-Dlx5;Lck-MyrAkt2* mice frequently involved the lung as well as liver, kidney, spleen and bone marrow (Fig. 1B; Supplementary Fig. S1A). Flow cytometric analysis revealed that the tumor cells were CD4/CD8 DP (Supplementary Fig. S1B). Karyotyping demonstrated that most tumors from *Lck-Dlx5;Lck-MyrAkt2* mice had trisomy 15 (Supplementary Table S1), the mouse chromosome that harbors the *Myc* gene. Immunoblotting uncovered upregulation of Notch1/Notch3 in tumors from *Lck-Dlx5* and *Lck-Dlx5;Lck-MyrAkt2* mice, upregulation of Myc in tumors from *Lck-Dlx5*, *Lck-MyrAkt2*, and *Lck-Dlx5;Lck-MyrAkt2* mice, and upregulation of β-catenin uniquely in lymphomas from *Lck-Dlx5;Lck-MyrAkt2* mice (Fig. 1C).

### Wnt signaling is dysregulated in lymphomas from *Lck-Dlx5;Lck-MyrAkt2* mice

**Figure 2:**
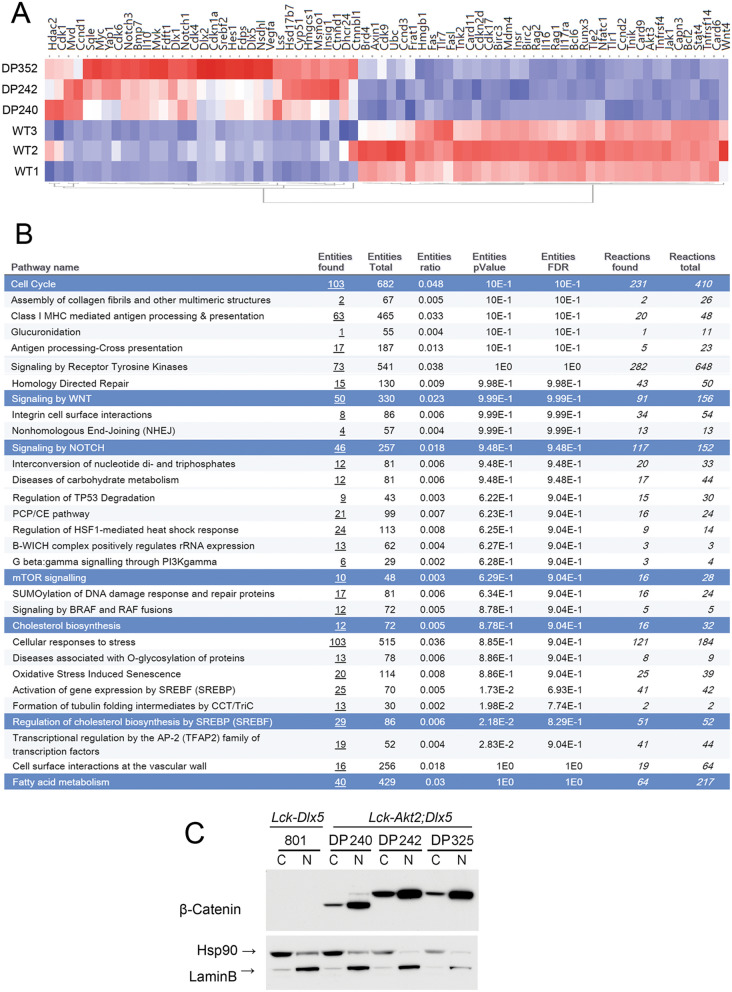
Expression profiling of lymphomas from *Lck-Dlx5;Lck-MyrAkt2* mice by RNA-seq. (**A**) Heatmap demonstrating significantly altered expression of genes in T-cell lymphoma cell lines DP240, DP242, and DP352 from *Lck-Dlx5;Lck-MyrAkt2* mice as compared to that in wild type (WT) thymic T cells. (**B**) Reactome pathway analysis showing the major altered signaling pathways in *Lck-Dlx5;Lck-MyrAkt2* lymphomas. (**C**) Cellular fractionation depicting the mainly nuclear location of β-catenin protein in *Lck-Dlx5;Lck-MyrAkt2* lymphoma cells. Hsp90 and Lamin B, which predominantly localize to the cytoplasm and nucleus, respectively, were used as controls.

To help elucidate the mechanism underlying lymphomagenesis in *Lck-Dlx5;Lck-MyrAkt2* mice, RNA-seq analysis was performed on T cell lymphoma cells from three *Lck-Dlx5;Lck-MyrAkt2* mice (DP240, DP242, DP352) and thymic T cells from three wild-type (WT) mice. The comparison revealed 1,294 up-regulated genes and 2,728 down-regulated genes in tumor cells versus normal thymic cells with > threefold expression change. A representative list of these genes is summarized in the heatmap shown in Fig. 2A. Among the up-regulated genes observed in lymphomas from *Lck-Dlx5;Lck-MyrAkt2* mice, *Notch*, *Myc*, and *Ccnd1* were previously reported to be upregulated in lymphomas from *Lck-Dlx5* mice, and Myc was previously shown to be upregulated in *Lck-MyrAkt2* mice^22^. However, upregulation of genes involved in the Wnt pathway and cholesterol synthesis were unique to the lymphomas from the *Lck-Dlx5;Lck-MyrAkt2* mice. Moreover, upregulation of *Vegf* and down-regulation of many apoptosis-related genes and *Jak-Stat* genes were seen only in tumor cells from *Lck-Dlx5;*Lck-*MyrAkt2* mice. Reactome pathway analysis identified Wnt, SREBF2 (Sterol Regulatory Element Binding Transcription Factor 2) regulation of cholesterol biosynthesis, Notch, mTOR, cell cycle, and p53 as the major altered signaling pathways in *Lck-Dlx5;Lck-MyrAkt2* lymphomas (Fig. 2B). Dlx1 and Dlx2, two other Dlx family members, were upregulated, which may amplify the role of Dlx5, given that they can bind to similar target genes. Interestingly, β-catenin mRNA was not changed, and this was validated by real-time PCR (Supplementary Fig. S2). Instead, negative regulators of β-catenin protein, including TNK2, Apc2 and Dvl2, were downregulated, which should result in activation of β-catenin. Accordingly, a cellular fractionation study demonstrated strong nuclear localization of β-catenin, suggesting a high activation level of this protein (Fig. 2C). Notably, immunoblot analysis demonstrated that DP240 cells have a weak full-length β-catenin band and a strong truncated form of the protein (Figs. 1C,2C). While N’-truncation has been shown to constitutively activate β-catenin^23^, DNA sequence analysis did not uncover a mutation in *Ctnnb1* in DP240 cells, suggesting a post-transcriptional alteration. Neither *Apc* nor *Axin*, which encode negative regulators of β-catenin, was found to be mutated.

### β-catenin signaling is essential for survival of *Lck-Dlx5;Lck-MyrAkt2* lymphoma cells

**Figure 3:**
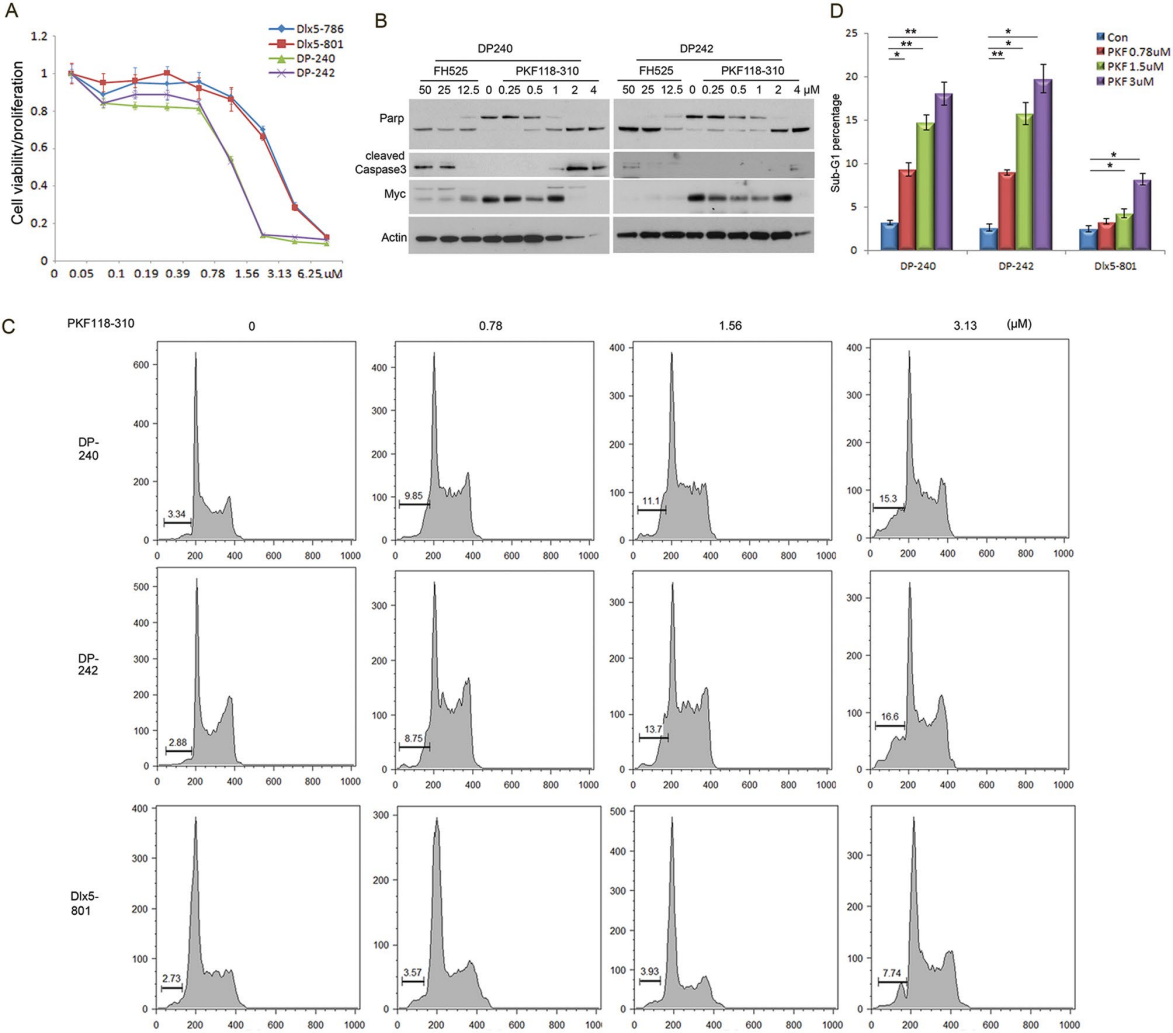
Inhibition of β-catenin signaling diminishes cell survival in *Lck-Dlx5;Lck-MyrAkt2* lymphoma cells. (**A**) MTS assay showing dose-dependent effects of β-catenin inhibitor PKF118-310 on viability of lymphoma cell lines DP240 and DP242 from *Lck-Dlx5;Lck-MyrAkt2* mice as well as on cell lines Dlx5-786 and Dlx5-801 from *Lck-Dlx5* mice. (**B**) Western blot analysis demonstrating that β-catenin inhibitor PKF118-310 promotes caspase 3 activity and PARP cleavage. Lysates for cell lines DP240 and DP242 were run on separate gels. (**C**) FACS analysis showing that PKF118-310 triggers apoptosis, as shown by sub-G1 DNA peak, in lymphoma cells DP240 and DP242 from *Lck-Dlx5;Lck-MyrAkt2* mice. Lymphoma cell line 801, from an *Lck-Dlx5* mouse, was used as a control. (**D**) Histogram depicting the sub-G1 populations in lymphoma cells from *Lck-Dlx5;Lck-MyrAkt2* mice and a control *Lck-Dlx5* mouse after treatment with PKF118-310. An Anova test was used to determine the statistical significance between placebo-treated cells and cells treated with the indicated doses of PKF, with *P *values shown with asterisks: *, *P* < 0.05; **, *P* < 0.005.

To determine if β-catenin plays a role in the survival of lymphoma cells from *Lck-Dlx5;Lck-MyrAkt2* mice, the cells were treated with PFK118-310, an inhibitor that interferes with β-catenin’s binding to Tcf/Lef. An MTS assay demonstrated that lymphoma cells from these double transgenic mice were highly sensitive to PFK118-310-induced cell death when compared to lymphoma cells from *Lck-Dlx5* mice (Fig. 3A). Immunoblot analysis of lymphoma cells from *Lck-Dlx5;Lck-MyrAkt2* mice demonstrated that PFK118-310 also promotes caspase 3 activity and consequent Parp cleavage (Fig. 3B). Moreover, flow cytometric analysis revealed a marked increase in apoptotic sub-G1 cells, suggesting that Wnt signaling plays an essential role in the survival of *Lck-Dlx5;Lck-MyrAkt2* lymphoma cells (Fig. 3C,D). Another inhibitor of β-catenin binding to Tcf/Lef, FH535, showed similar pro-apoptotic effects at higher concentrations (Supplementary Fig. S3).

### Identification of a β-catenin-controlled signaling network

**Figure 4:**
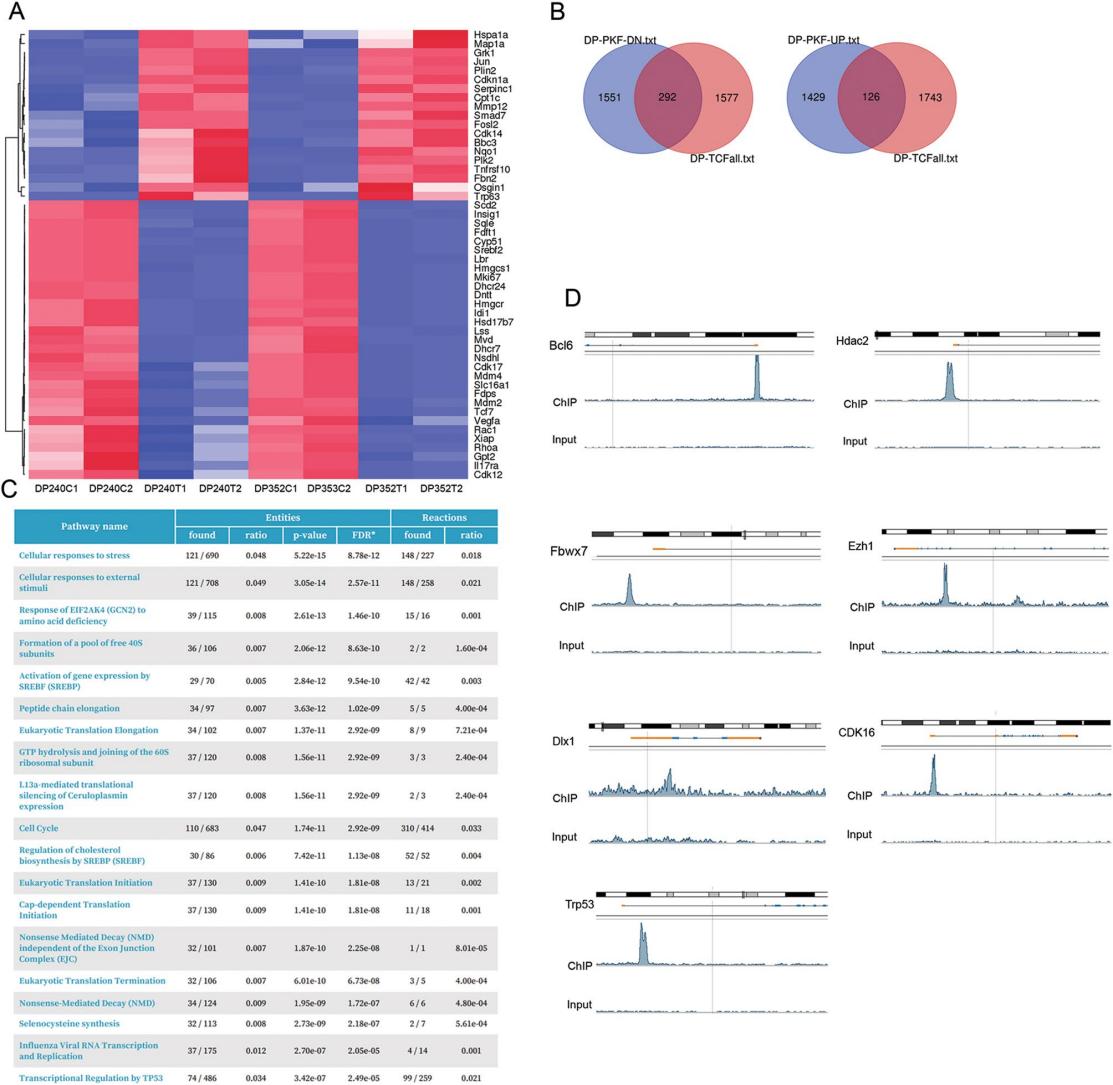
RNA-seq and ChIP-seq reveal that Wnt signaling controls a downstream network. (**A**) Heatmap of representative genes with altered expression after treatment with β-catenin inhibitor PKF118-310 in double-positive T cell lymphoma cells from *Lck-Dlx5;Lck-MyrAkt2* mice. Abbreviations: C1, C2 = placebo-treated controls; P1, P2 = PFK118-310-treated cells. (**B**) Venn diagram depicting overlapping genes identified by RNA-seq and Chip-seq, using a TCF7 antibody. (**C**) Pathway analysis indicating the main pathways directly controlled by Wnt signaling, including regulation of cholesterol biosynthesis. (**D**) Representative ChIP-seq peaks called by MACS pipeline, including *Bcl6*, *Hdac1*, *Fbwx7*, *Ezh1*, *Dlx1*,*Cdk16*, and *Trp53*.

To investigate the downstream Wnt signaling network and direct targets of β-catenin/Tcf/Lef, RNA-seq and ChIP-seq analyses were performed. Lymphoma cells from *Lck-Dlx5;Lck-MyrAkt2* mice were treated with PFK118-310 at 1 µM for 20 h and then subjected to whole transcriptome analysis, which revealed altered expression of genes encoding Srebf2-cholesterol synthesis pathway members and cell cycle regulators (Fig. 4A). Genes commonly up- or down-regulated in lymphoma cell lines from *Lck-Dlx5;Lck-MyrAkt2* mice upon β-catenin inhibition are depicted in the Venn diagram shown in Supplementary Fig. S4A. Strikingly, genes encoding nearly all major components in the cholesterol synthesis pathway were upregulated (Supplementary Fig. S4B). ChIP-seq analysis was performed to pull down β-catenin**/**Tcf7-bound DNA fragments for analysis by NGS. Upon treatment with PFK118-310, 292 downregulated genes and 126 upregulated genes had Tcf-bound DNA peaks based on the ChIP-seq analysis (Fig. 4B). *Lef1* and *Axin2* were their own targets of this pathway (Supplementary Fig. S4C). Reactome pathway analysis indicated that the main pathways directly controlled by β-catenin signaling included those belonging to Srebf-related cholesterol biosynthesis, p53 regulation, as well as Mapk and Notch signaling (Fig. 4C). Interestingly, Dlx1 was a target of β-catenin, suggesting a sophisticated, intertwined regulatory network among homeobox genes and Wnt signaling (Fig. 4D). Other key target genes involving cell survival, proliferation, cell division, transcription, translation, and metabolism included *Tp53*, *Fbwx7*, *Bcl6*, *Cdk12*, *Ddk16*, *Polr1a* and *Cenpe* and the epigenetic modulator genes *Hdac1*, *Hdac7 and Ezh1* (Fig. 4D; Supplementary Fig. S4D).

### Cholesterol synthesis pathway is downstream of β-catenin signaling

**Figure 5:**
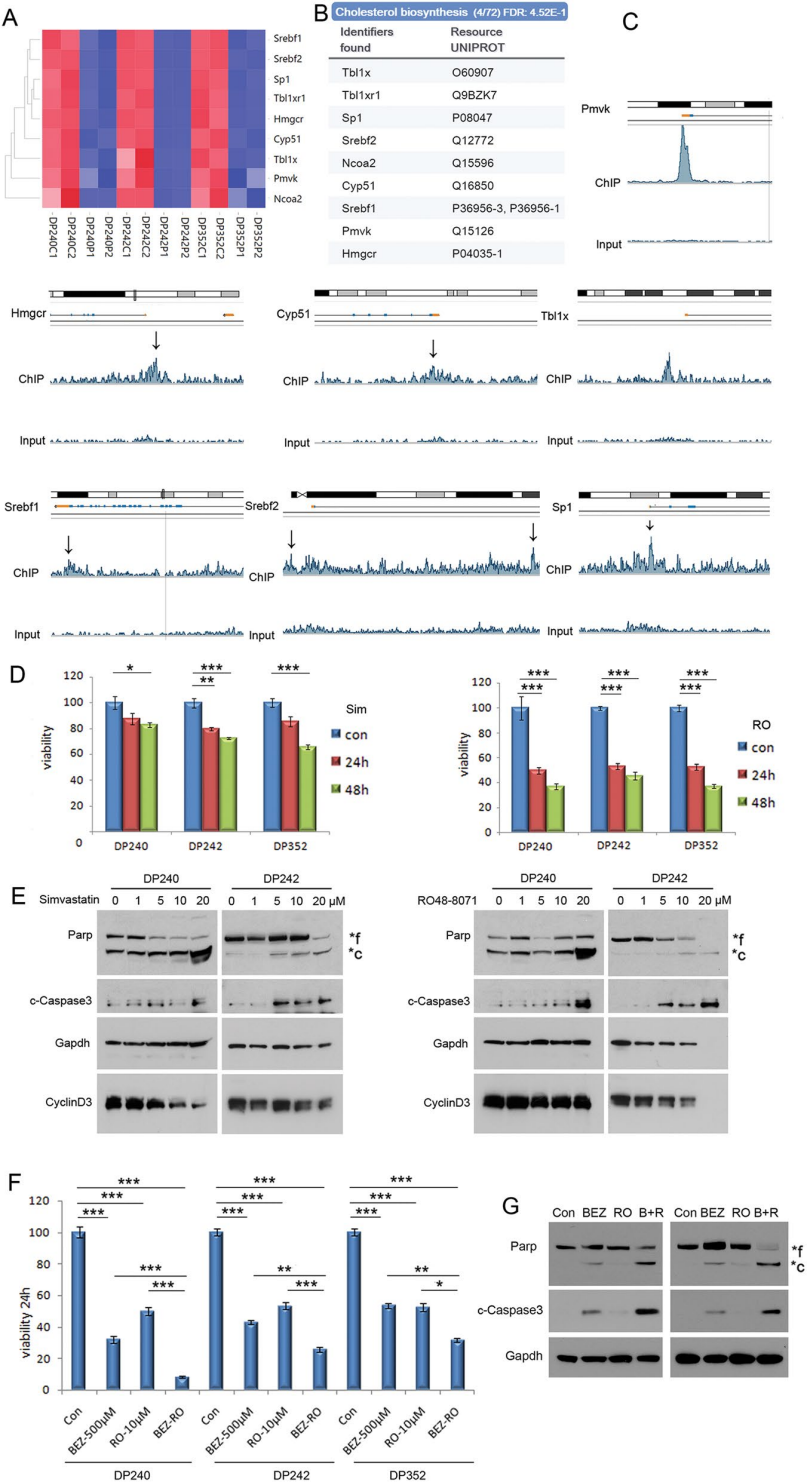
Srebf2 pathway members are downstream of β-catenin signaling. (**A**) Heatmap showing downregulated (blue rectangles) expression of cholesterol synthesis pathway members in three different T cell lymphoma cell lines from *Lck-Dlx5;Lck-MyrAkt2* mice after treatment with the Wnt inhibitor PFK118-310. Abbreviations: C1, C2 = placebo-treated controls; P1, P2 = PFK118-310-treated cells. (**B**) Reactome pathway analysis reveals upregulated members in cholesterol synthesis pathway. (**C**) ChIP-seq peaks of Tcf-binding sequences as viewed using UCSC Genome Browser. (**D**) Results of MTS assay showing reduced viability of tumor cells from *Lck-Dlx5;Lck-MyrAkt2* mice following treatment with the cholesterol synthesis inhibitor RO 48–8071 (RO) or with simvastatin (Sim). MTS assay was performed 24 h and 48 h after initiating treatment with drugs at the indicated concentrations. (**E**) Immunoblot analysis of apoptosis induced by treatment of T cell lymphoma cell lines with inhibitors of cholesterol synthesis. Parp was used as a marker of apoptosis, and Gapdh was used to depict degradation of gross cellular proteins after cell death. (**F**) MTS assays showing that the combination of RO48-8071 and BEZ235 cooperate to further diminish cell viability after treatment with drugs for 24 h. (**G**) Immunoblot demonstrating enhanced activation of caspase 3 after use of the combination of RO48-8071 and BEZ235. Note that in panels (**E**,**G**), lysates for cell lines DP240 and DP242 were run on separate gels. In panels (**D**,**F**), an Anova test was used to determine the statistical significance of differences in cell viability between control (Con) versus drug-treated cells, which are indicated by asterisks: *, *P* < 0.05; **, *P* < 0.005; and ***, *P* < 0.0005.

Further breakdown of the cholesterol synthesis pathway identified *Cyp51*, *Hmgcr*, *Ncoa2*, *Pmvk*, *Sp1*, *Srebf1*, *Srebf2*, *Tbl1x*, and *Tbl1xr1* as direct target of the β-catenin complex, each of which was downregulated in *Lck-Dlx5;Lck-MyrAkt2* cells after treatment with PFK118-310 (Fig. 5A,B). MACS peak calling identified Tcf7 binding peaks at the promoters of most of these genes (Fig. 5C). However, Tcf7 only had weak binding to the promoter and intron1 of *Srebf2* (Fig. 5C). This peak encompassed the Tcf binding consensus (T)CTTTGA(A) of the *Srebf2* promoter. Binding of the β-catenin complex to other cholesterol synthesis pathway genes was generally robust (Fig. 5C; Supplementary Fig. S5A). These findings suggest that the β-catenin complex augments cholesterol synthesis by directly transactivating these proteins or by enhancing the activity of Srebf2 on the promoters of these genes. To investigate if Srebf-mediated cholesterol and fatty acid synthesis are implicated in the survival of T cell lymphoma cells from *Lck-Dlx5;Lck-MyrAkt2* mice, cells were treated with the cholesterol biosynthesis inhibitors RO48-8071 or simvastatin. MTS assays showed reduced viability of these cells following treatment with these inhibitors (Fig. 5D), and immunoblot analysis revealed caspase 3 activation and Parp cleavage, indicative of apoptosis (Fig. 5E; Supplementary Fig. S5B). Treatment of lymphoma cells with cholesterol synthesis inhibitors in combination with Akt/mTOR inhibitors was also examined. MTS assays showed that the combination of RO48-8071 and BEZ235 and, to a lesser extent, simvastatin and RAD001, cooperated to further reduce cell viability after treatment with drugs for 24 to 72 h, with enhanced activation of caspase 3 (Fig. 5F,G; Supplementary Fig. S5C,D). Together, these findings suggest that β-catenin activation contributes to T-lymphomagenesis in *Lck-Dlx5;Lck-MyrAkt2* mice by a mechanism that includes upregulation of cholesterol synthesis via directly enhancing expression of Srebf and downstream proteins that generate a pro-survival microenvironment.

### Human T-ALL cell lines frequently show β-catenin activity and are sensitive to β-catenin inhibition

**Figure 6:**
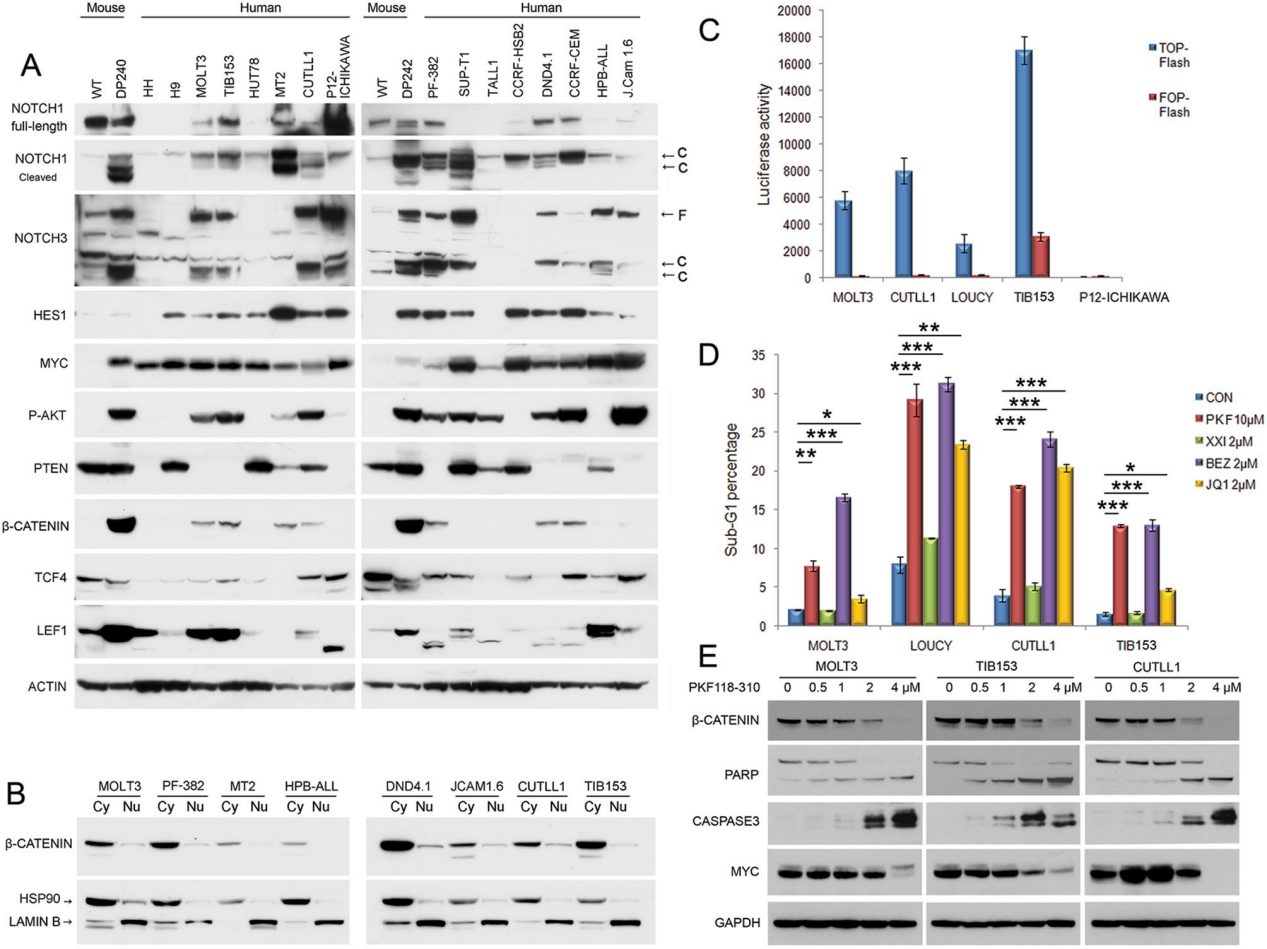
Human T-ALL cell lines frequently exhibit β-catenin activity and are sensitive to β-catenin inhibitors. (**A**) Key components of NOTCH, AKT and β-catenin signaling pathways in a group of human T-ALL lines. MYC was expressed in all human T-ALL lines. Upregulated expression of β-catenin appeared to be exclusively found in cell lines with activation of both AKT and NOTCH1. (**B**) Cellular fractionation assay demonstrating that β-catenin is partially activated in human T-ALL lines, as suggested by the small portion of β-catenin being localized in the nucleus. (**C**) Dual-luciferase assay showing that T-ALL cell lines harboring β-catenin have strong luciferase signals as compared with P12-Ichikawa cells, which lack β-catenin expression. LOUCY is shown here but not in panel A. (**D**) Cells with β-catenin activation are sensitive to β-catenin inhibitor PKF118-310, AKT pathway inhibitor BEZ235, and BRD4 inhibitor JQ1. The apoptotic sub-G1 DNA peak was measured by flow cytometry 24 h after treatment. An Anova test was used to determine the statistical significance of differences in the percentage of apoptotic sub-G1 cells between control (CON) versus drug-treated cells, as indicated by asterisks: *, *P* < 0.05; **, *P* < 0.005; and ***, *P* < 0.0005. (**E**) Western blot analysis depicting β-catenin expression after treatment of human T-ALL cell lines MOLT3, TIB153 and CUTLL1 with the β-catenin/TCF inhibitor PKF118-310 at the indicated concentration for 24 h. Immunoblot shows diminished β-catenin levels, PARP cleavage, and activation of caspase 3 in treated cells. In panel (**E**), lysates for cell lines MOLT3, TIB153, and CUTLL1 were each run on separate gels.

To test if our observation in a mouse model of acute T-cell lymphoma has translational relevance to human T-ALL, we first assessed the status of NOTCH, AKT and β-catenin signaling pathways in the human disease counterpart. Of 16 human T-ALL cell lines tested, Notch activation was observed in 14 (88%), AKT activation in 11 (69%), and β-catenin activation in 6 (38%); additionally, MYC was expressed, typically abundantly, in all 16 cell lines (Fig. 6A). Interestingly, β-catenin expression was seen only in the cell lines that had both NOTCH and AKT activity. Cellular fractionation demonstrated that a small portion of β-catenin was translocated into the nucleus (Fig. 6B). To determine if this amount of nuclear β-catenin has functional significance, a dual-luciferase assay was performed with TOP-FLASH (WT binding consensus) and FOP-FLASH (mutant binding consensus) plasmids. Cells harboring β-catenin showed strong luciferase signals compared with cells lacking its expression (Fig. 6C). Next, we treated these cells individually with β-catenin inhibitor PFK118-310, AKT pathway inhibitor BEZ235, NOTCH inhibitor XXI, or BRD4/MYC inhibitor JQ1. Cells treated with PFK118-310, BEZ235 or JQ1 each showed a marked increase in apoptotic cells, as shown by the presence of a sub-G1 peak (Fig. 6D). Treatment with PFK118-310 also resulted in reduced cell viability in a dose-dependent manner (Supplementary Fig. S6). Moreover, immunoblot analysis demonstrated that MOLT3, TIB153, and CUTLL1 cells treated with PKF118-310 for 24 h had decreased β-catenin levels paralleling caspase 3 activation and PARP cleavage (Fig. 6E). Thus, these findings indicate that β-catenin is activated in a subset of human T-ALL cell lines along with NOTCH, AKT and MYC activation, suggesting that pharmacologic inhibition of β-catenin signaling may have significance in a personalized therapeutic setting.

### The SREBF2-cholesterol pathway has a pro-survival role in human T-ALL cells

**Figure 7:**
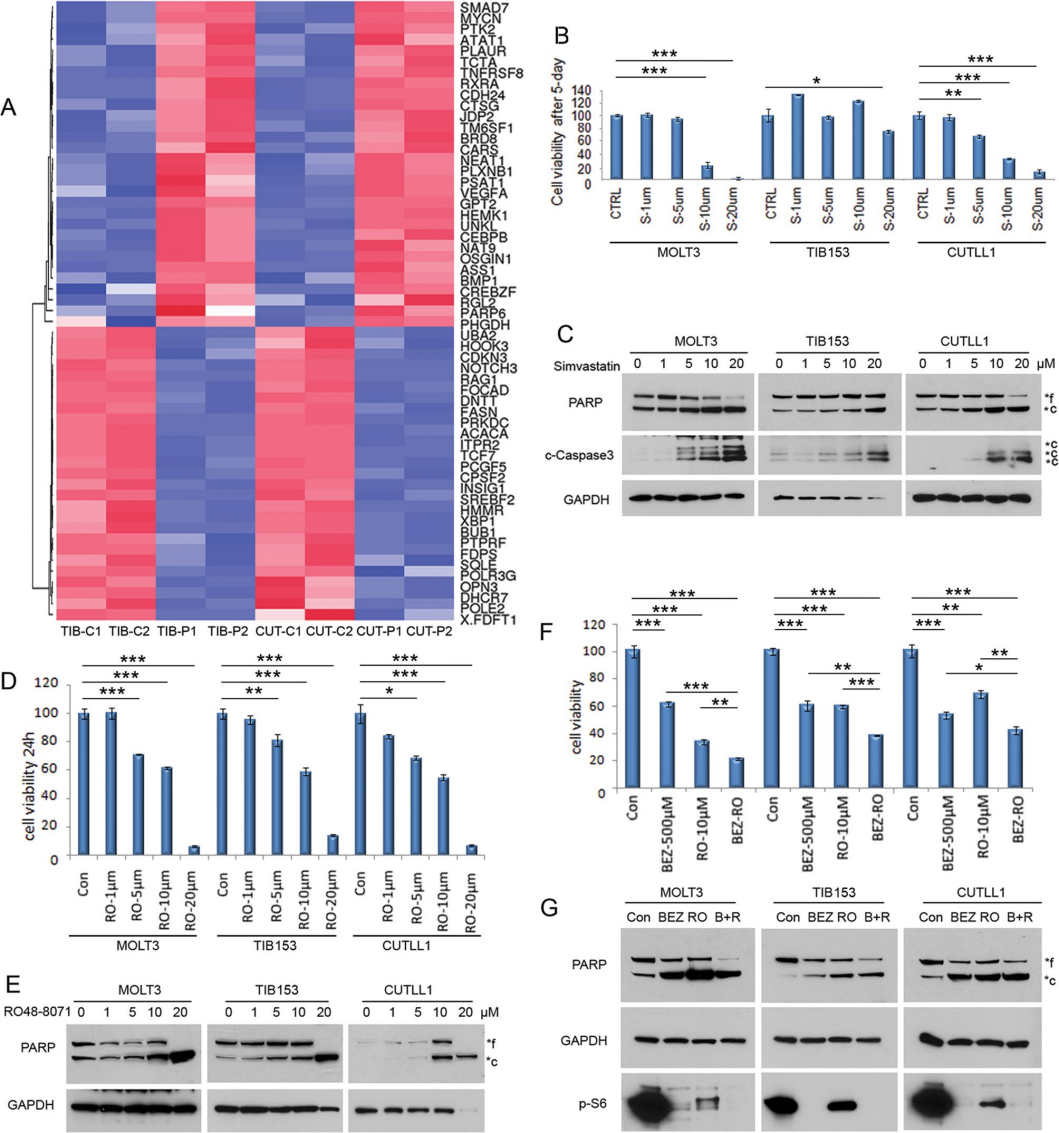
SREBF2 pathway has a pro-survival role in human T-ALL cells. (**A**) RNA-seq analysis showing that SREBF2 is a direct target of β-catenin in human T-ALL cells. Cell lines TIB153 (TIB) and CUTLL1 (CUT) were treated with 1 µM of PKF118-310 for 24 h, and RNA was subjected to whole transcriptome sequencing. Heatmap depicts genes altered by Wnt inhibition. Abbreviations: C1, C2 = placebo-treated controls; P1, P2 = cells treated with PFK118-310. (**B**,**C**) Data from MTS assays showing that increasing concentrations of the SREBF2 inhibitor simvastatin profoundly impact the viability of human T-ALL cells, with activation of caspase 3 after treatment for 5 days. (**D**,**E**) Similar effects were observed with another cholesterol synthesis inhibitor, RO 48–8071, after treatment for 24 h. (**F**,**G**) The AKT pathway inhibitor BEZ235 augments the efficacy of RO 48-8071 in inhibiting cell viability at the indicated concentrations. MOLT3, TIB153 and CUTLL1 cells were treated with inhibitors at the indicated concentration for 3 days (**B**) or 5 days (**C**). In panels (**C**,**E**,**G**), lysates for cell lines MOLT3, TIB153, and CUTLL1 were each run on separate gels. In panel (**B**), an Anova test was used to determine the statistical significance of differences in cell viability between control (CTRL) versus simvastatin-treated cells, as indicated by asterisks: *, *P* < 0.05; **, *P* < 0.005; and ***, *P* < 0.0005.

To discover genes and their associated pathways under the control of β-catenin in human T-ALL cells, WNT signaling was blocked in TIB153 and CUTLL1 cells with a β-catenin inhibitor. The cells were treated with 1 µM of PKF118-310 for 24 h, and RNA was subjected to whole transcriptome sequencing. A Venn diagram depicting commonly up- or downregulated genes in these cell lines is shown in Supplementary Fig. S7A. Reactome pathway analysis revealed a number of pathways that are significantly altered by β-catenin inhibition (Supplementary Fig. S7B). Intriguingly, SREBF2 and some of its target genes were downregulated by inhibition of β-catenin activity (Fig. 7A). Moreover, the SREBF2 inhibitor simvastatin markedly decreased the viability of some of the human T-ALL cell lines tested, as shown by MTS assay (Fig. 7B). This was accompanied by activation of caspase 3 and PARP cleavage as well as reduced AKT activity (Fig. 7C). Similar results were observed with the cholesterol synthesis inhibitor RO48-8071, which also resulted in diminished cyclin D3 levels (Fig. 7D,E). These data indicate that the cholesterol synthesis pathway has a critical role in mediating β-catenin’s oncogenic function in T-ALL. Because these cells have constitutively activated AKT activity, we were intrigued to investigate whether inhibiting AKT would intensify RO48-8071′s apoptotic effects. T-ALL cells were treated with the AKT pathway inhibitor BEZ235 together with RO48-8071 at various concentrations, and MTS assays demonstrated that BEZ235 augmented the efficacy of RO48-8071 in inhibiting cell viability (Fig. 7F). Furthermore, BEZ235 enhanced the pro-apoptotic effects of RO48-8071 as demonstrated by the activation of caspase 3 (Fig. 7G).

### The WNT pathway is intertwined with the expression of NOTCH, MYC and DLX and correlates with cholesterol synthesis in primary T-ALL specimens

**Figure 8:**
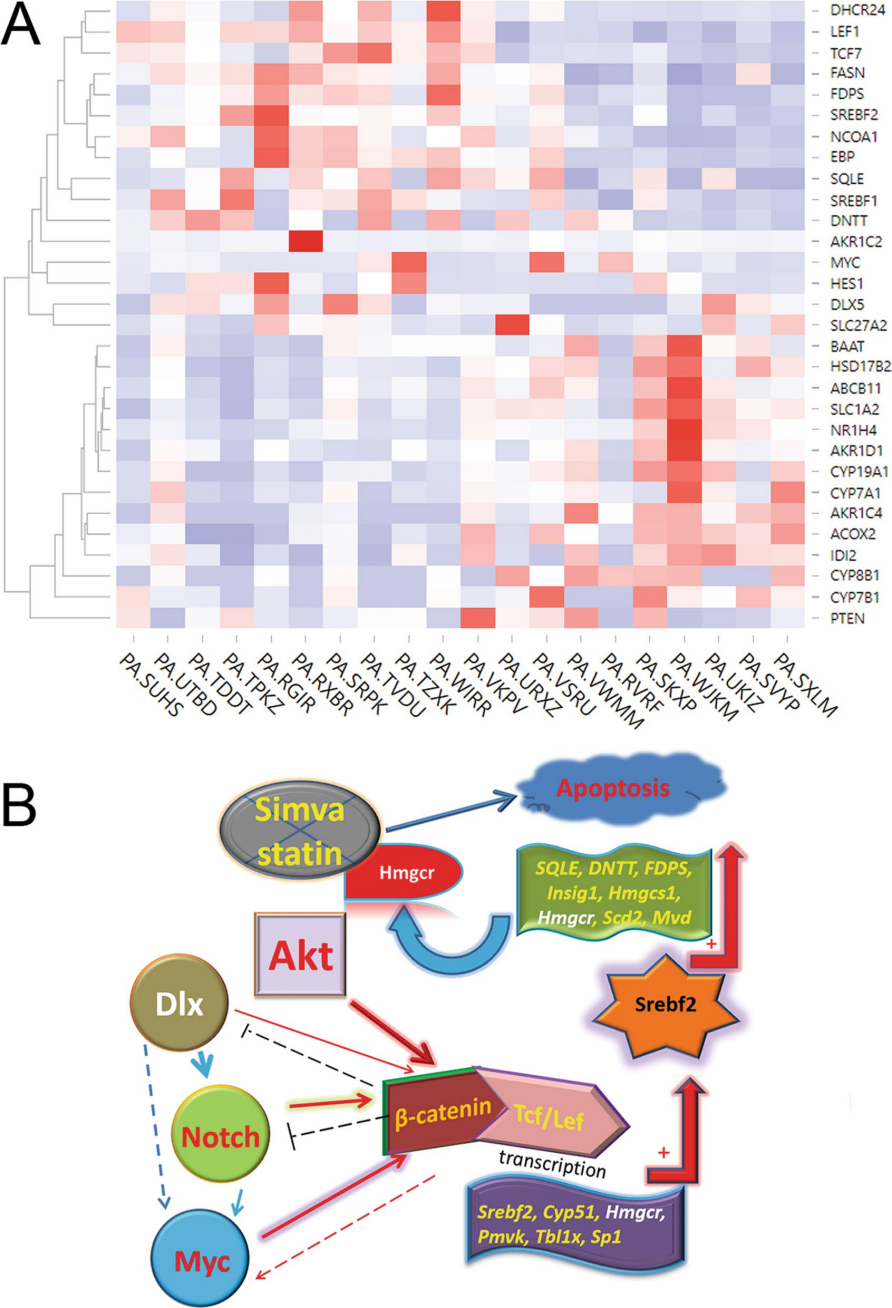
Correlation of TCF/LEF levels with SREBF2 pathway, oncogenes and tumor suppressors in human T-ALL specimens. Twenty primary T-ALL specimens were subjected to whole transcriptome analysis. (**A**) LEF1 is positively correlated with the expression of SREBF2-cholesterol synthesis pathway genes, e.g., *SREBF2*, *SQLE*, *FASN, DHCR24*, and *EBP.* (**B**) Working model of the mechanism by which β-catenin regulates cholesterol synthesis components in T-ALL. Dlx5 activates Notch, which cooperates with Akt and Myc to dysregulate Wnt signaling. The active β-catenin/TCF/LEF complex transactivates *Srebf2* and other members in the cholesterol synthesis pathway, which eventually augments the cholesterol synthesis machinery.

To further assess if our findings have translational significance, 20 primary human T-ALL specimens were subjected to whole transcriptome analysis. Importantly, *LEF1* expression was positively correlated with expression of cholesterol synthesis genes *SREBF2*, *FASN*, *SQLE*, *DHCR24* and *EBP*, but negatively correlated with expression of steroid hormone synthesis genes (Fig. 8A). Expression levels of Wnt activity markers *LEF1*, *TCF3* and *TCF7* were significantly negatively correlated with those of homeobox genes such as *HOX* and *DLX* family members as well as stem cell-related genes *GLI1/2*, *YAP1*, and *NOTCH4* (and targets *HEY1/2*, *HESX1*) (Supplementary Fig. S8A). Lower levels of Wnt activity markers are a sign of high β-catenin activity, which inhibits Wnt transcription via a feedback loop. With regard to genes implicated in cell proliferation, expression of *LEF1* and *TCF7* positively correlated with expression of *PAK2*, *MAP2K2*, *STAT5A*, *PIK3R3*, *PIK3C2B*, and *CCND3* but negatively correlated with expression of *TLR3*, *IGF1/2*, *EGFR*, *MET* and *PAK3/5* (Supplementary Fig. S8B). *LEF1* expression was also negatively correlated with that of invasion/metastatic genes such as *MMP*, *CCL*, *CXCR* and *SERPIN* family genes (Supplementary Fig. S8C). Moreover, *LEF1* expression negatively correlated with expression of stem cell maintenance genes such as *NANOG*, *POU5F1* and *SOX2*, but positively correlated with the expression of *MYC* and epigenetic modifiers *HDAC1* and *BRD3* (Supplementary Fig. S8D). Collectively, these findings suggest that Wnt activity alone is sufficient to support T-ALL survival and proliferation, such that high levels of NOTCH, Hippo, and Hedgehog signaling genes as well as homeobox and mitogen genes are not required, although this does not rule out the possibility that those pathways are involved in Wnt activation earlier in disease development. Direct or indirect negative feedback loops from Wnt signaling may have suppressed those pathways. Figure 8B represents a working model of the mechanism by which β-catenin regulates cholesterol synthesis components in T-ALL.

## Discussion

Activating mutations in *NOTCH1* occur in more than 50% of human T-ALL patients^24^. However, *Notch* mutations alone are only weakly oncogenic in the mouse, suggesting that NOTCH activation is not sufficient to trigger a T-cell malignancy^25^. In vitro studies have shown that NOTCH inhibitors mainly induce cell cycle arrest, but rarely cell death^26^. We previously implicated *Dlx5* as an oncogene when it was found to be aberrantly expressed due to a recurrent clonal chromosomal rearrangement that placed the intact *Dlx5* locus beside the *Tcrb* enhancer in a transgenic *Lck-MyrAkt2* mouse model of T-ALL^17^. Subsequently, transgenic mice specifically overexpressing Dlx5 in immature thymic T-cells (*Lck-Dlx5* mice) were shown to develop T-ALL by directly transactivating Notch and upregulating Akt signaling^22^. We thus hypothesized that constitutively active Akt2 and overexpression of Dlx5 cooperate to promote T-cell lymphomagenesis. Indeed, in the present study, we found that deaths due to T-ALL occurred 3–5 times faster in *Lck-Dlx5;Lck-MyrAkt2* mice than in either *Lck-MyrAkt2* mice or *Lck-Dlx5* mice. This marked acceleration of tumor onset and progression appears to result from the unique activation of β-catenin signaling in lymphoma cells from *Lck-Dlx5;Lck-MyrAkt2* mice, as activation of Akt, Notch, and Myc were common to the lymphomas from mice with all three genotypes. Additionally, treatment of lymphoma cells from *Lck-Dlx5;Lck-MyrAkt2* mice with β-catenin/Tcf inhibitors demonstrated that β-catenin signaling plays a critical role in promoting cell survival. RNA-seq analysis revealed that Srebf2-mediated cholesterol synthesis is tightly regulated by β-catenin signaling. Furthermore, lymphoma cells from *Lck-Dlx5;Lck-MyrAkt2* mice had elevated expression of genes encoding components of the Srebf2 pathway, and their expression was suppressed when β-catenin/Tcf inhibitors were used.

Collectively, our findings in *Lck-Dlx5;Lck-MyrAkt2* mice indicate that Wnt signaling mediates oncogenic synergy between Akt and Dlx5 in T-cell lymphomagenesis by enhancing cholesterol synthesis. Oncogenesis is sustained by cancer anabolism, of which lipid biosynthesis plays an important role. Rewired lipid metabolism is now considered a hallmark of cancer, and enhanced lipid uptake and lipogenesis are well documented in many cancers and facilitate tumorigenesis^27^. Cholesterol-reducing statins have been reported to suppress tumor cell proliferation^28^. Moreover, clinical studies have demonstrated reduced cancer-specific mortality in patients taking statins^29^. Sterol regulatory element binding protein/factors (SREBP/SREBFs) are transcription factors that control lipid homeostasis by transactivating genes encoding enzymes needed for the synthesis of cholesterol, triacylglycerol, fatty acids, and phospholipids. The SREBP-2 isoform is responsible for cholesterol synthesis, whereas the SREBP-1a isoform has been implicated in both cholesterol and fatty acid synthesis^30^. In breast cancer cells, increased levels of cholesterol and lipids, resulting from upregulation of the SREBP pathway, convey resistance to tamoxifen^31^. SREBP activity has been found to be regulated by various oncogenic or tumor suppressive mechanisms, with tumor suppressor genes usually inhibiting SREBP function, and oncogenes typically promoting and depending on SREBP activity^32^. For example, Lats2-p53 can inhibit SREBP transcription and activity^32^. Additionally, a recent study revealed that SREBP target genes can be controlled by pyruvate dehydrogenase A1^33^. However, a potential link between Wnt/β-catenin and cholesterol synthesis is now beginning to emerge. Mutations in *DHCR7*, the gene encoding delta-7-sterol reductase, impair the reduction of 7-dehydrocholesterol (7-DHC) to cholesterol and thereby directly downregulate Wnt/β-catenin signaling^34^. The combinatory RNA-seq and ChIP-seq data presented here have revealed that some key players in the Srebf2-cholesterol pathway are direct targets of Wnt signaling. A working mechanism of such action is depicted in Fig. 8B. Our data on primary human T-ALL specimens also support our finding that Wnt activity positively correlates with expression of SREBF2 and other components of the cholesterol synthesis pathway.

To our knowledge, this investigation is the first to demonstrate that the SREBF2-cholesterol biosynthesis pathway is transcriptionally regulated by WNT/β-catenin, and that the SREBF2-cholesterol pathway is pivotal to the survival of murine *Lck-Dlx5;Lck-MyrAkt2* lymphoma cells as well as a subset of human T-ALL cells. Together, these findings suggest that targeting β-catenin and/or cholesterol biosynthesis, together with AKT, could have therapeutic efficacy in some T-ALL patients.

## Materials and methods

### Reagents

Antibodies against Akt (cat. #9272), Caspase 3 (cat. #9661), Cyclin D3 (cat. #2936), HSP90 (cat. #4874), Myc (cat. #5605), Myc-Tag (cat. #2276), Notch1 (cat. #3608), PARP (cat. #9542), phospho (p)-Akt(Ser473) (cat. #4060), p-S6 ribosomal protein (cat. #2211), and Pten (cat. #9559) were from Cell Signaling (Danvers, MA). Antibodies against β-Actin (cat. #sc-47778), β-Catenin (cat. #sc-7963), Gapdh (cat. #sc-32233), Hes1 (cat. #sc-13844), Lamin B (cat. #sc-6216), LEF-1 (cat. #sc-374412), Notch3 (cat. #sc-5593), TCF-4 (cat. #sc-166699), as well as mouse anti-goat IgG-horseradish peroxidase (HRP) secondary antibody (cat. # sc-2354) were all from Santa Cruz Biotechnology (Santa Cruz, CA). Anti-rabbit IgG, peroxidase-linked species-specific whole antibody (from donkey) secondary HRP-linked secondary antibody (cat. #NA 9341) and anti-mouse IgG, peroxidase-linked species-specific whole antibody (from sheep) secondary antibody (cat. #NA 3911) were from GE Healthcare Life Sciences (Marlborough, MA). Immobilon ECL Ultra Western HRP substrate (cat. #WBULS0100), β-Catenin/Tcf inhibitor II, PKF118-310 (cat. #219331), OSC inhibitor, RO48-8071 (cat. #499635), and InSolution Simvastatin, Sodium Salt (cat. #567022) were from Millipore Sigma (Burlington, MA). JQ1 inhibitor (cat. #S7110), BEZ235 (cat. #S1009), RAD001 (cat. #S1120), FH535 (cat. #S7484), and γ-secretase inhibitor XI (cat. #S0058) were from Selleckchem (Houston, TX). Lipofectamine 2000 (cat. #11668019) was from Thermo Fisher (Waltham, MA).

### Mouse models

The transgenic mouse models *Lck-MyrAkt2* and *Lck-Dlx5-Myc-tag* were previously reported^17,22^. Mice were sacrificed upon evidence of labored breathing, hunched posture, distended abdomen, or upon a 10% change in body weight, under Fox Chase Cancer Center Institutional Animal Care and Use Committee protocol #96-36. This protocol was updated, reviewed and approved annually by the Fox Chase Cancer Center Institutional Animal Care and Use Committee, allowing for the generation, maintenance, and ethical care and monitoring of *Lck-MyrAkt2*, *Lck-Dlx5-Myc-tag,* and *Lck-Dlx5;Lck-MyrAkt2* transgenic mice. All experiments were performed in accordance with these institutional as well as NIH guidelines and regulations. A portion of each tumor was collected for protein or RNA analysis, and the remainder was placed into culture to establish tumor cell lines.

### Cell culture

Mouse T cell lymphoma cells were collected from thymic tumors and filtered through a 100-gauge mesh, as previously described^17^. Cells were maintained in Iscove’s MEM complete medium, supplemented with 15% FBS, 100 μg/mL penicillin/streptomycin, 2 mM L-glutamine, 1 mM MEM sodium pyruvate, and non-essential amino acids (Life Technologies, Grand Island, NY), and β-mercaptoethanol (Thermo Fisher). Human malignant T cell lines were a gift of Owen O’Connor (Columbia University, NY). Cells were maintained in RPMI1640 medium supplemented with 10% FBS containing 100 μg/mL penicillin/streptomycin and 2 mM L-glutamine. HEK293T cells were from ATCC and maintained in DMEM with the same supplements.

### T-ALL specimens

Primary T-ALL samples were provided by the Children's Oncology Group under NCTN Operations Center Grant U10CA180886 and NCTN SDC Grant 180899. Total RNAs were extracted using TRIzol and purified via spin column (Qiagen, Germantown, MD).

### Cell viability assay

Cell viability was measured by MTS using a CellTiter 96 AQ_ueous_ One Solution Cell Proliferation Assay (Promega, Madison WI; cat. #G3580), as previously described^17^. In brief, cells were seeded at 2 × 10^4^ cells/well in 96-well plates overnight and treated with various inhibitors for 24 or 48 h. MTS reagent (20 μL) was added to each well, followed by incubation for 1–4 h for color development. OD value at 490 nm was measured using a 96-well microplate reader (BioRad, Hercules, CA).

### Flow cytometry analysis

Flow cytometry was performed with a FACS (BD Biosciences, San Jose, CA). Cells were fixed with 70% ice-cold ethanol for 5 min, washed with cold PBS, and stained with propidium iodide for 15 min at room temperature. Data were analyzed with FlowJo software.

### Dual-luciferase reporter assay

β-catenin luciferase reporter plasmids were obtained from Addgene (cat. #12457 and #12456). 3 µg of TOP-FLASH or FOP-FLASH plasmids were transfected onto 1 × 10^6^ human T-ALL cells cultured in single wells of a 6-well plate using Lipofectamine LTX (Thermo Fisher; cat. #15,338,100). Luciferase activity was measured 24 h after transfection using a Dual-Luciferase reporter assay kit (Promega; cat. #E1910) on an Enspire alpha plate reader (Perkin Elmer, Waltham, MA).

### Western blot analysis

Immunoblotting was performed as previously described^17^. In brief, cells were lysed with 1 × cell lysis buffer (Cell Signaling; cat. #9803) supplemented with 2 mM PMSF. Bradford reagent was used to measure protein concentrations. Cell lysates were loaded into Bis–Tris gels (Invitrogen, Carlsbad, CA) and transferred onto PVDF membranes (Millipore, Burlington, MA). Membranes were blocked with 5% non-fat milk in TBST for 1 h and then incubated with primary antibodies at 4 °C overnight. After washing 3 times with TBST, membranes were incubated with secondary antibody at room temperature for 1 h and further washed three times before film exposure with ECL (Millipore).

### Real-time PCR analysis

RNA was extracted using an RNeasy column (Qiagen; cat. #74104). RNA quality was checked by running on a BioAnalyzer using RNA nano CHIP (Agilent; cat. #5067-1511). Next, 1000 ng of total RNA with RIN > 8 was used to make cDNA via reverse transcription. cDNA was generated using SuperScript II from Thermo Fisher (cat. #18064014) with random primers at 42 °C for 1 h. qPCR was performed on a QuantStudio 12K Flex Real-Time PCR System (Thermo Fisher), using PowerUp SYBR Green Master Mix (Thermo Fisher; cat. #A25742) and the default program. The *Pp1a* gene was used as the normalizing control.

#### RNA-seq and ChIP-seq

Total RNA was extracted using TRIzol and purified using an RNA miniprep kit (ZymoResearch, Irvine, CA). Construction of sequencing libraries was with a TruSeq stranded mRNA library kit (Illumina, San Diego, CA). Next generation sequencing (NGS) was performed on an Illumina HiSeq 2500 system. For each sample, approximately 30 million reads were obtained. FASTQ files were aligned to the mouse reference genome using TopHat algorithm. Differentially expressed genes were analyzed using EdgerR algorithm. The related signaling pathways were viewed on the ConsensusPath database, with analyses performed using Chipster^35^. ChIP was carried out using an Active Motif (Carlsbad, CA) kit. For input DNA, genomic DNA was sonicated to 150–800 bp using a Covaris S2 ultrasonicator (Matthews, NC). ChIP-grade TCF7 antibody was used to pull down bound DNA fragments. The Illumina library was constructed using a NEBNext Ultra II kit (Ipswich, MA), and ~ 50 million reads/sample were obtained. FASTQ files were aligned to mm10, and peaks were called using the MACS2 algorithm in Chipster. FASTQ files were uploaded to GEO.

## Supplementary information

Supplementary information 1.

Supplementary information 2.

## References

[1] Thiel E (1985). Cell surface markers in leukemia: biological and clinical correlations. Crit. Rev. Oncol. Hematol..

[2] Weerkamp F, van Dongen JJ, Staal FJ (2006). Notch and Wnt signaling in T-lymphocyte development and acute lymphoblastic leukemia. Leukemia.

[3] Weng AP (2004). Activating mutations of *NOTCH1* in human T cell acute lymphoblastic leukemia. Science.

[4] Larson Gedman A (2009). The impact of *NOTCH1*, *FBW7* and *PTEN* mutations on prognosis and downstream signaling in pediatric T-cell acute lymphoblastic leukemia: a report from the Children's Oncology Group. Leukemia.

[5] Hatano M, Roberts CW, Minden M, Crist WM, Korsmeyer SJ (1991). Deregulation of a homeobox gene, *HOX11*, by the t(10;14) in T cell leukemia. Science.

[6] Soulier J (2005). *HOXA* genes are included in genetic and biologic networks defining human acute T-cell leukemia (T-ALL). Blood.

[7] Bellavia D (2000). Constitutive activation of NF- κB and T-cell leukemia/lymphoma in Notch3 transgenic mice. EMBO J..

[8] Dose M (2014). β-Catenin induces T-cell transformation by promoting genomic instability. Proc. Natl. Acad. Sci. U.S.A..

[9] Timakhov RA (2009). Recurrent chromosomal rearrangements implicate oncogenes contributing to T-cell lymphomagenesis in Lck-MyrAkt2 transgenic mice. Genes Chromosomes Cancer.

[10] Ng OH (2014). Deregulated WNT signaling in childhood T-cell acute lymphoblastic leukemia. Blood Cancer J..

[11] Guo Z (2007). β-catenin stabilization stalls the transition from double-positive to single-positive stage and predisposes thymocytes to malignant transformation. Blood.

[12] Giambra V (2015). Leukemia stem cells in T-ALL require active Hif1α and Wnt signaling. Blood.

[13] Yu S (2012). The TCF-1 and LEF-1 transcription factors have cooperative and opposing roles in T cell development and malignancy. Immunity.

[14] Gekas C (2016). β-Catenin is required for T-cell leukemia initiation and MYC transcription downstream of Notch1. Leukemia.

[15] Guo W (2011). Suppression of leukemia development caused by PTEN loss. Proc. Natl. Acad. Sci. U.S.A..

[16] Guo W (2008). Multi-genetic events collaboratively contribute to *Pten*-null leukaemia stem-cell formation. Nature.

[17] Tan Y (2008). A novel recurrent chromosomal inversion implicates the homeobox gene *Dlx5* in T-cell lymphomas from Lck-Akt2 transgenic mice. Cancer Res..

[18] Uddin S, Hussain A, Al-Hussein K, Platanias LC, Bhatia KG (2004). Inhibition of phosphatidylinositol 3'-kinase induces preferentially killing of PTEN-null T leukemias through AKT pathway. Biochem. Biophys. Res. Commun..

[19] Gutierrez A (2009). High frequency of *PTEN*, *PI3K*, and *AKT* abnormalities in T-cell acute lymphoblastic leukemia. Blood.

[20] Silva A (2008). PTEN posttranslational inactivation and hyperactivation of the PI3K/Akt pathway sustain primary T cell leukemia viability. J. Clin. Invest..

[21] Medyouf H (2010). Acute T-cell leukemias remain dependent on Notch signaling despite PTEN and INK4A/ARF loss. Blood.

[22] Tan Y (2017). The homeoprotein Dlx5 drives murine T-cell lymphomagenesis by directly transactivating Notch and upregulating Akt signaling. Oncotarget.

[23] Miller JR, Hocking AM, Brown JD, Moon RT (1999). Mechanism and function of signal transduction by the Wnt/beta-catenin and Wnt/Ca2+ pathways. Oncogene.

[24] Grabher C, von Boehmer H, Look AT (2006). Notch 1 activation in the molecular pathogenesis of T-cell acute lymphoblastic leukaemia. Nat. Rev. Cancer.

[25] Chiang MY (2008). Leukemia-associated *NOTCH1* alleles are weak tumor initiators but accelerate K-ras-initiated leukemia. J. Clin. Invest..

[26] Chan SM, Weng AP, Tibshirani R, Aster JC, Utz PJ (2007). Notch signals positively regulate activity of the mTOR pathway in T-cell acute lymphoblastic leukemia. Blood.

[27] Cheng X, Li J, Guo D (2018). SCAP/SREBPs are central players in lipid metabolism and novel metabolic targets in cancer therapy. Curr. Top. Med. Chem..

[28] Raghu VK (2018). Biomarker identification for statin sensitivity of cancer cell lines. Biochem. Biophys. Res. Commun..

[29] Nielsen SF, Nordestgaard BG, Bojesen SE (2012). Statin use and reduced cancer-related mortality. New. Engl. J. Med..

[30] Horton JD, Goldstein JL, Brown MS (2002). SREBPs: Activators of the complete program of cholesterol and fatty acid synthesis in the liver. J. Clin. Invest..

[31] Hultsch S (2018). Association of tamoxifen resistance and lipid reprogramming in breast cancer. BMC Cancer.

[32] Aylon Y, Oren M (2016). The Hippo pathway, p53 and cholesterol. Cell Cycle.

[33] Chen J (2018). Compartmentalized activities of the pyruvate dehydrogenase complex sustain lipogenesis in prostate cancer. Nat. Genet..

[34] Francis KR (2016). Modeling Smith-Lemli-Opitz syndrome with induced pluripotent stem cells reveals a causal role for Wnt/beta-catenin defects in neuronal cholesterol synthesis phenotypes. Nat. Med..

[35] Kallio MA (2011). Chipster: user-friendly analysis software for microarray and other high-throughput data. BMC Genomics.

