# Supplementary material for: Wnt signaling mediates oncogenic synergy between Akt and Dlx5 in T-cell lymphomagenesis by enhancing cholesterol synthesis
Source: Sci Rep. 2020 Sep 28;10:15837. doi: 10.1038/s41598-020-72822-w (PMC7522078; doi:10.1038/s41598-020-72822-w)
Supplement: Supplementary file 1 — Supplementary information 1. [file 41598_2020_72822_MOESM1_ESM.pptx]

## Slide 1
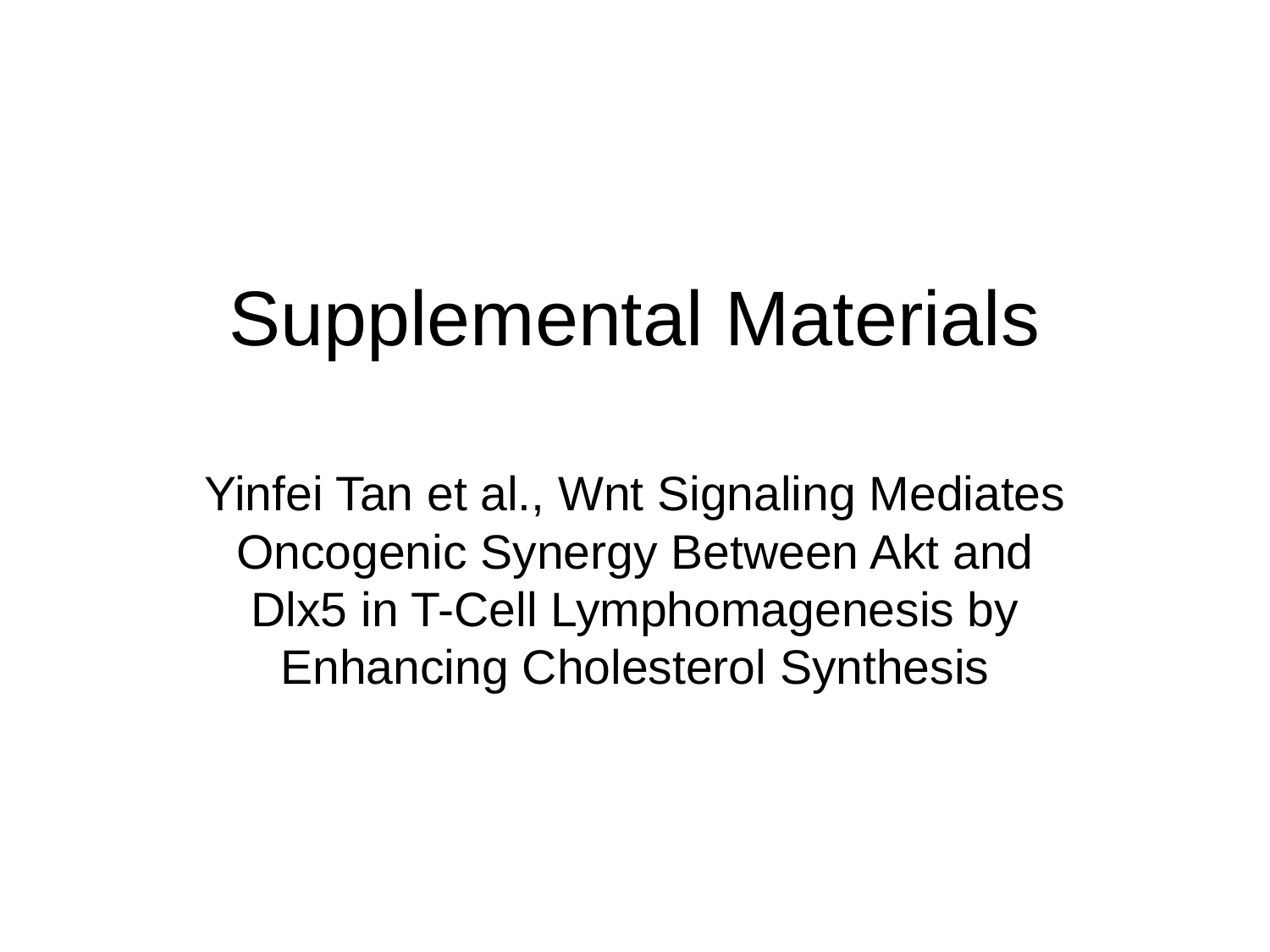

# Supplemental Materials
Yinfei Tan et al., Wnt Signaling Mediates Oncogenic Synergy Between Akt and Dlx5 in T-Cell Lymphomagenesis by Enhancing Cholesterol Synthesis

## Slide 2
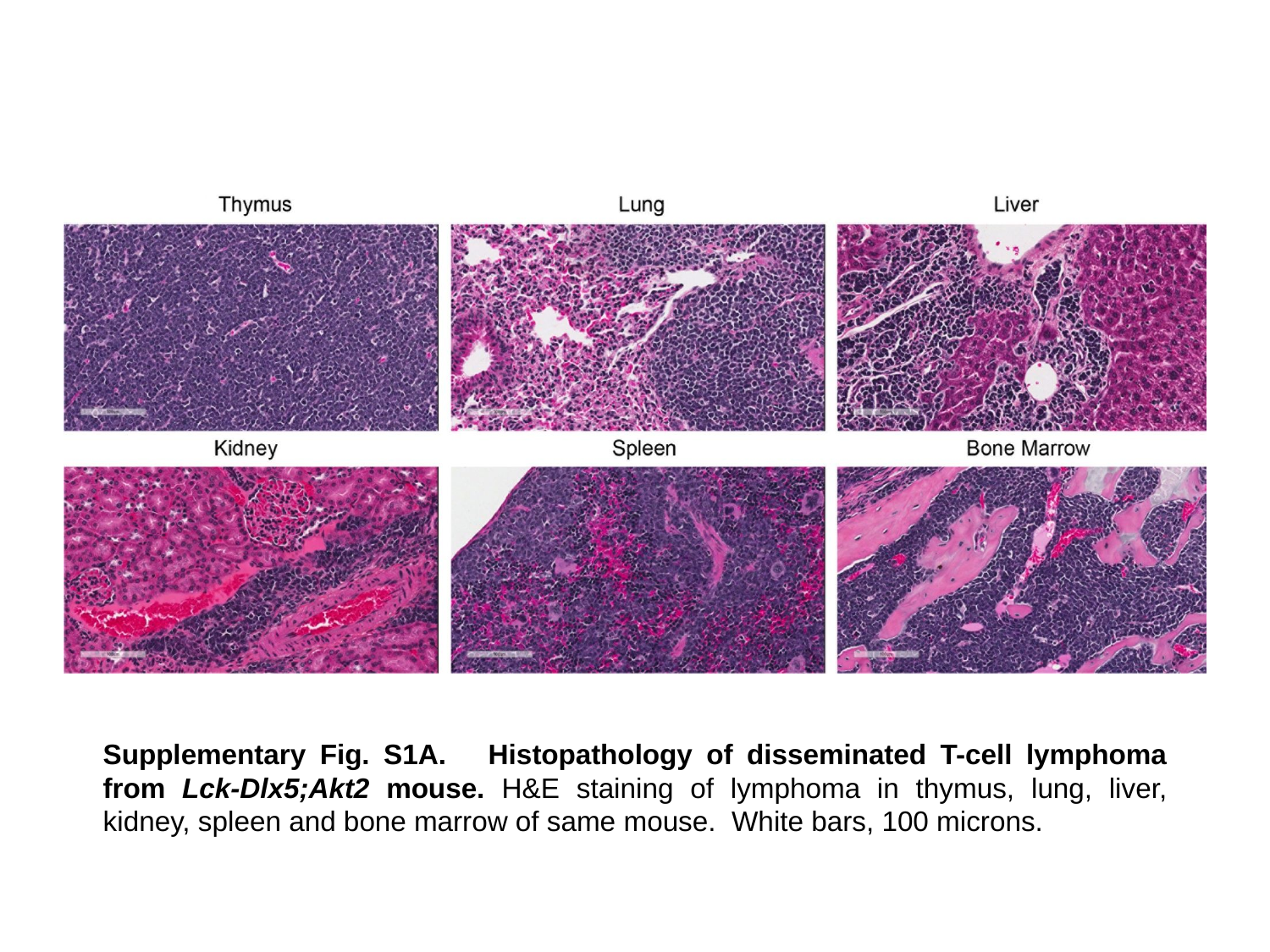

Supplementary Fig. S1A. Histopathology of disseminated T-cell lymphoma from Lck-Dlx5;Akt2 mouse. H&E staining of lymphoma in thymus, lung, liver, kidney, spleen and bone marrow of same mouse. White bars, 100 microns.

## Slide 3
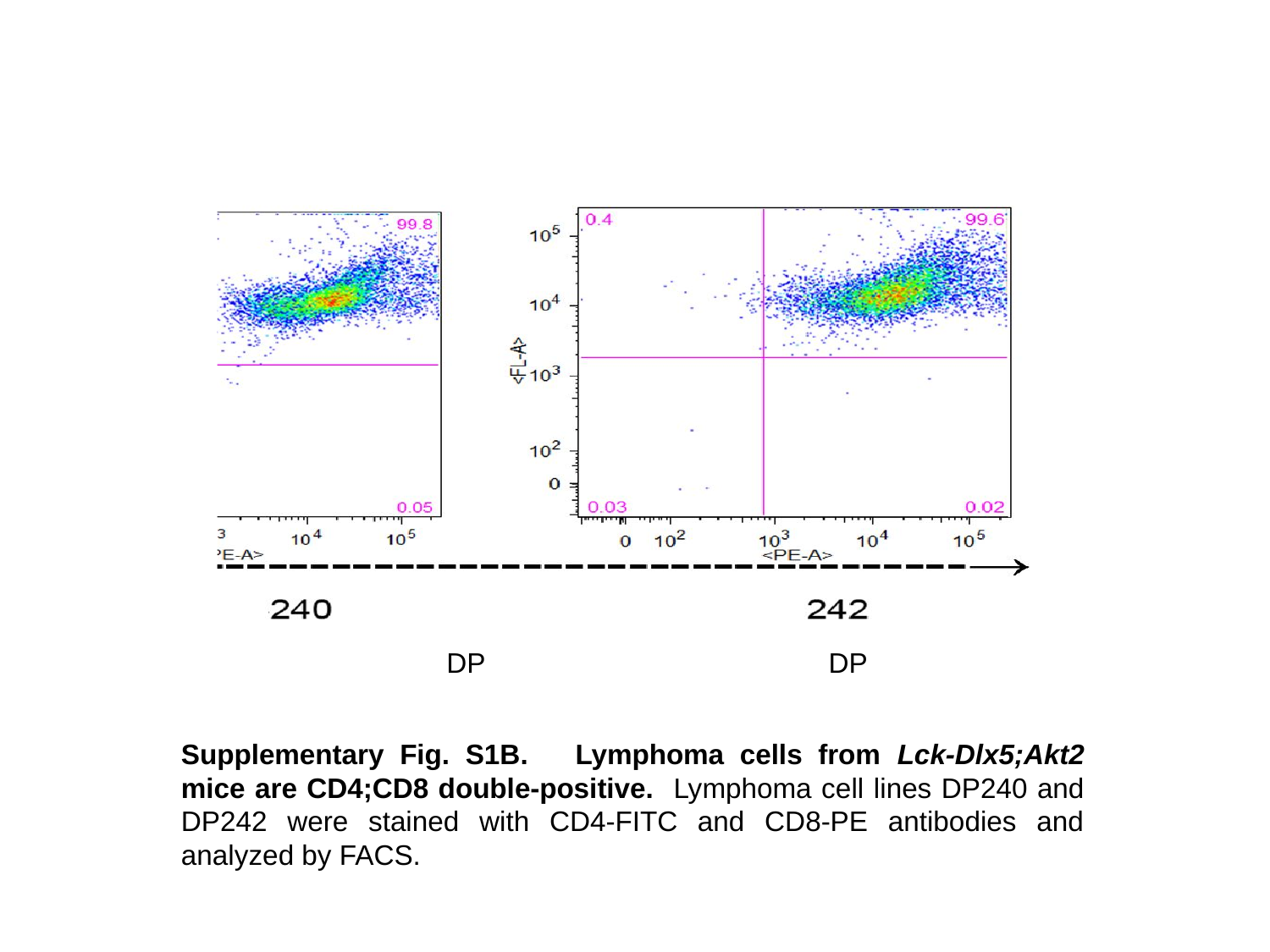

DP DP
Supplementary Fig. S1B. Lymphoma cells from Lck-Dlx5;Akt2 mice are CD4;CD8 double-positive. Lymphoma cell lines DP240 and DP242 were stained with CD4-FITC and CD8-PE antibodies and analyzed by FACS.

## Slide 4
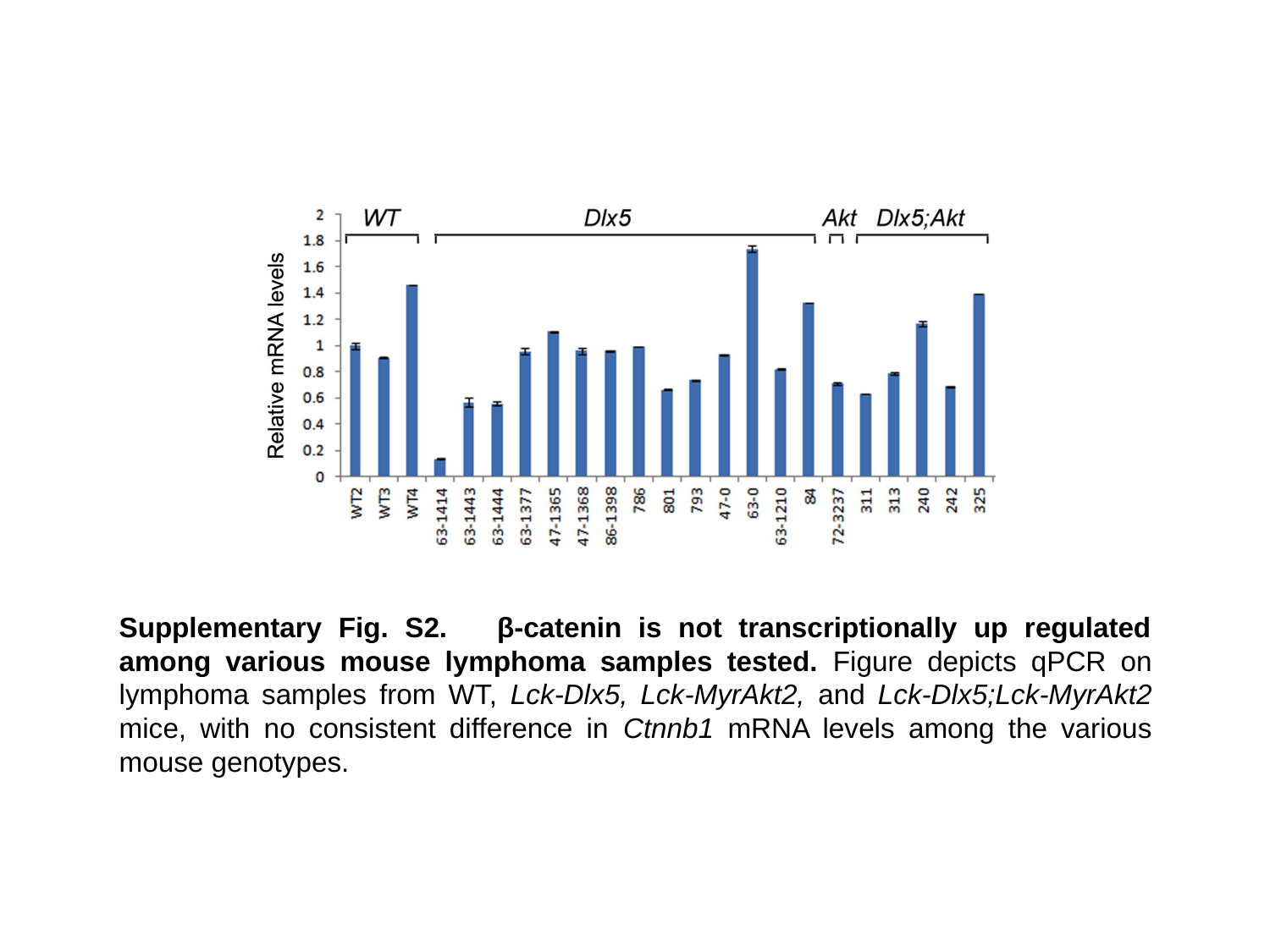

Supplementary Fig. S2. β-catenin is not transcriptionally up regulated among various mouse lymphoma samples tested. Figure depicts qPCR on lymphoma samples from WT, Lck-Dlx5, Lck-MyrAkt2, and Lck-Dlx5;Lck-MyrAkt2 mice, with no consistent difference in Ctnnb1 mRNA levels among the various mouse genotypes.

## Slide 5
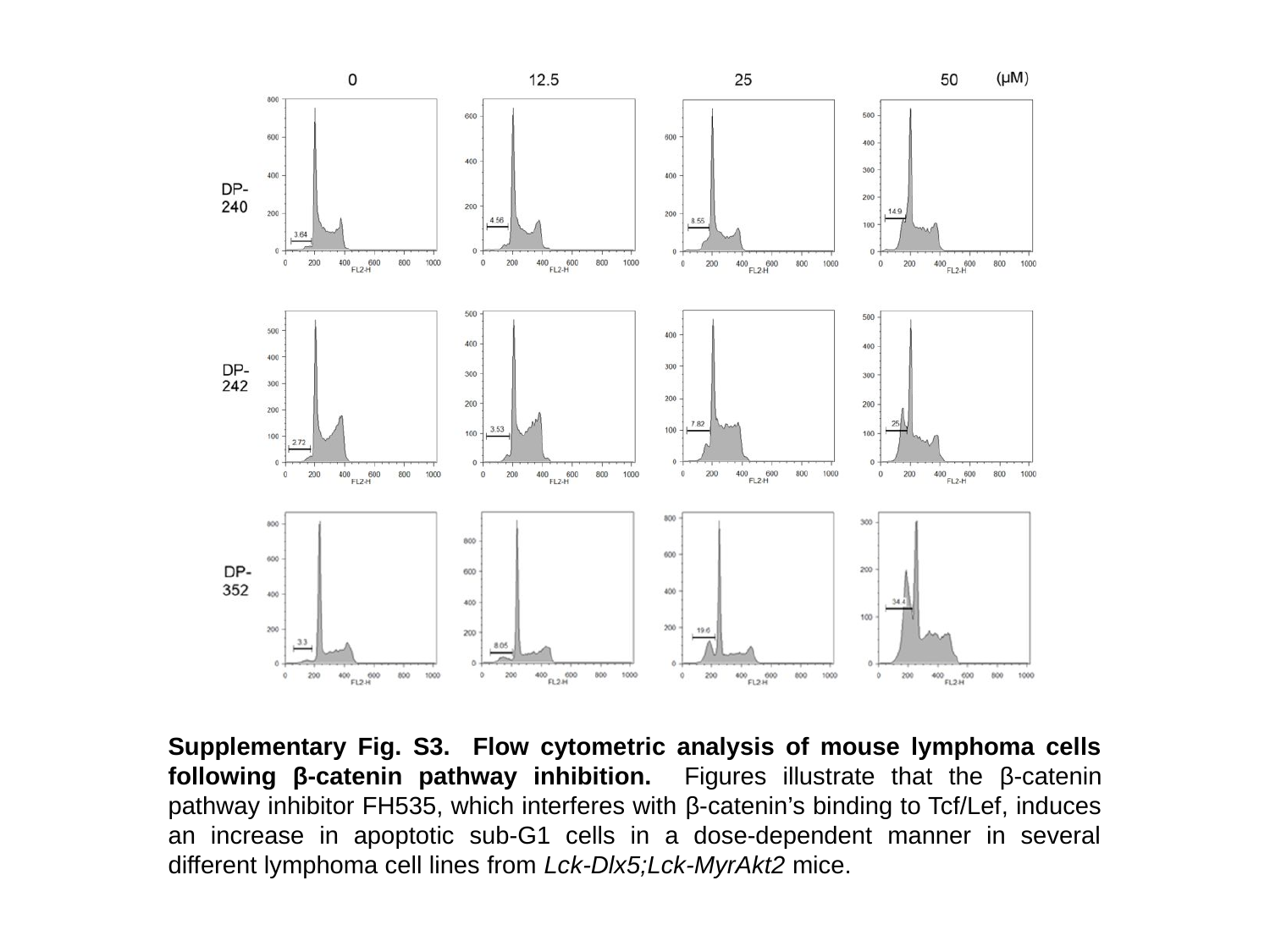

Supplementary Fig. S3. Flow cytometric analysis of mouse lymphoma cells following β-catenin pathway inhibition. Figures illustrate that the β-catenin pathway inhibitor FH535, which interferes with β-catenin’s binding to Tcf/Lef, induces an increase in apoptotic sub-G1 cells in a dose-dependent manner in several different lymphoma cell lines from Lck-Dlx5;Lck-MyrAkt2 mice.

## Slide 6
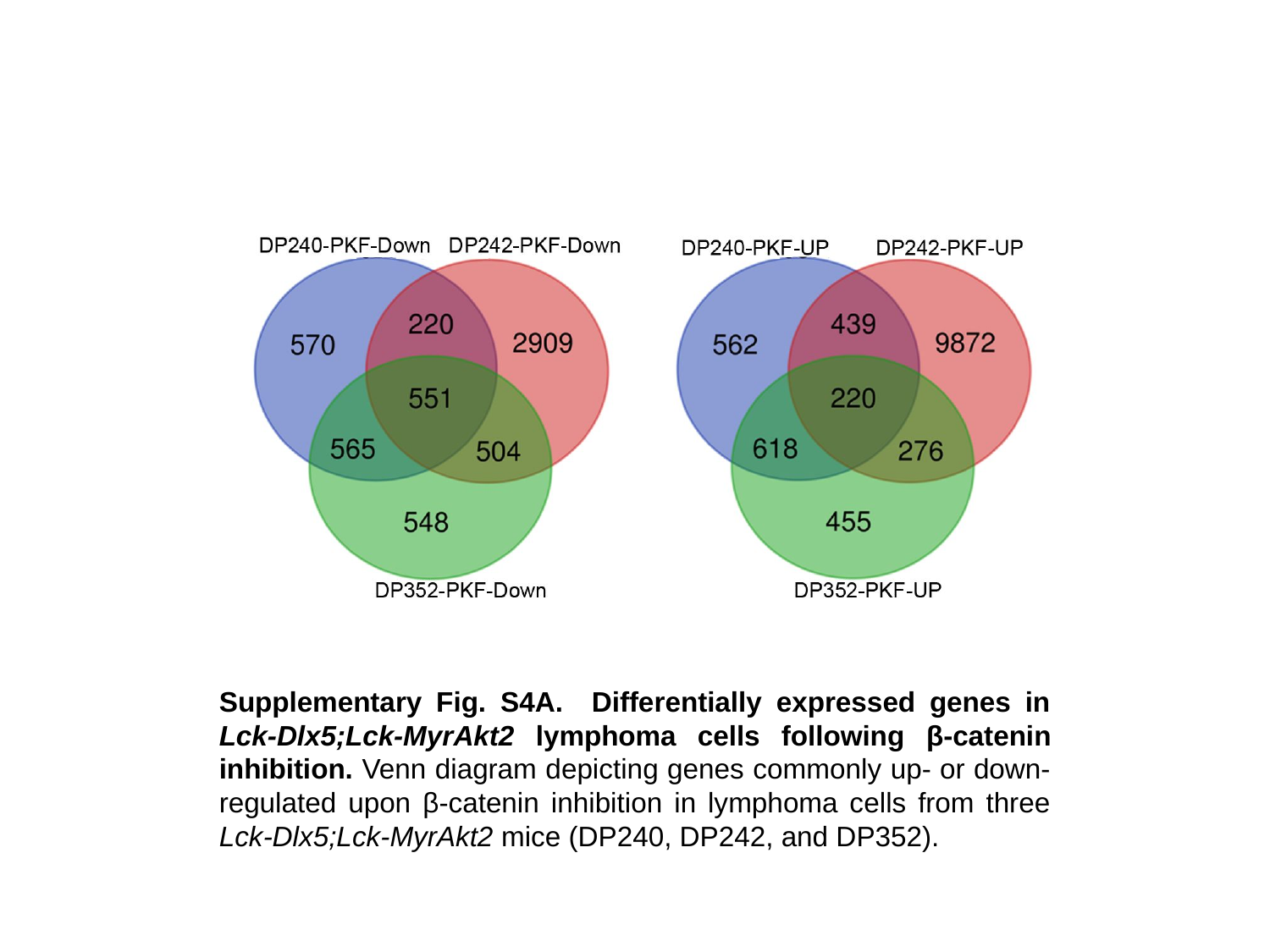

Supplementary Fig. S4A. Differentially expressed genes in Lck-Dlx5;Lck-MyrAkt2 lymphoma cells following β-catenin inhibition. Venn diagram depicting genes commonly up- or down-regulated upon β-catenin inhibition in lymphoma cells from three Lck-Dlx5;Lck-MyrAkt2 mice (DP240, DP242, and DP352).

## Slide 7
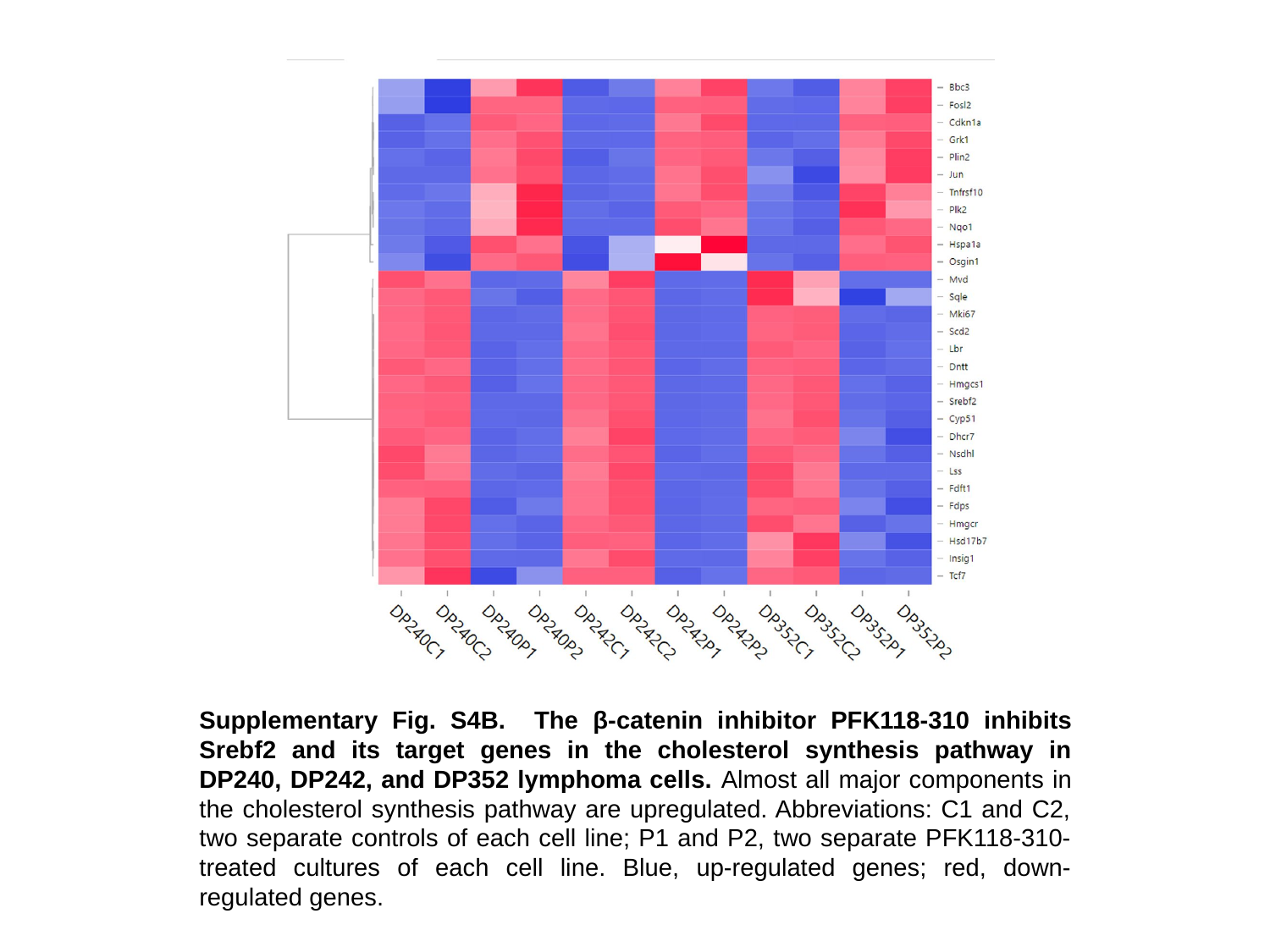

Supplementary Fig. S4B. The β-catenin inhibitor PFK118-310 inhibits Srebf2 and its target genes in the cholesterol synthesis pathway in DP240, DP242, and DP352 lymphoma cells. Almost all major components in the cholesterol synthesis pathway are upregulated. Abbreviations: C1 and C2, two separate controls of each cell line; P1 and P2, two separate PFK118-310-treated cultures of each cell line. Blue, up-regulated genes; red, down-regulated genes.

## Slide 8
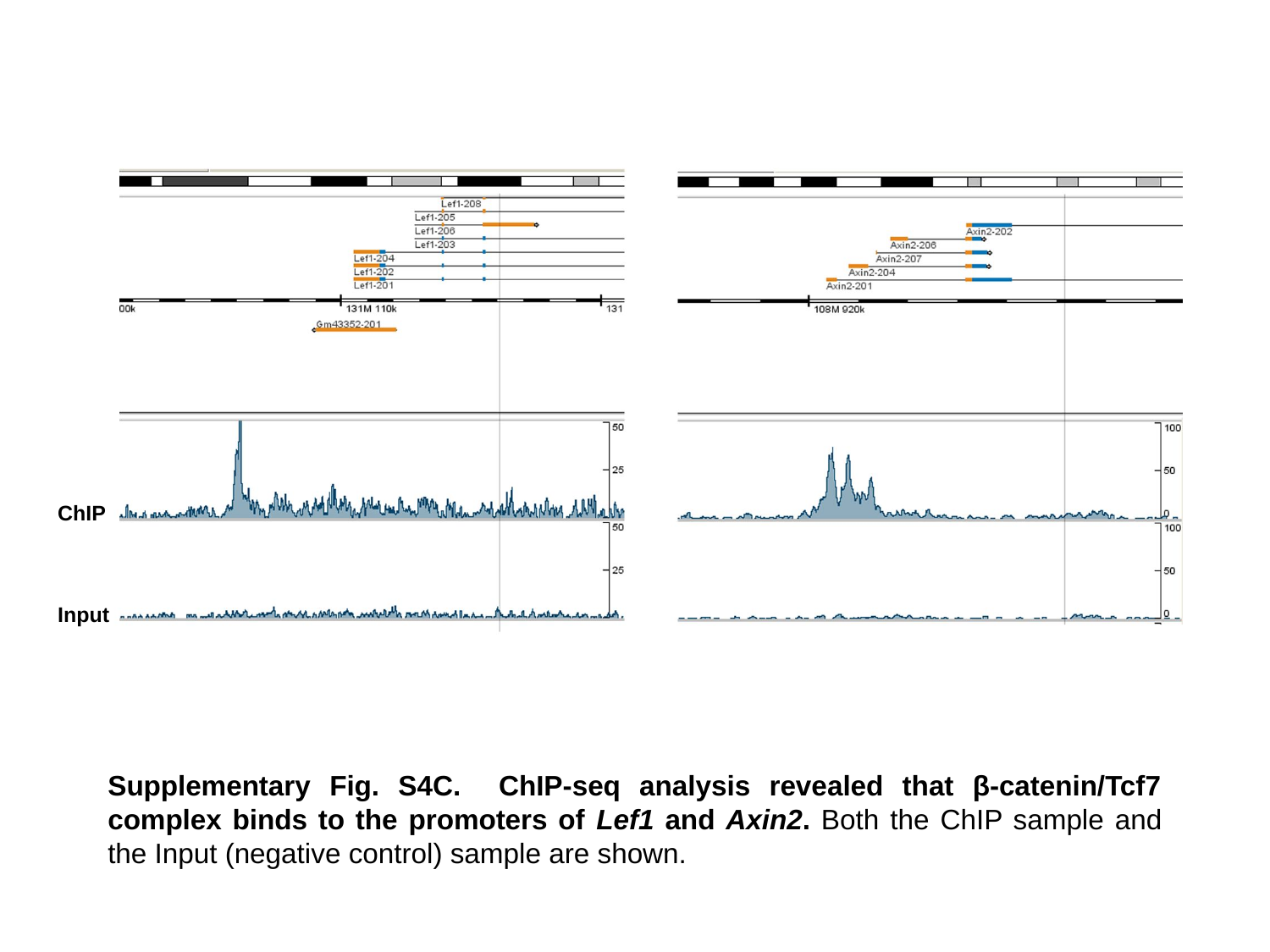

ChIP
Input
Supplementary Fig. S4C. ChIP-seq analysis revealed that β-catenin/Tcf7 complex binds to the promoters of Lef1 and Axin2. Both the ChIP sample and the Input (negative control) sample are shown.

## Slide 9
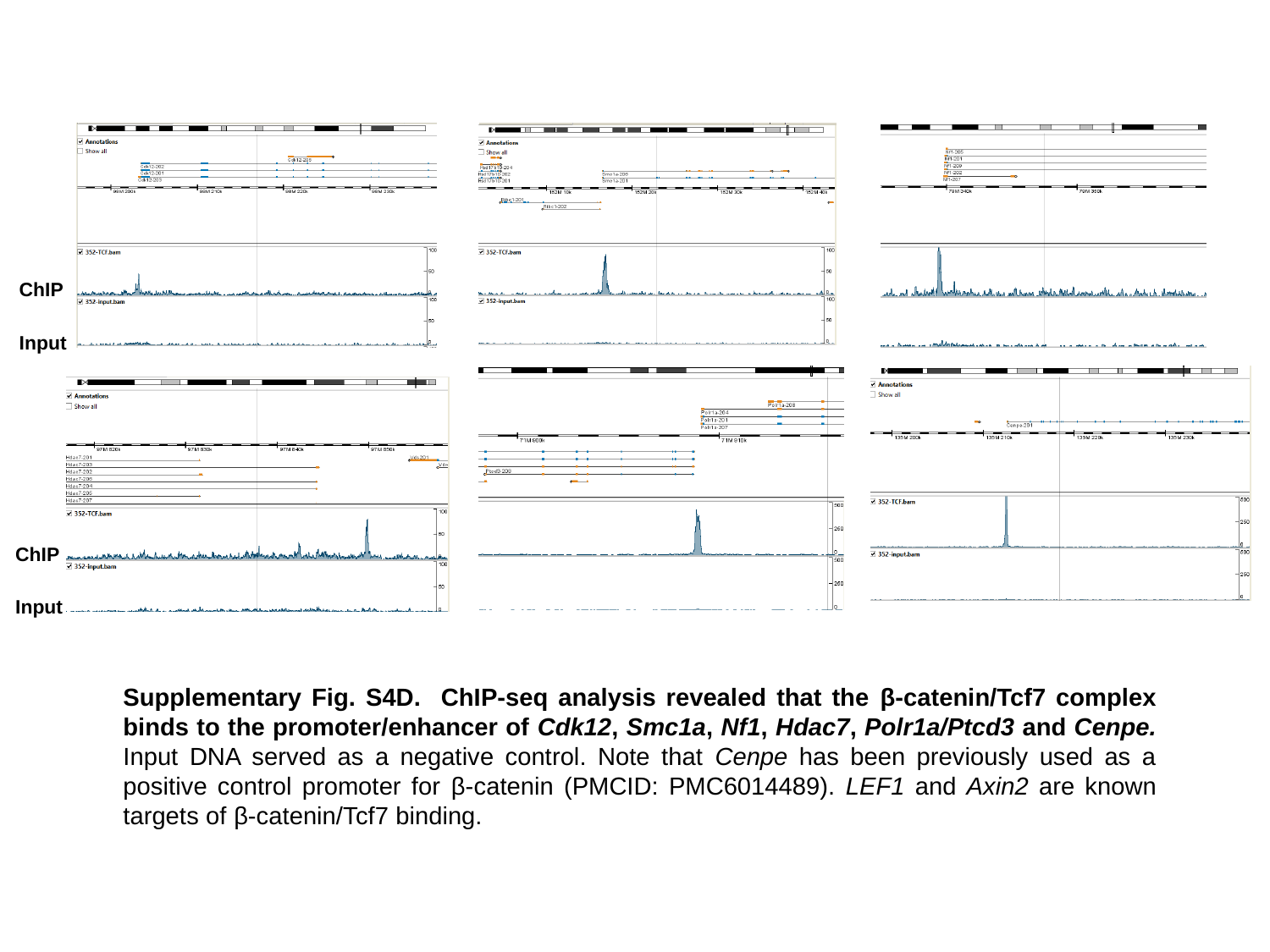

ChIP
Input
ChIP
Input
Supplementary Fig. S4D. ChIP-seq analysis revealed that the β-catenin/Tcf7 complex binds to the promoter/enhancer of Cdk12, Smc1a, Nf1, Hdac7, Polr1a/Ptcd3 and Cenpe. Input DNA served as a negative control. Note that Cenpe has been previously used as a positive control promoter for β-catenin (PMCID: PMC6014489). LEF1 and Axin2 are known targets of β-catenin/Tcf7 binding.

## Slide 10
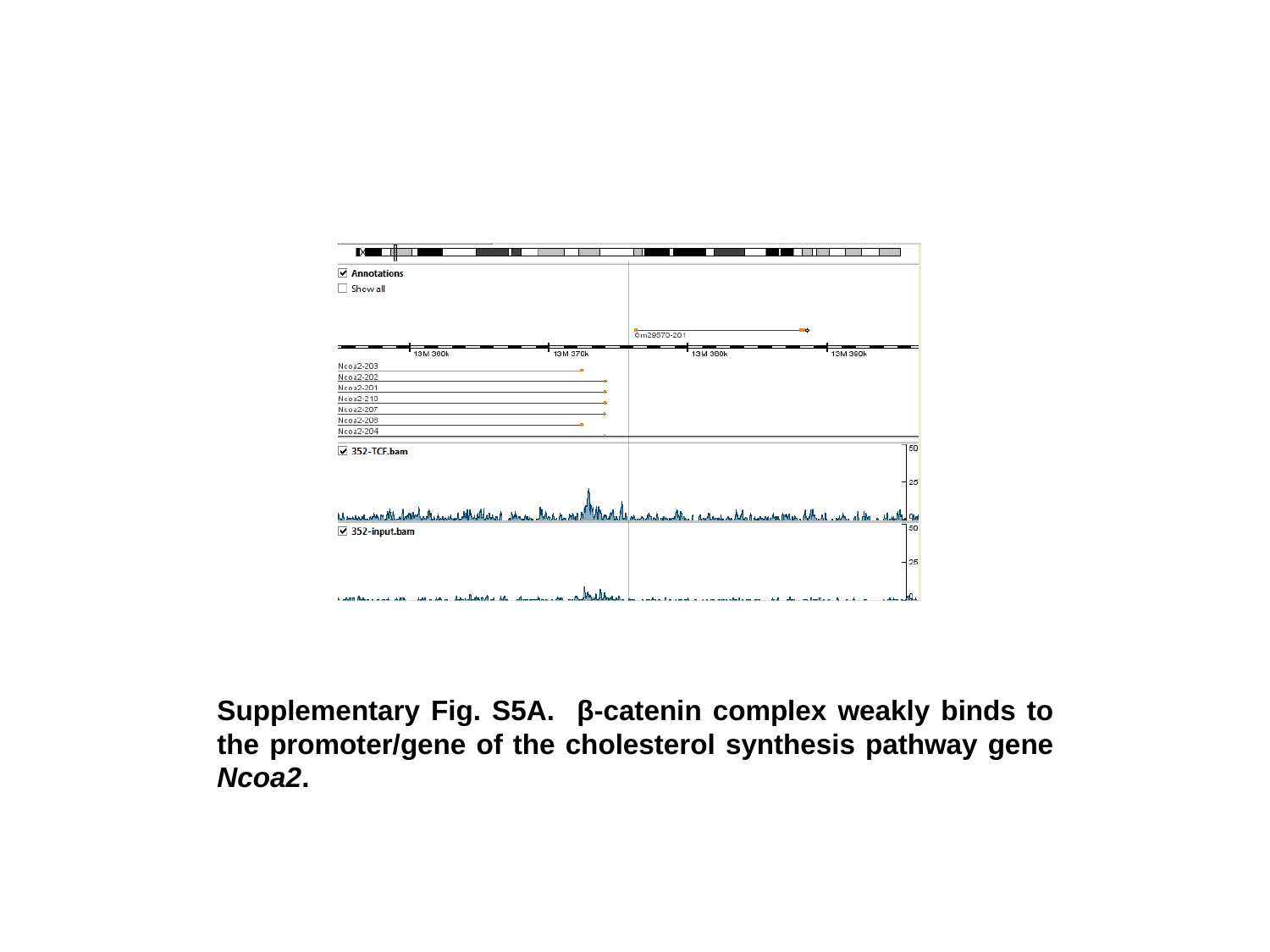

Supplementary Fig. S5A. β-catenin complex weakly binds to the promoter/gene of the cholesterol synthesis pathway gene Ncoa2.

## Slide 11
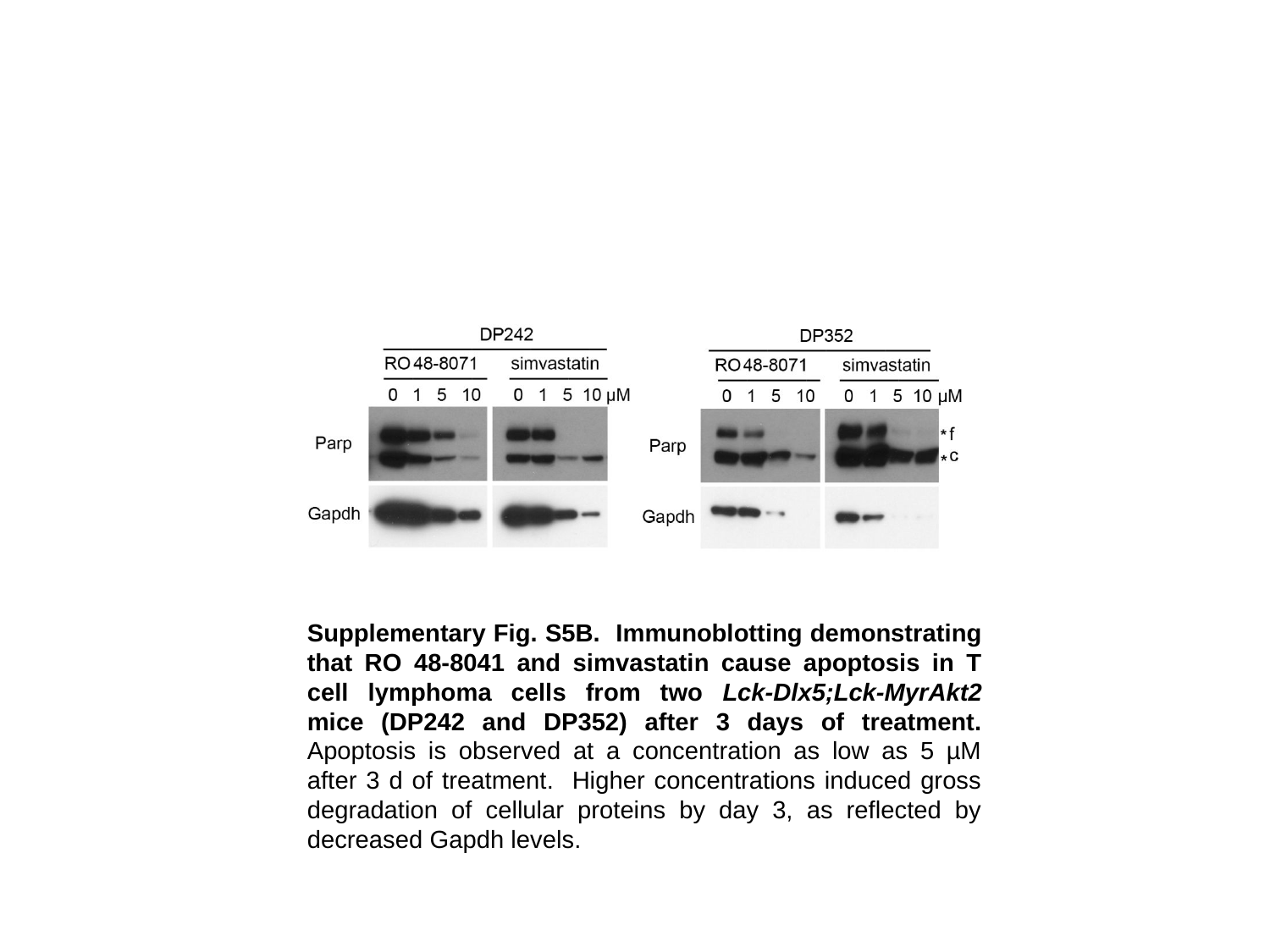

Supplementary Fig. S5B. Immunoblotting demonstrating that RO 48-8041 and simvastatin cause apoptosis in T cell lymphoma cells from two Lck-Dlx5;Lck-MyrAkt2 mice (DP242 and DP352) after 3 days of treatment. Apoptosis is observed at a concentration as low as 5 µM after 3 d of treatment. Higher concentrations induced gross degradation of cellular proteins by day 3, as reflected by decreased Gapdh levels.

## Slide 12
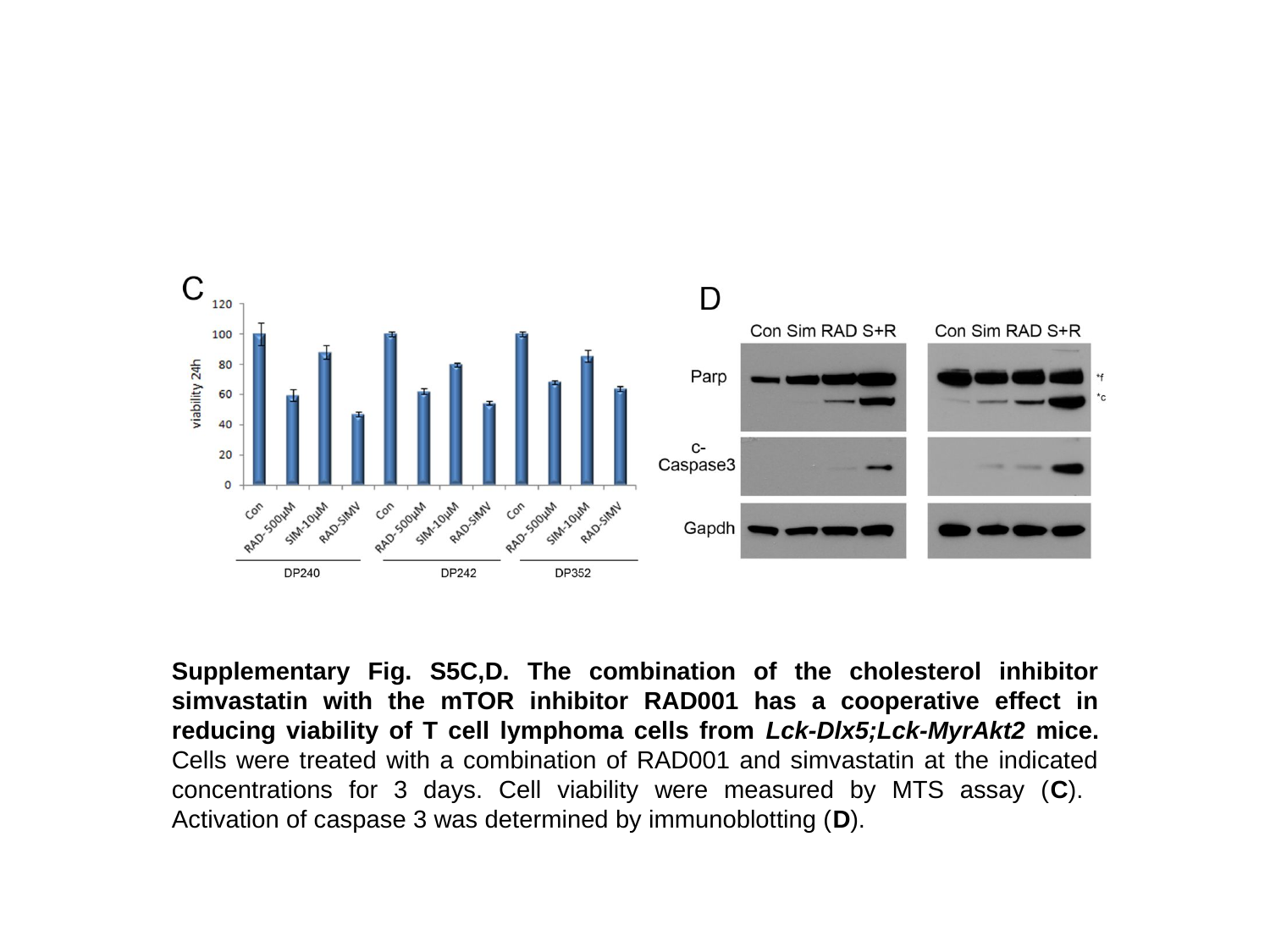

Supplementary Fig. S5C,D. The combination of the cholesterol inhibitor simvastatin with the mTOR inhibitor RAD001 has a cooperative effect in reducing viability of T cell lymphoma cells from Lck-Dlx5;Lck-MyrAkt2 mice. Cells were treated with a combination of RAD001 and simvastatin at the indicated concentrations for 3 days. Cell viability were measured by MTS assay (C). Activation of caspase 3 was determined by immunoblotting (D).

## Slide 13
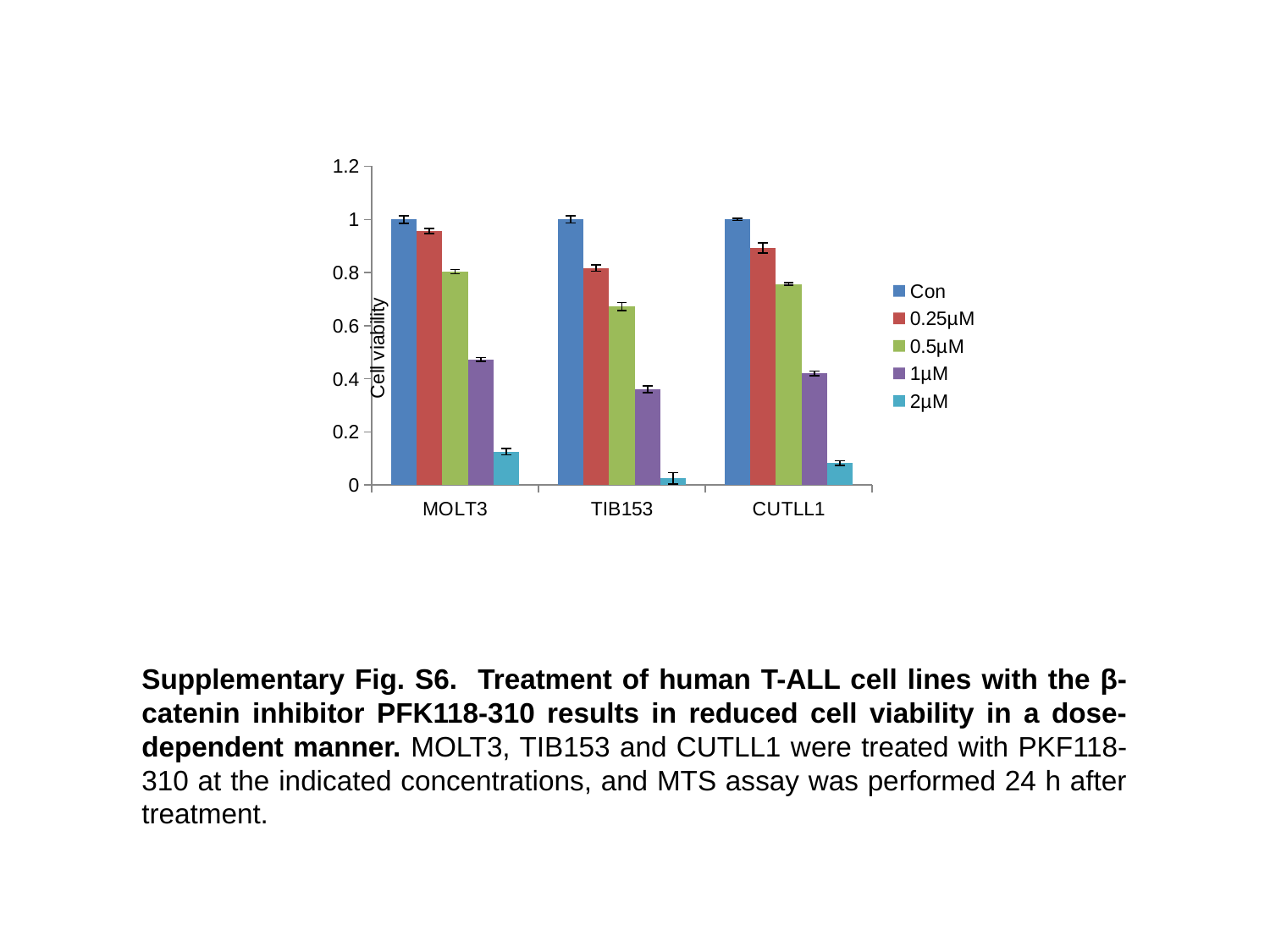

### Chart
| Category | Con | 0.25µM | 0.5µM | 1µM | 2µM |
|---|---|---|---|---|---|
| MOLT3 | 1.0 | 0.9561671763506625 | 0.8032619775739042 | 0.4729867482161061 | 0.1253822629969419 |
| TIB153 | 1.0 | 0.8168273444347063 | 0.6722173531989483 | 0.3602103418054338 | 0.025416301489921144 |
| CUTLL1 | 1.0 | 0.8928188638799571 | 0.7566988210075026 | 0.4201500535905681 | 0.08252947481243299 |Supplementary Fig. S6. Treatment of human T-ALL cell lines with the β-catenin inhibitor PFK118-310 results in reduced cell viability in a dose-dependent manner. MOLT3, TIB153 and CUTLL1 were treated with PKF118-310 at the indicated concentrations, and MTS assay was performed 24 h after treatment.

## Slide 14
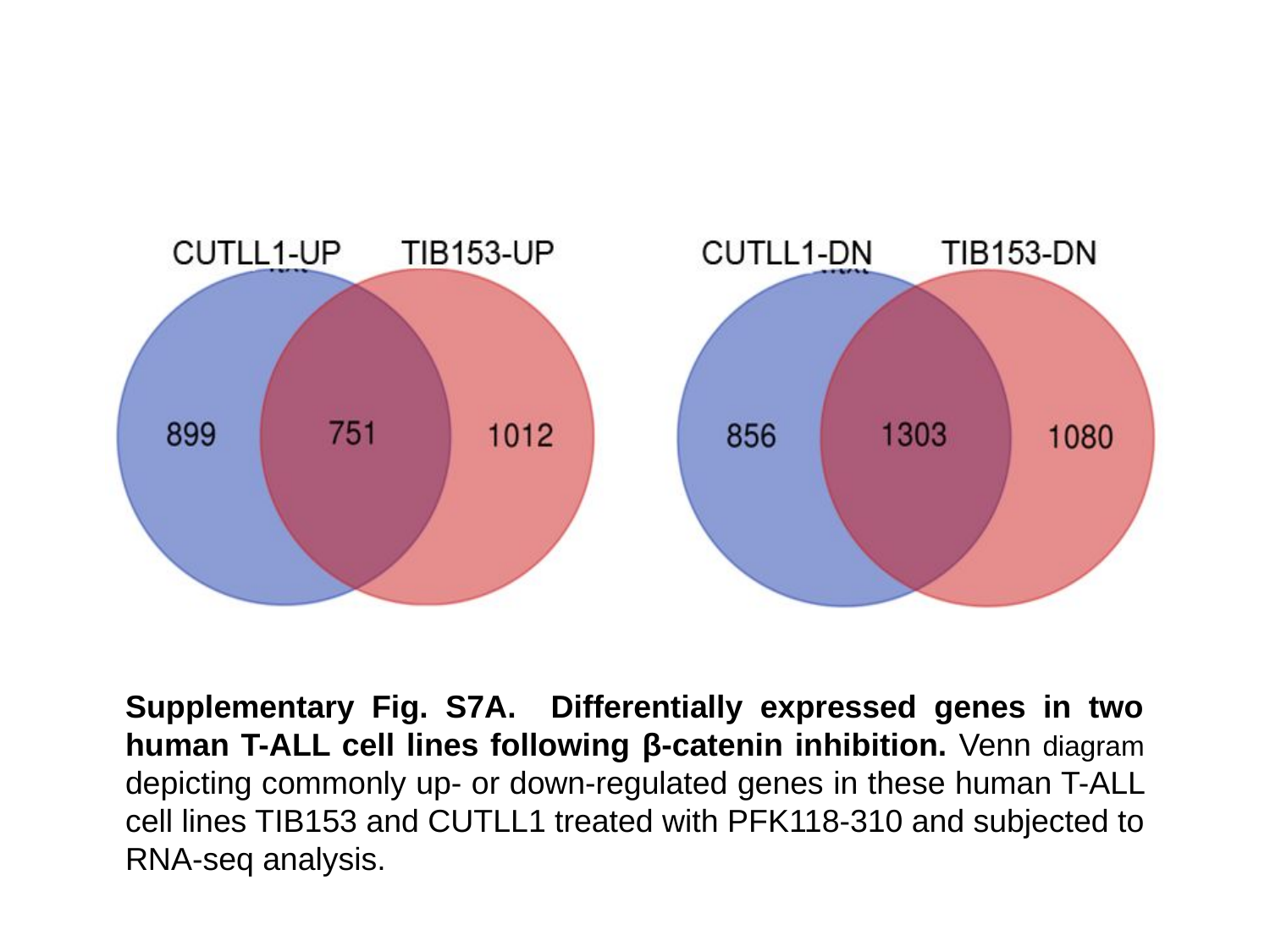

Supplementary Fig. S7A. Differentially expressed genes in two human T-ALL cell lines following β-catenin inhibition. Venn diagram depicting commonly up- or down-regulated genes in these human T-ALL cell lines TIB153 and CUTLL1 treated with PFK118-310 and subjected to RNA-seq analysis.

## Slide 15
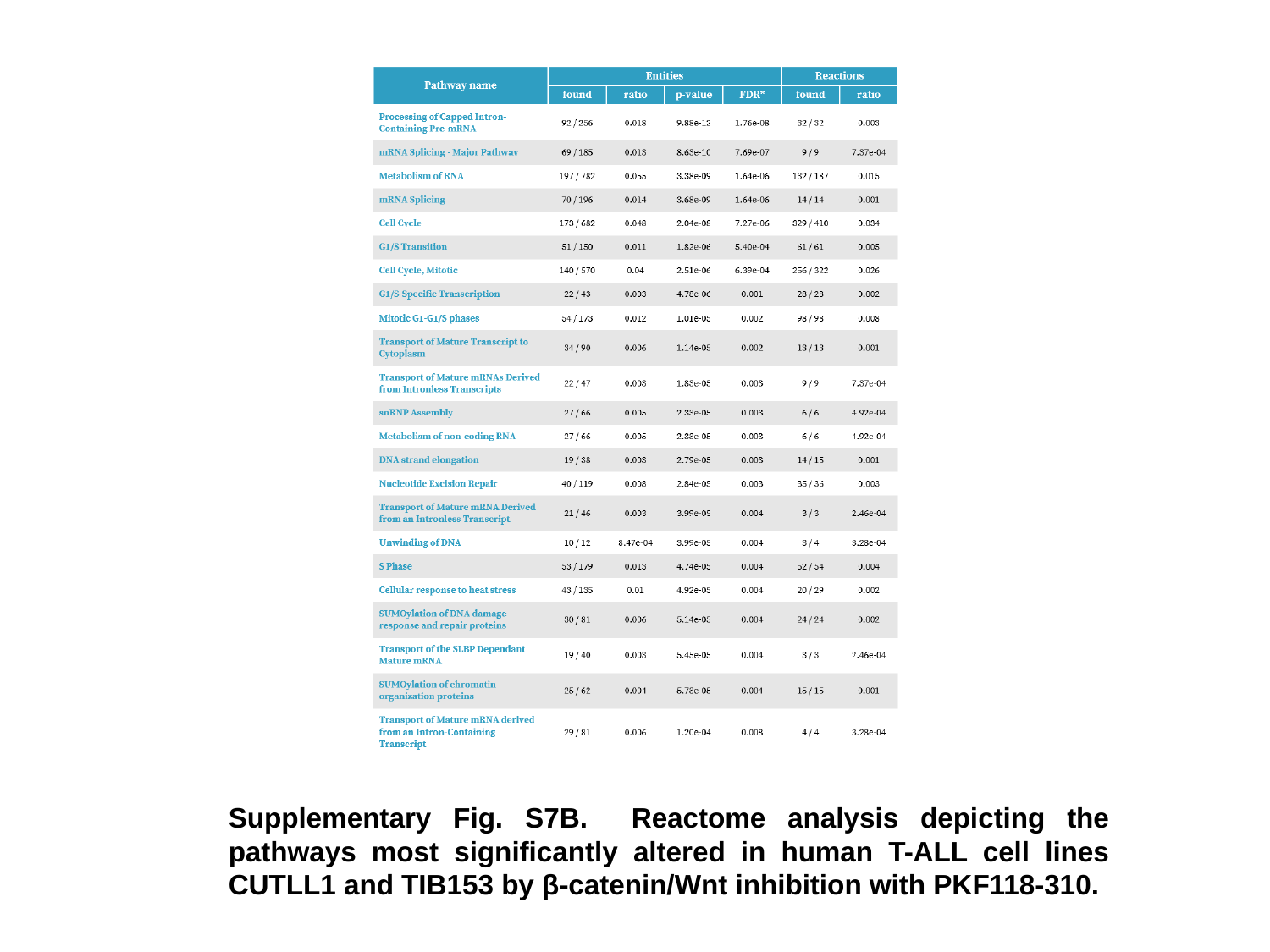

Supplementary Fig. S7B. Reactome analysis depicting the pathways most significantly altered in human T-ALL cell lines CUTLL1 and TIB153 by β-catenin/Wnt inhibition with PKF118-310.

## Slide 16
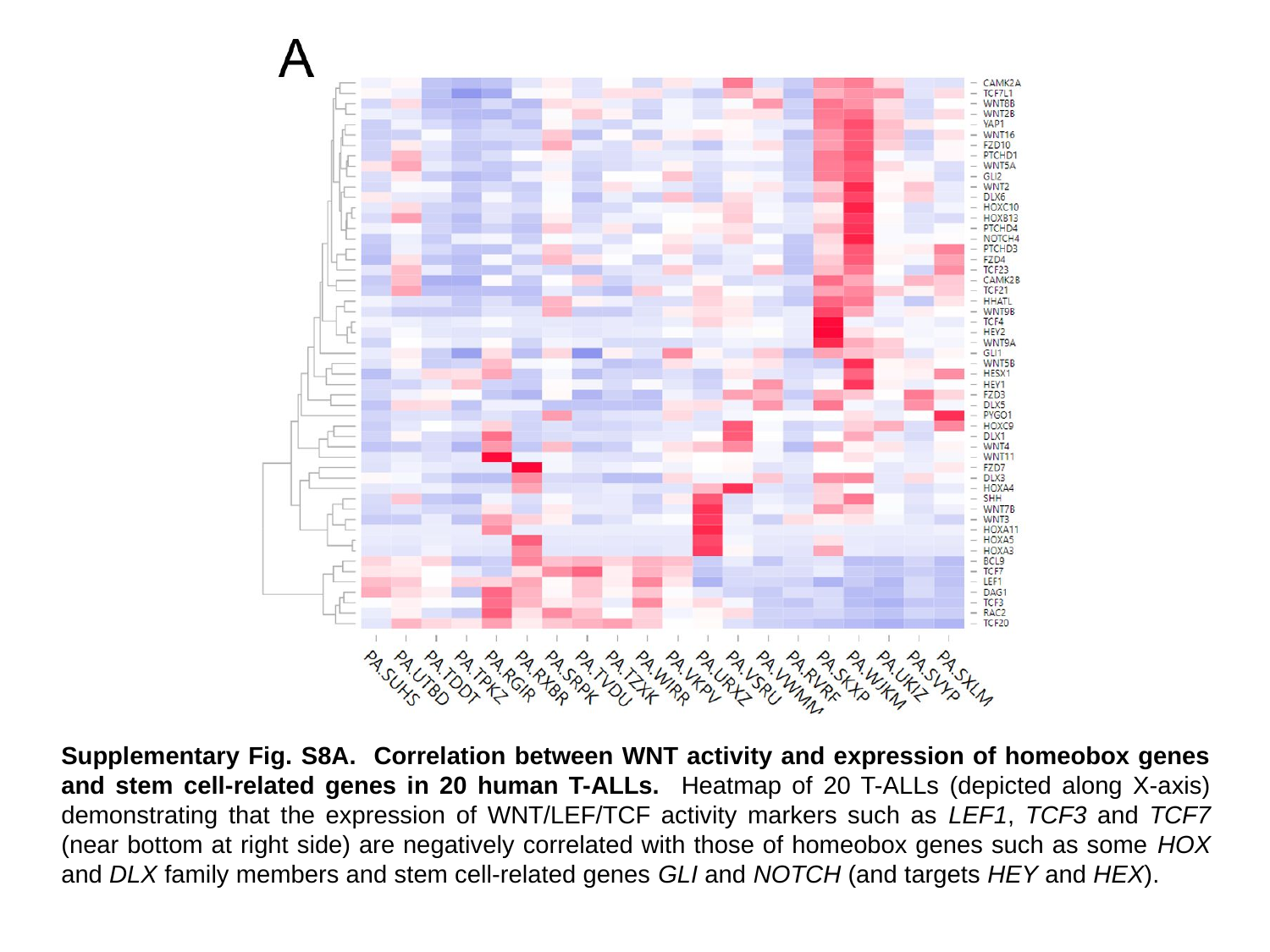

Supplementary Fig. S8A. Correlation between WNT activity and expression of homeobox genes and stem cell-related genes in 20 human T-ALLs. Heatmap of 20 T-ALLs (depicted along X-axis) demonstrating that the expression of WNT/LEF/TCF activity markers such as LEF1, TCF3 and TCF7 (near bottom at right side) are negatively correlated with those of homeobox genes such as some HOX and DLX family members and stem cell-related genes GLI and NOTCH (and targets HEY and HEX).

## Slide 17
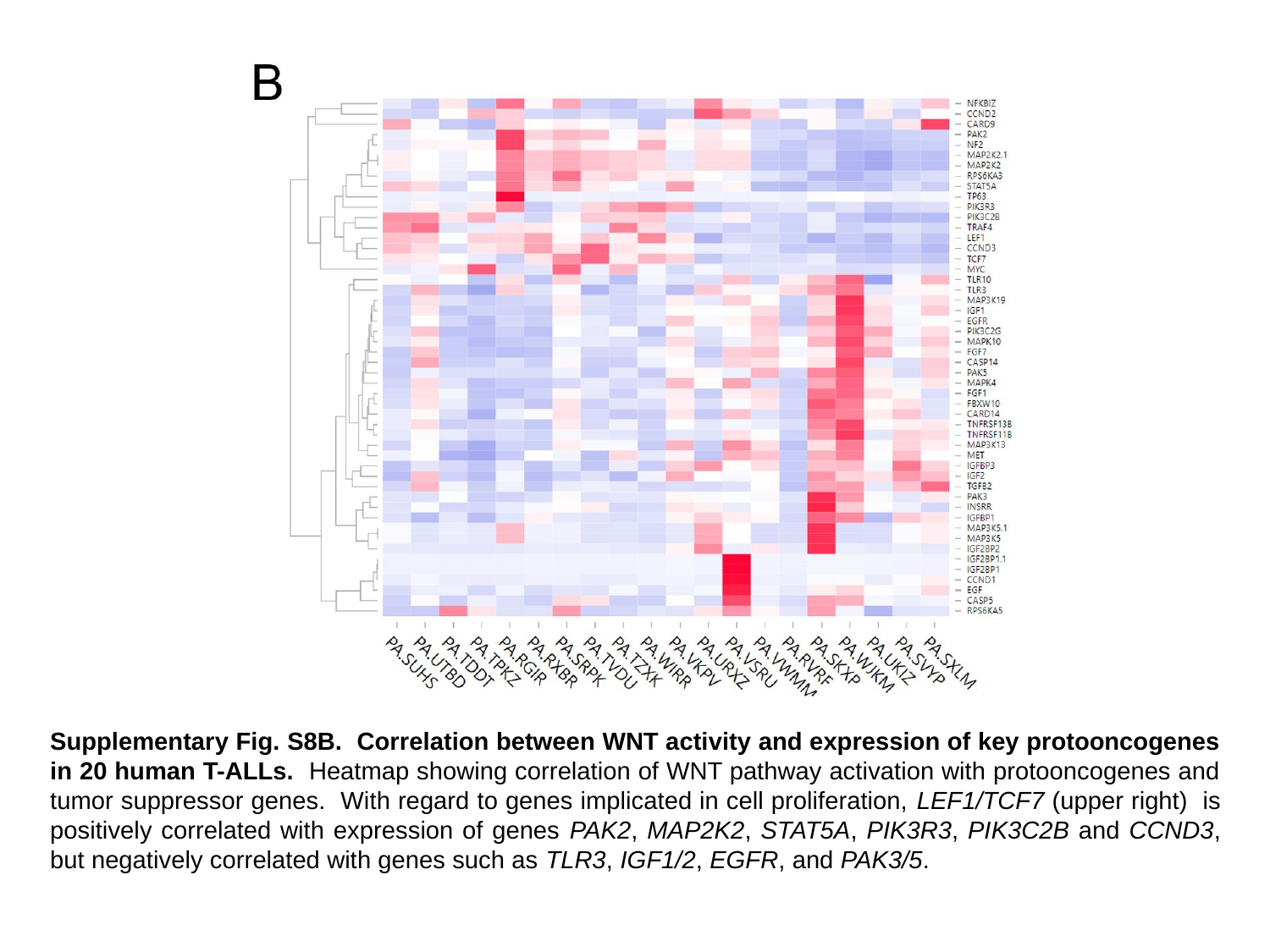

Supplementary Fig. S8B. Correlation between WNT activity and expression of key protooncogenes in 20 human T-ALLs. Heatmap showing correlation of WNT pathway activation with protooncogenes and tumor suppressor genes. With regard to genes implicated in cell proliferation, LEF1/TCF7 (upper right) is positively correlated with expression of genes PAK2, MAP2K2, STAT5A, PIK3R3, PIK3C2B and CCND3, but negatively correlated with genes such as TLR3, IGF1/2, EGFR, and PAK3/5.

## Slide 18
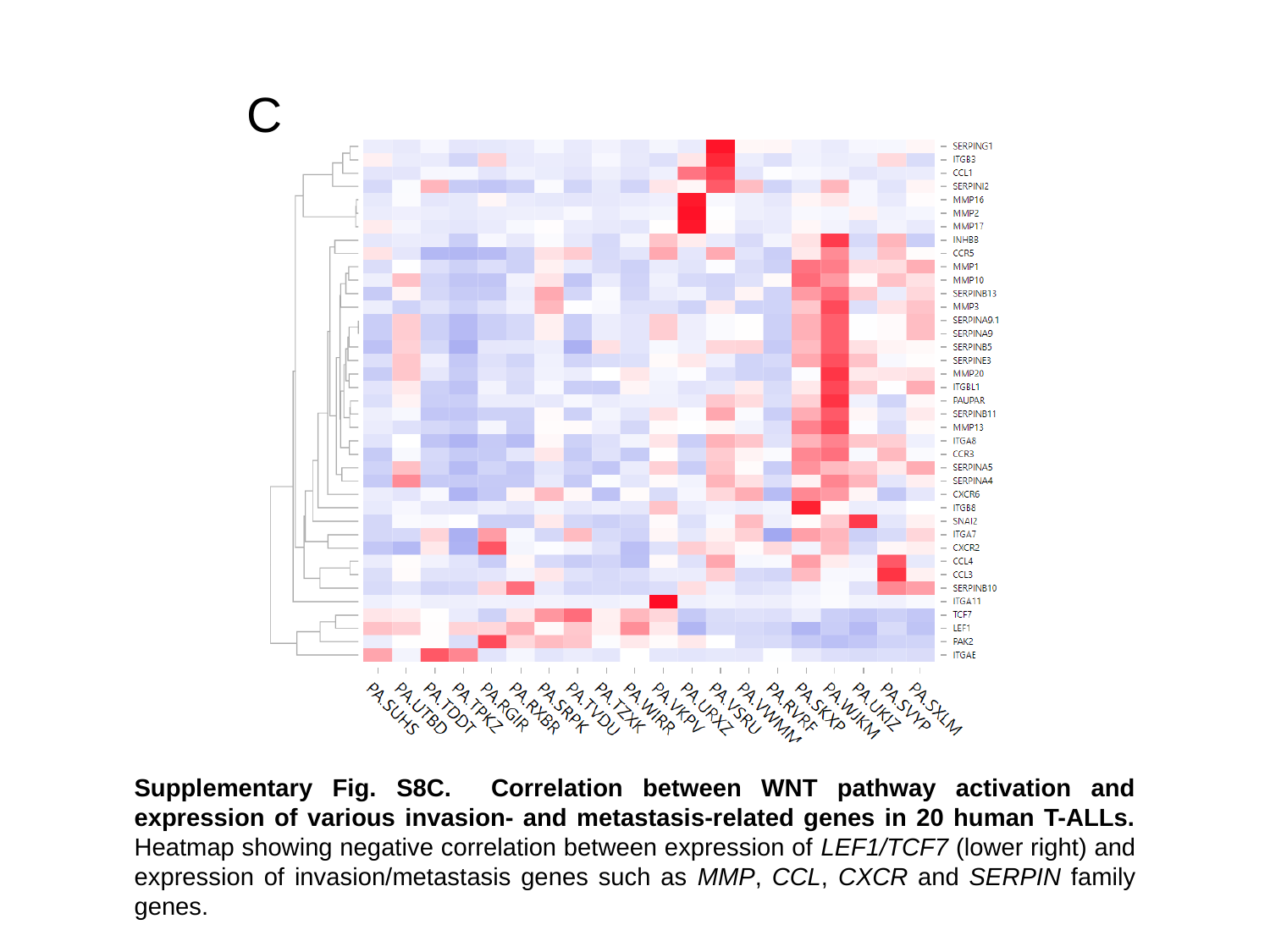

# C
Supplementary Fig. S8C. Correlation between WNT pathway activation and expression of various invasion- and metastasis-related genes in 20 human T-ALLs. Heatmap showing negative correlation between expression of LEF1/TCF7 (lower right) and expression of invasion/metastasis genes such as MMP, CCL, CXCR and SERPIN family genes.

## Slide 19
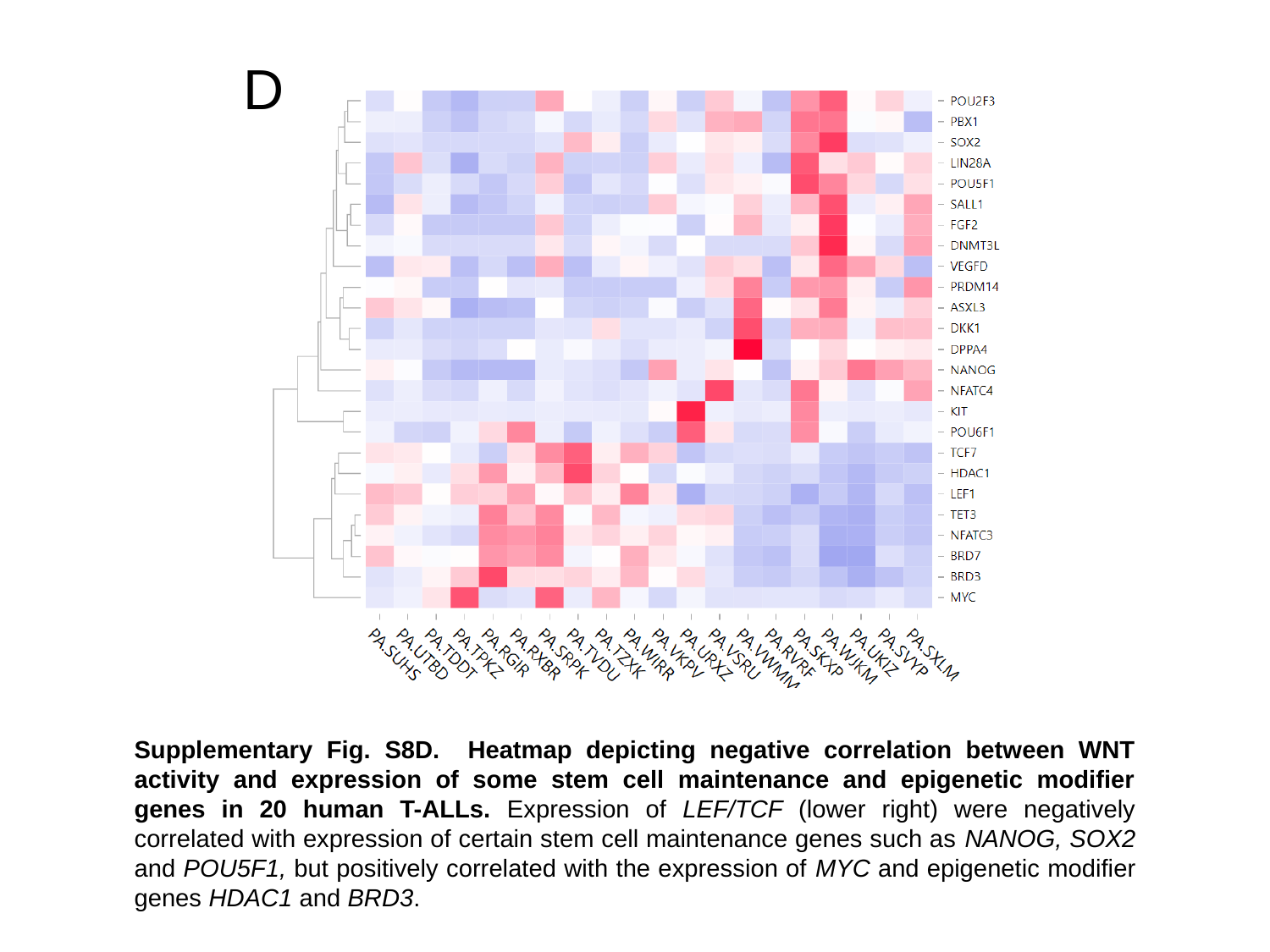

# D
Supplementary Fig. S8D. Heatmap depicting negative correlation between WNT activity and expression of some stem cell maintenance and epigenetic modifier genes in 20 human T-ALLs. Expression of LEF/TCF (lower right) were negatively correlated with expression of certain stem cell maintenance genes such as NANOG, SOX2 and POU5F1, but positively correlated with the expression of MYC and epigenetic modifier genes HDAC1 and BRD3.

## Slide 20
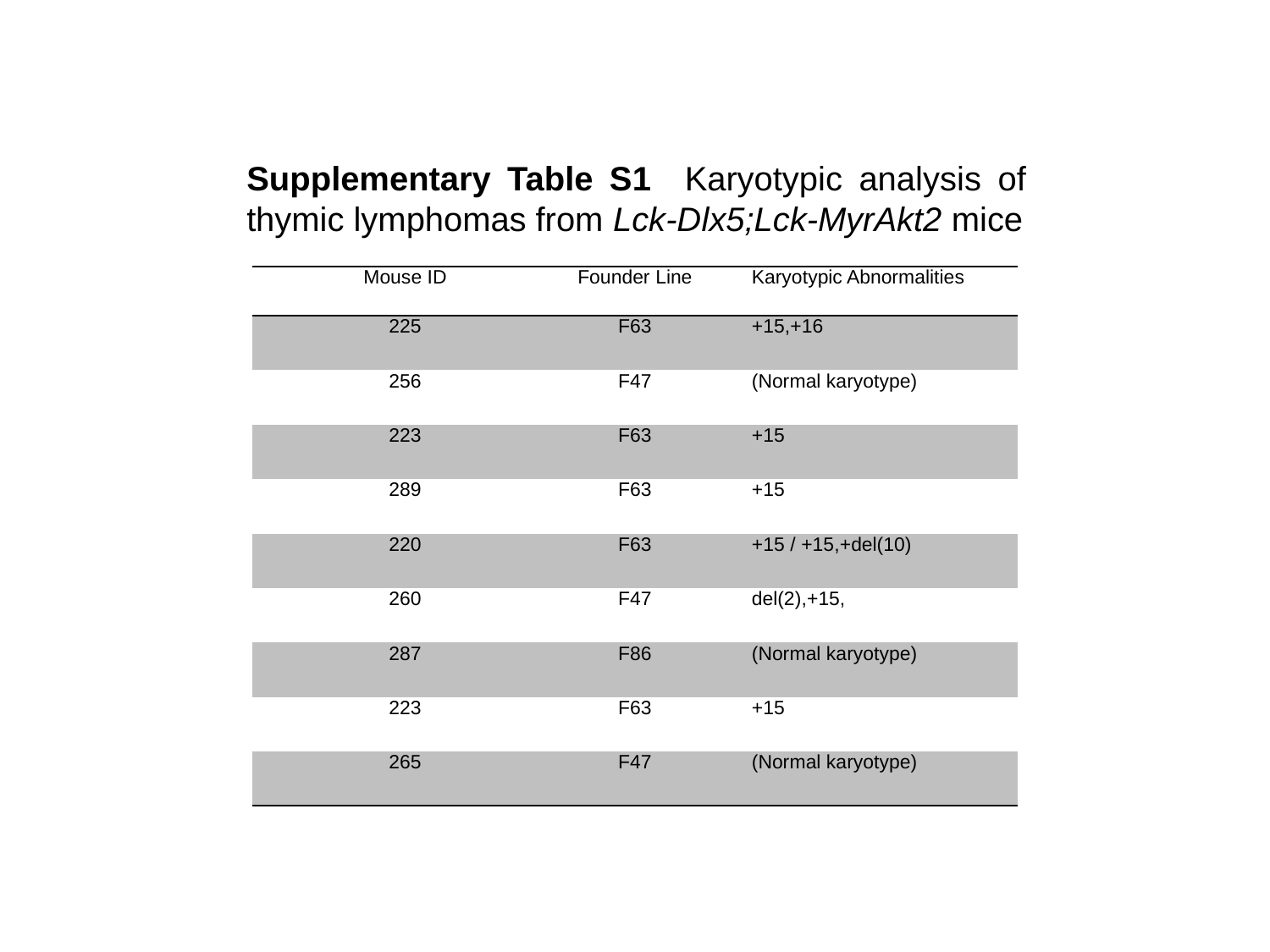

# Supplementary Table S1 Karyotypic analysis of thymic lymphomas from Lck-Dlx5;Lck-MyrAkt2 mice
| Mouse ID | Founder Line | Karyotypic Abnormalities |
| --- | --- | --- |
| 225 | F63 | +15,+16 |
| 256 | F47 | (Normal karyotype) |
| 223 | F63 | +15 |
| 289 | F63 | +15 |
| 220 | F63 | +15 / +15,+del(10) |
| 260 | F47 | del(2),+15, |
| 287 | F86 | (Normal karyotype) |
| 223 | F63 | +15 |
| 265 | F47 | (Normal karyotype) |

## Slide 21
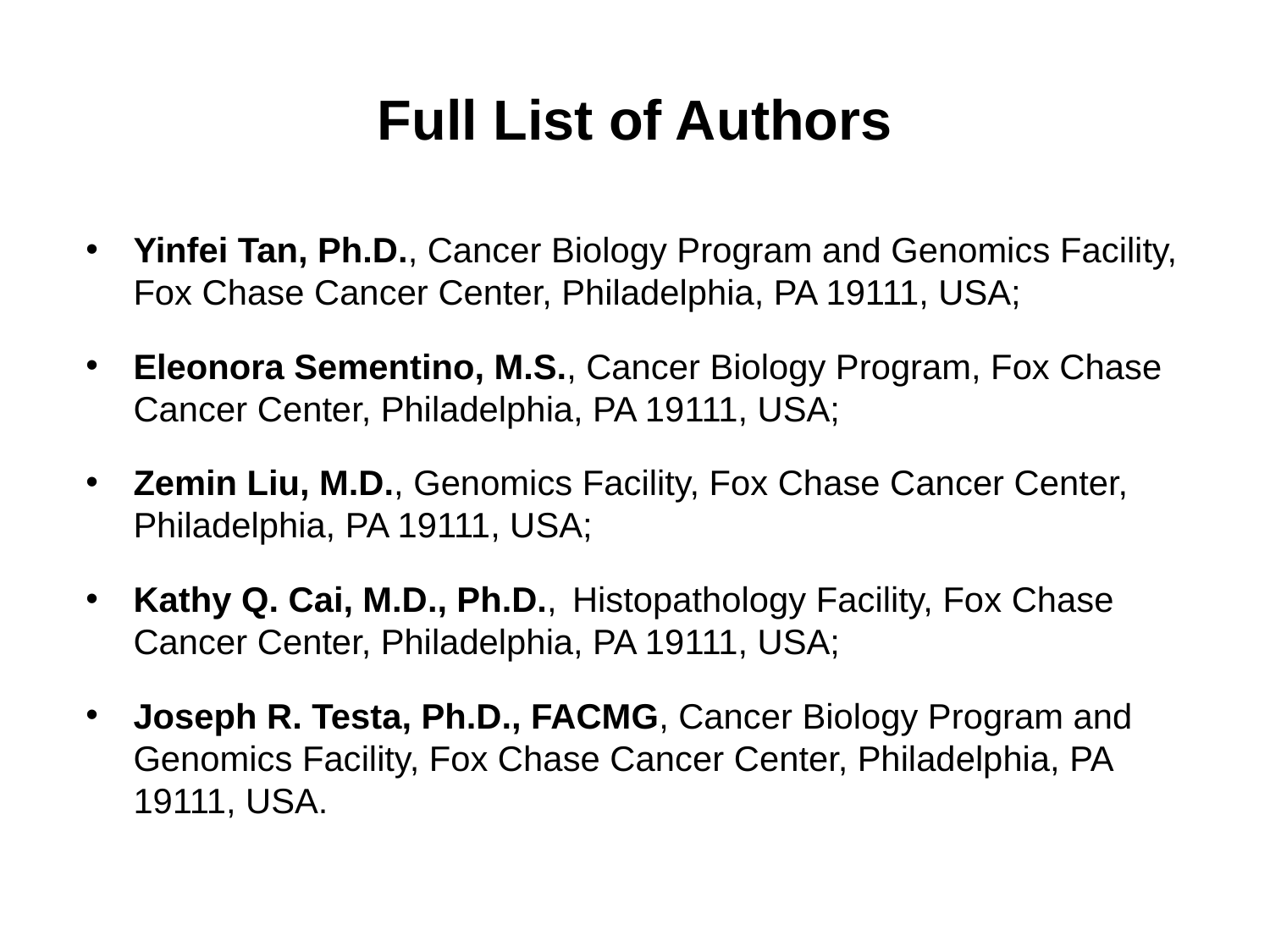

# Full List of Authors
Yinfei Tan, Ph.D., Cancer Biology Program and Genomics Facility, Fox Chase Cancer Center, Philadelphia, PA 19111, USA;
Eleonora Sementino, M.S., Cancer Biology Program, Fox Chase Cancer Center, Philadelphia, PA 19111, USA;
Zemin Liu, M.D., Genomics Facility, Fox Chase Cancer Center, Philadelphia, PA 19111, USA;
Kathy Q. Cai, M.D., Ph.D., Histopathology Facility, Fox Chase Cancer Center, Philadelphia, PA 19111, USA;
Joseph R. Testa, Ph.D., FACMG, Cancer Biology Program and Genomics Facility, Fox Chase Cancer Center, Philadelphia, PA 19111, USA.
