# Supplementary material for: Wnt signaling mediates oncogenic synergy between Akt and Dlx5 in T-cell lymphomagenesis by enhancing cholesterol synthesis
Source: Sci Rep. 2020 Sep 28;10:15837. doi: 10.1038/s41598-020-72822-w (PMC7522078; doi:10.1038/s41598-020-72822-w)
Supplement: Supplementary file 2 — Supplementary information 2. [file 41598_2020_72822_MOESM2_ESM.pdf]

Raw blots

Fig.1C

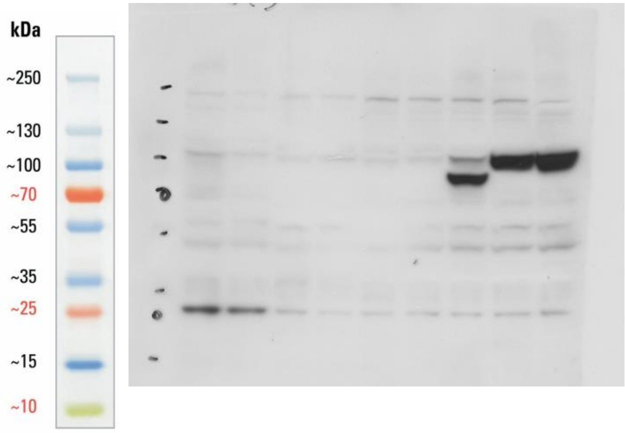

β-Catenin

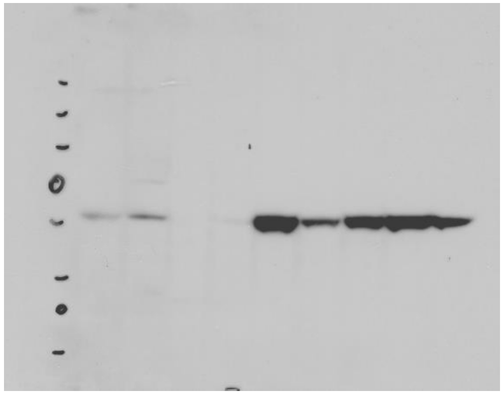

Lef1

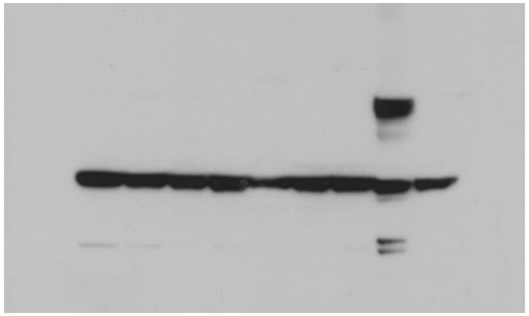

Gsk3β

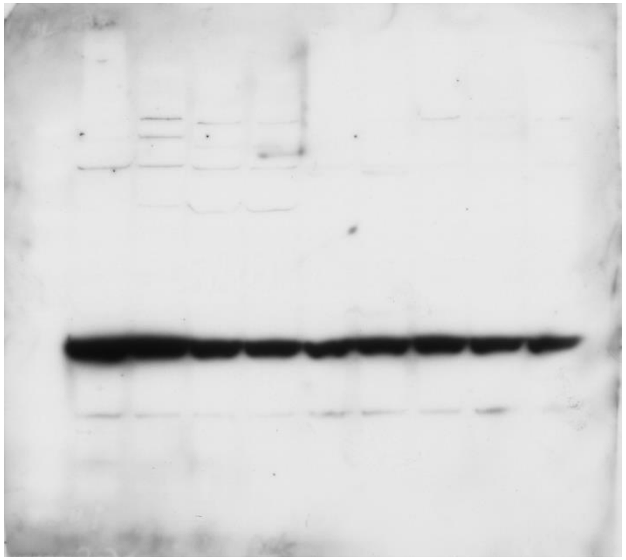

β-Actin

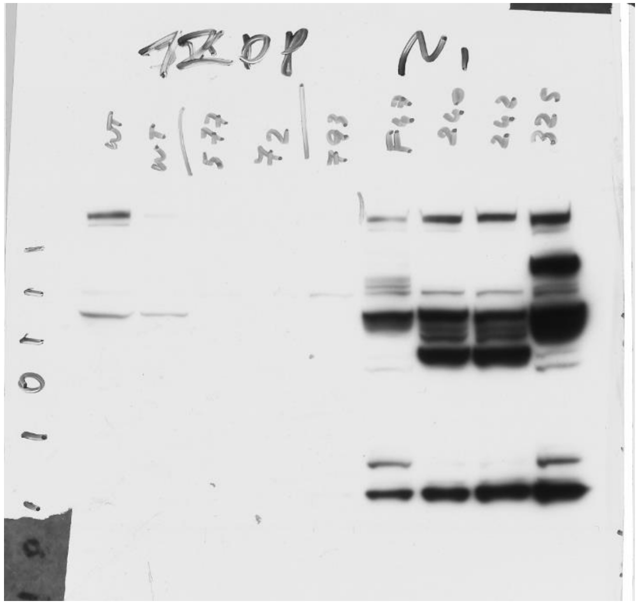

Notch1

Fig.1C- continued

Notch3

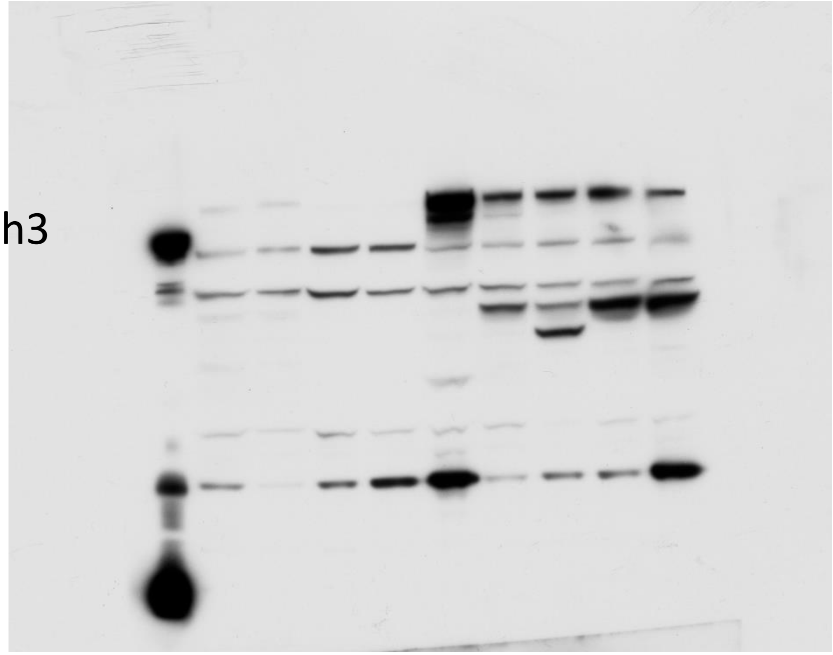

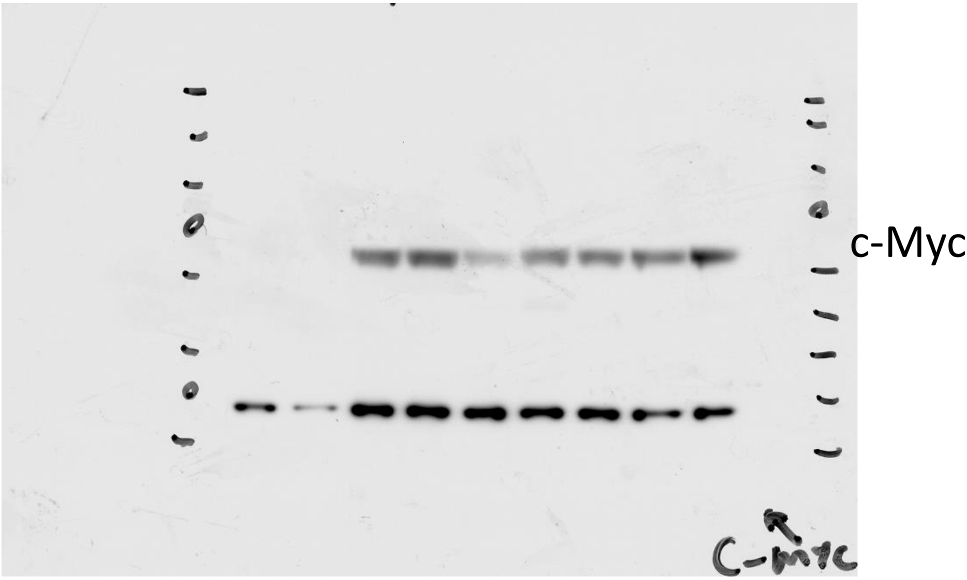

c-Myc

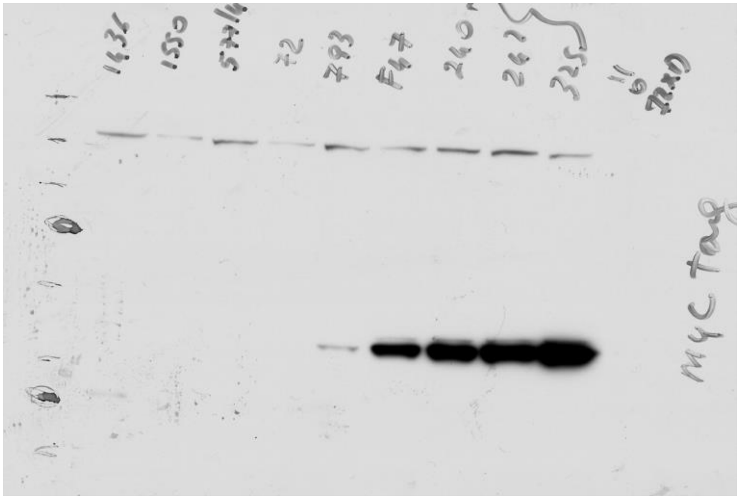

Myc-Tag

Fig.2c

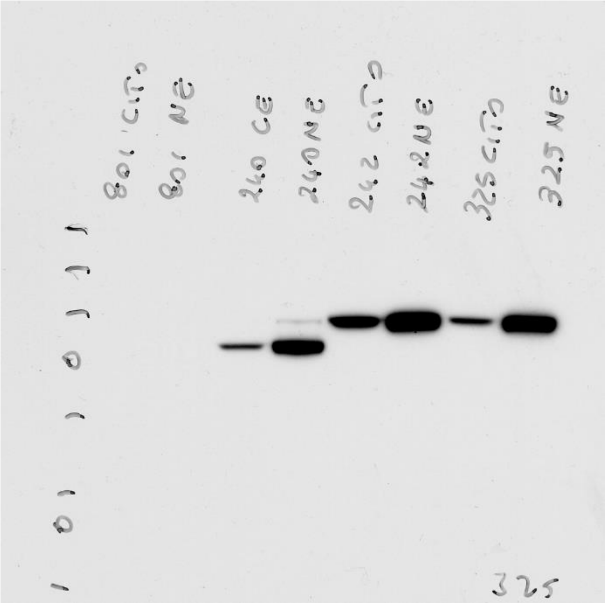

β-Catenin

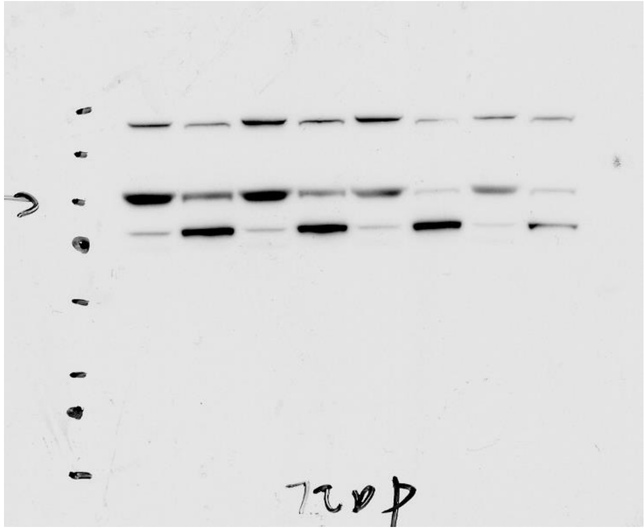

Hsp90&LaminB

Fig.3B

DP240

DP242

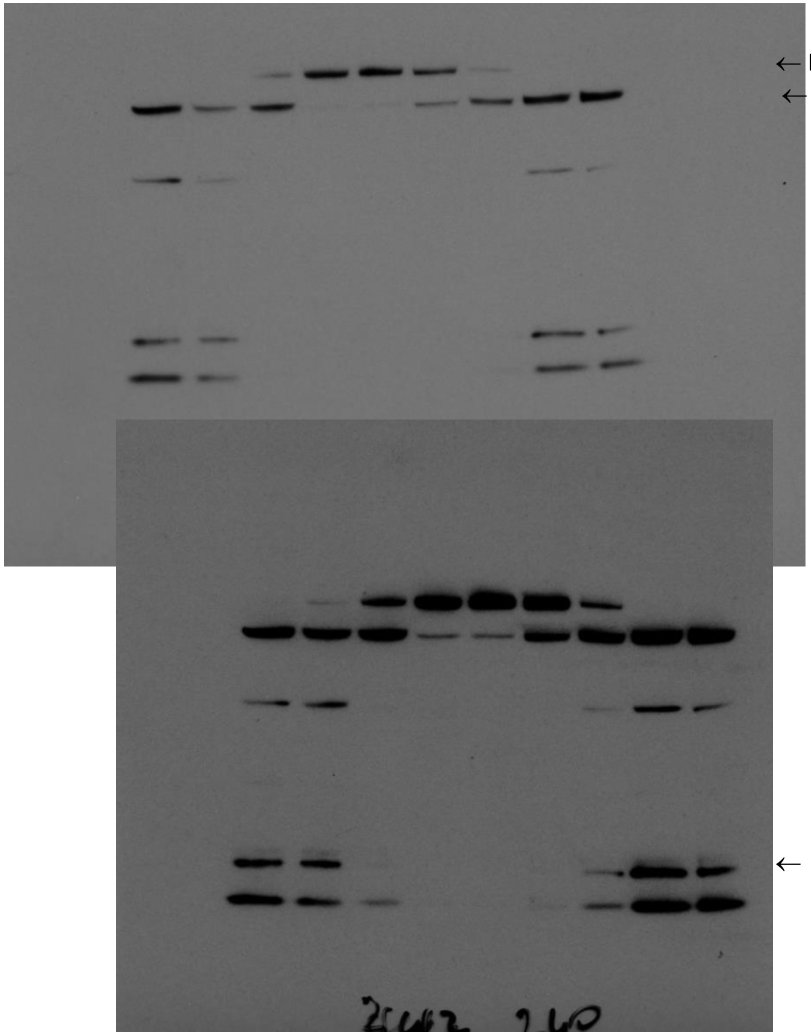

← Full Parp

← cleaved Parp

← Cleaved Caspase3

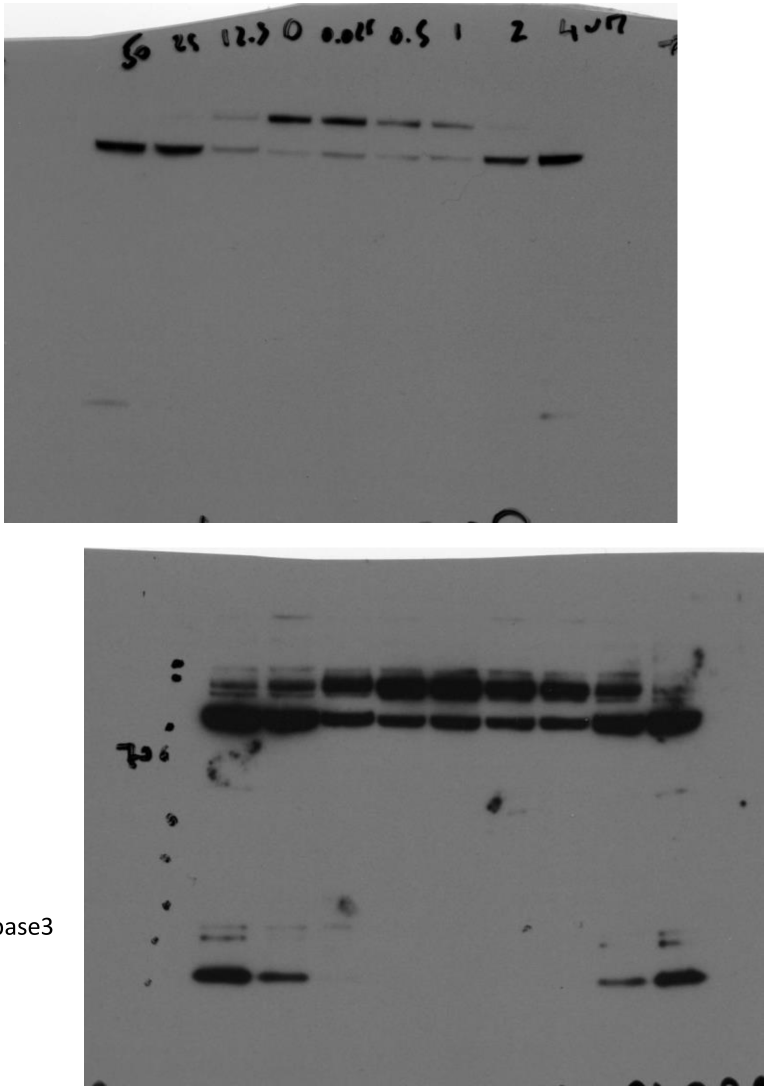

Fig.3B

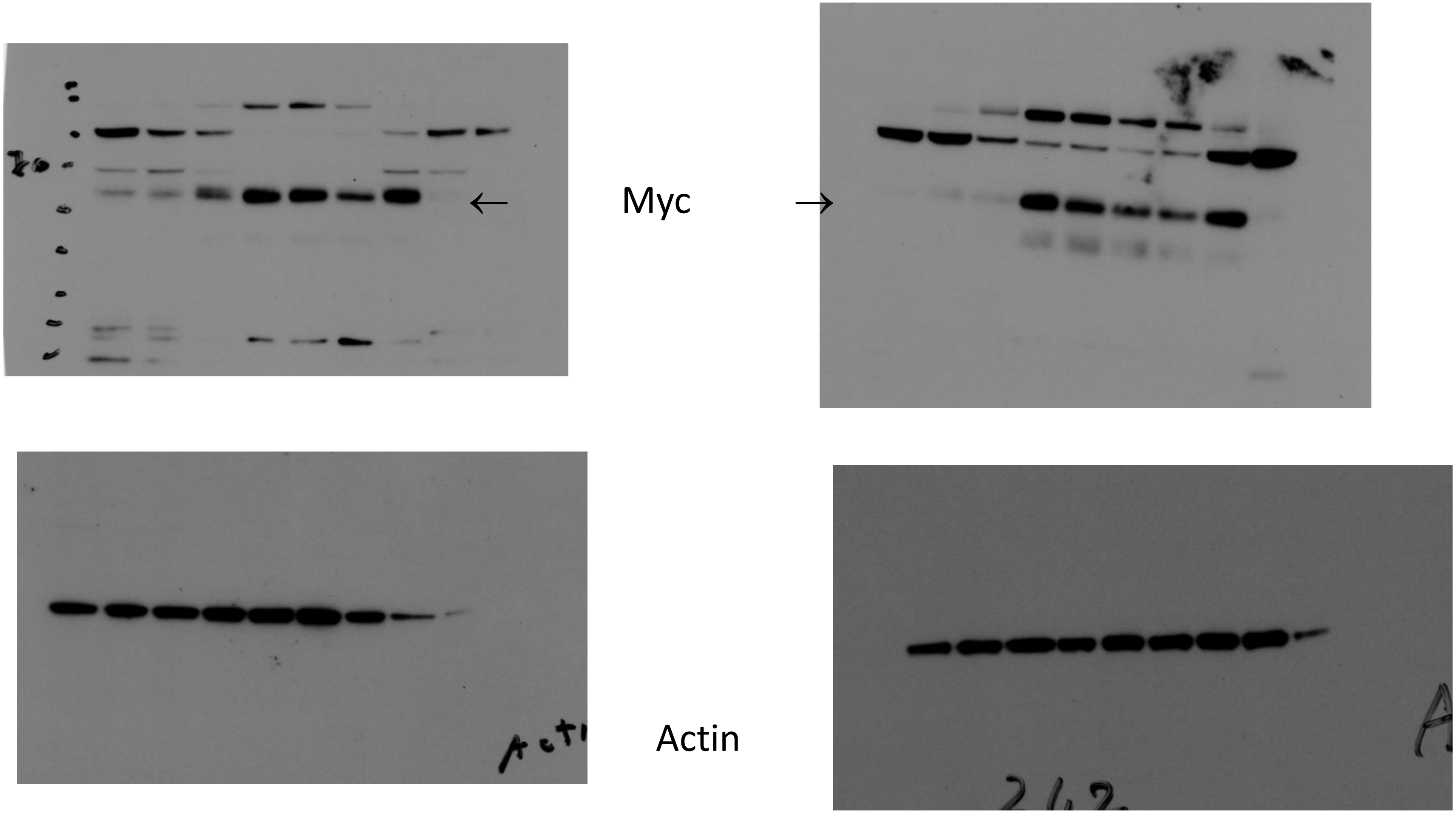

Fig5E

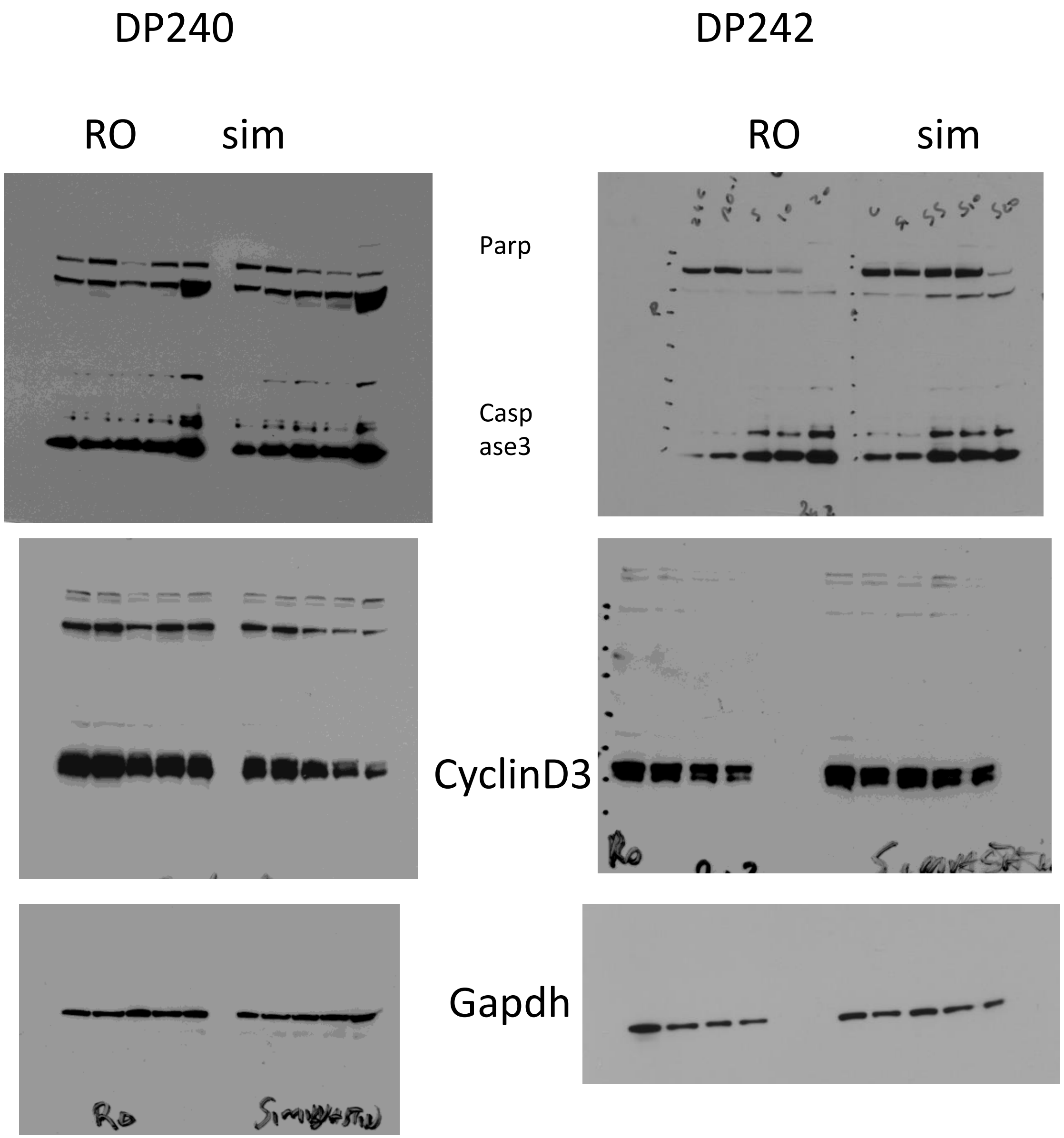

Fig.5G DP240

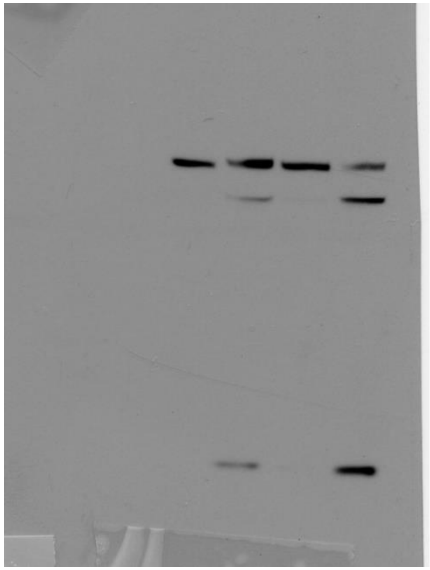

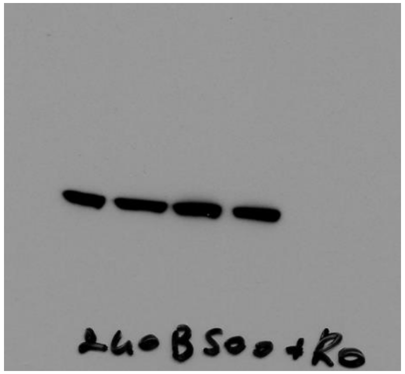

Parp + Caspase3

Gapdh

Fig.5G DP242

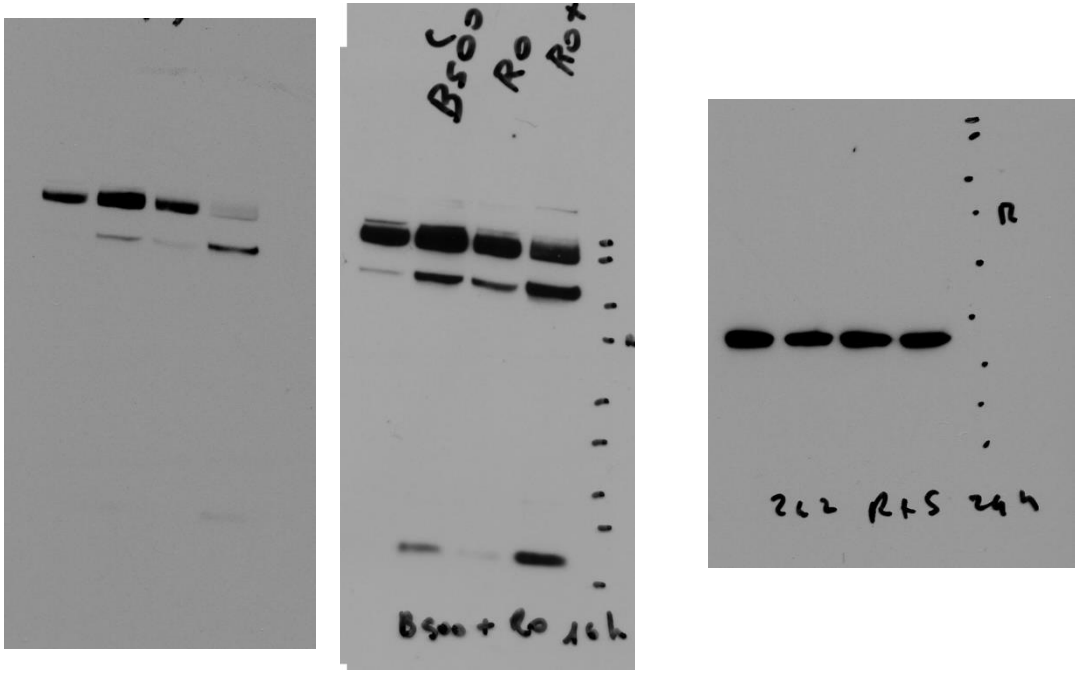

Parp Caspase3 Gapdh

Fig6A

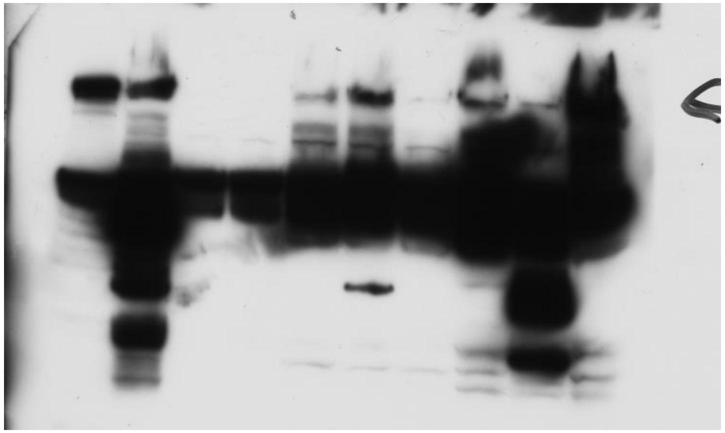

←NOTCH1→

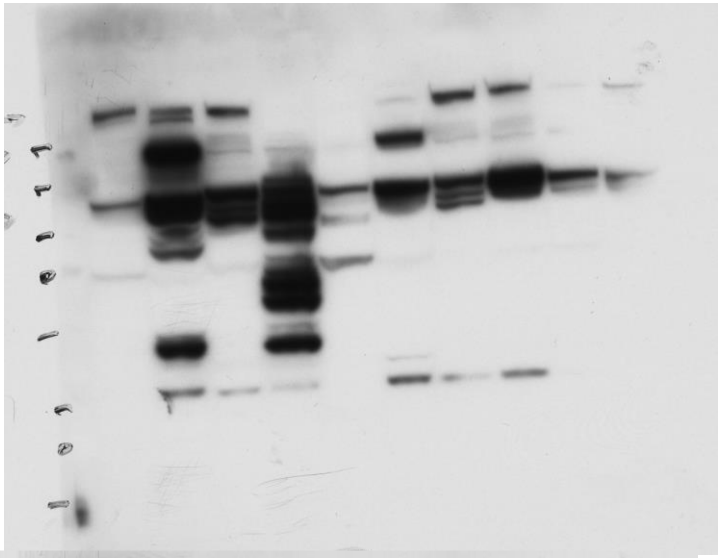

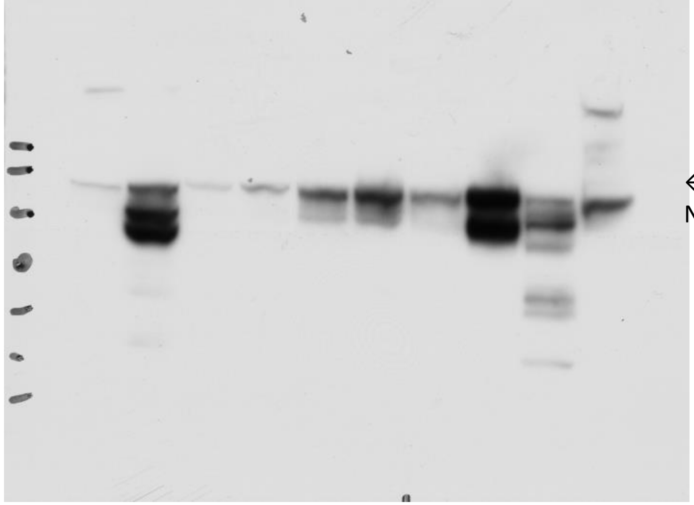

←Cleaved
NOTCH1 →

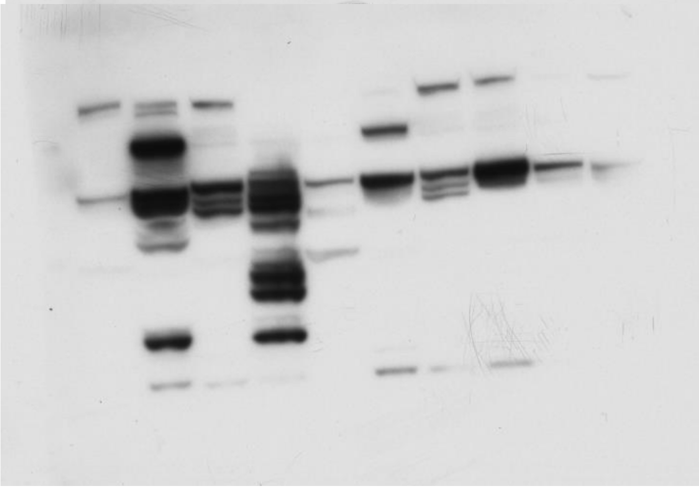

Fig6A

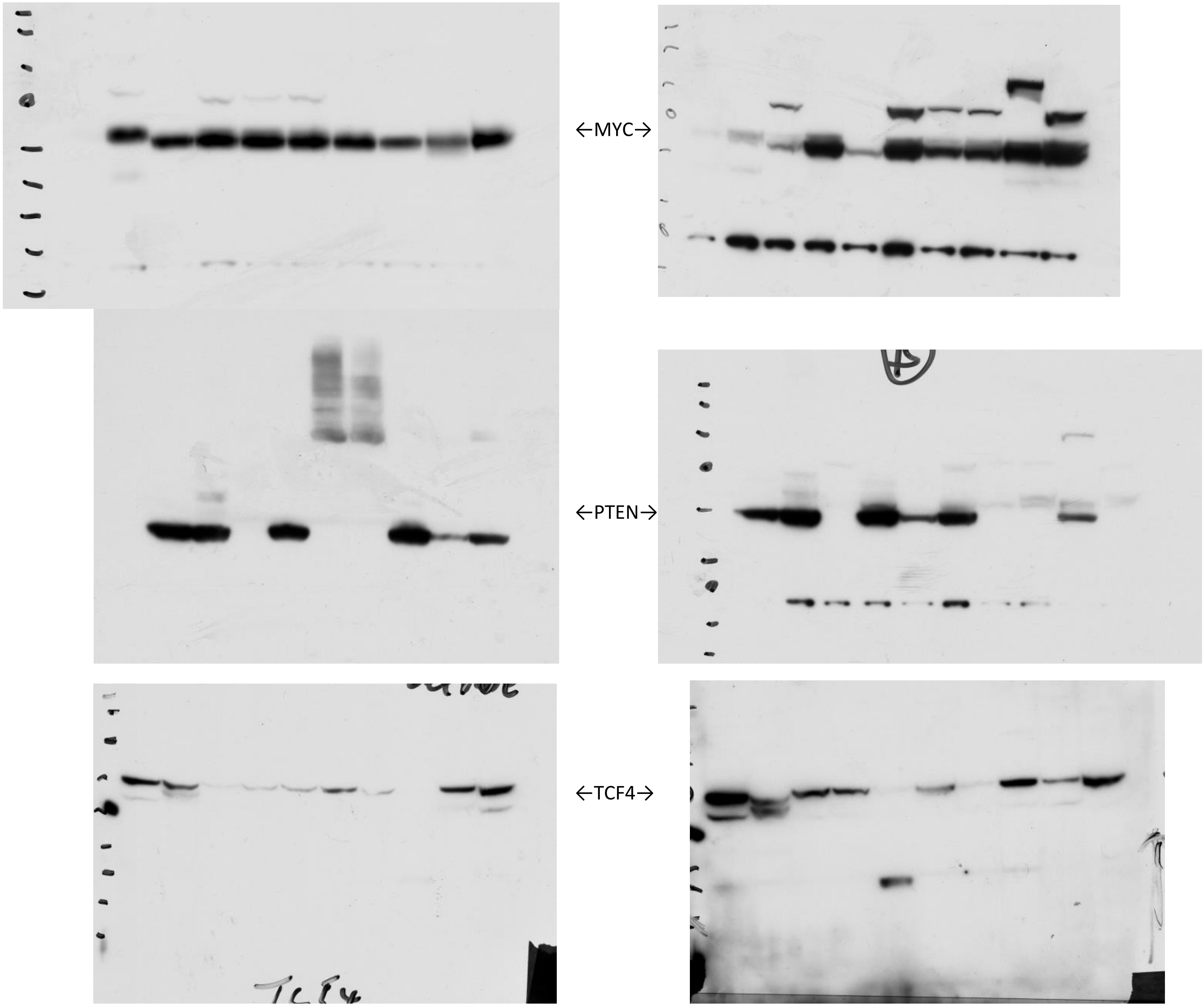

Fig6A

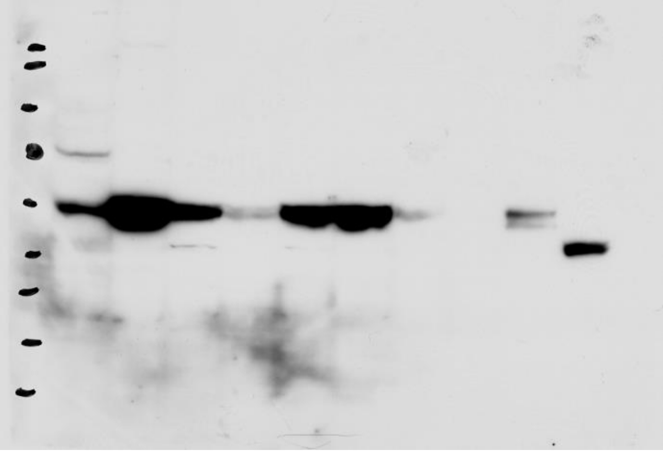

←LEF1→

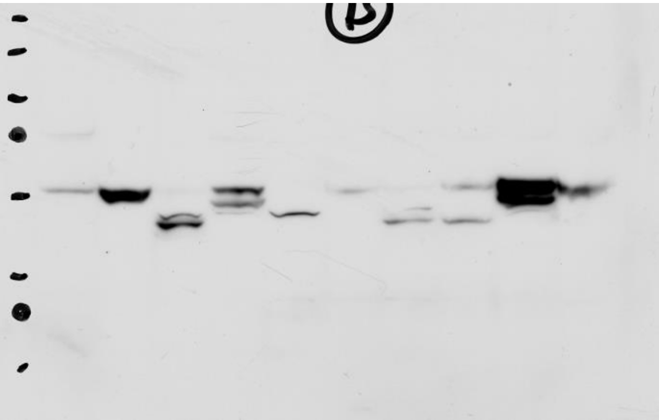

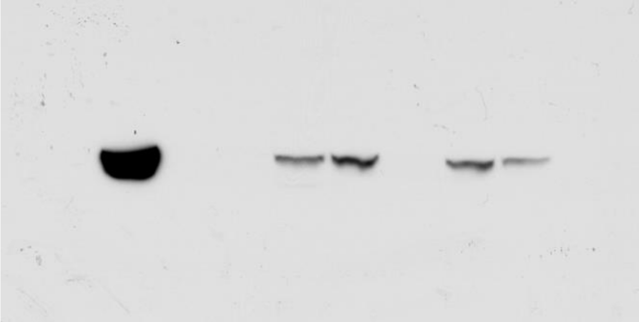

Beta-CATENIN

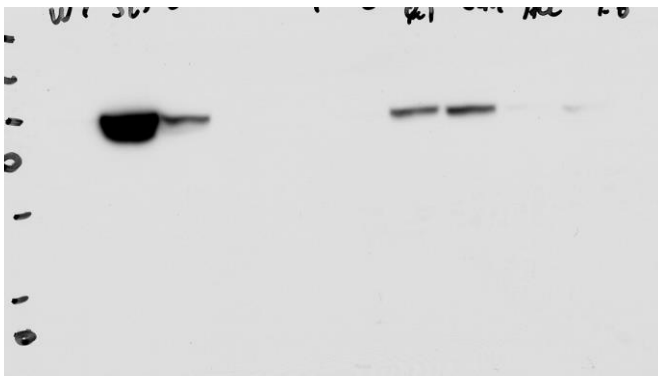

Fig6A

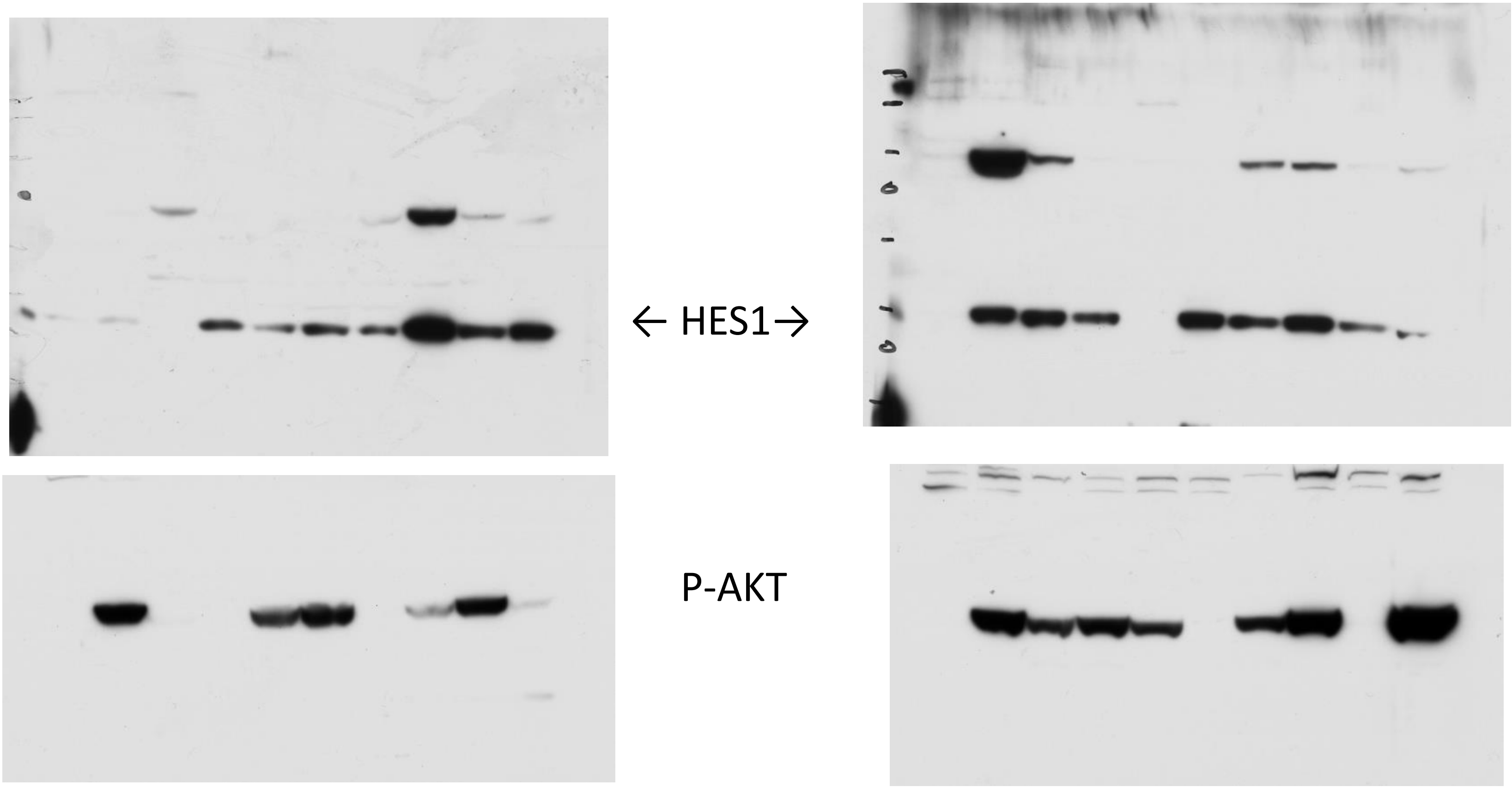

Fig6A

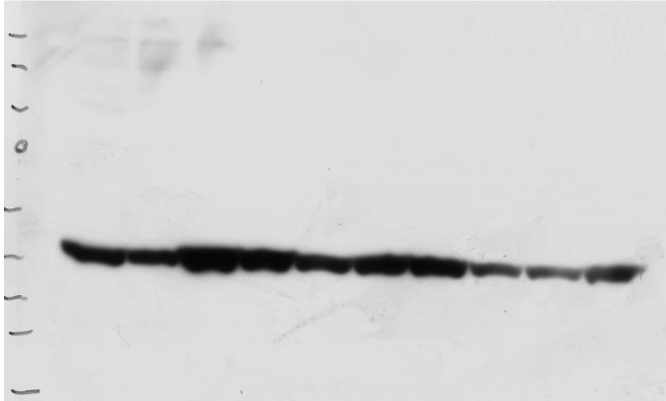

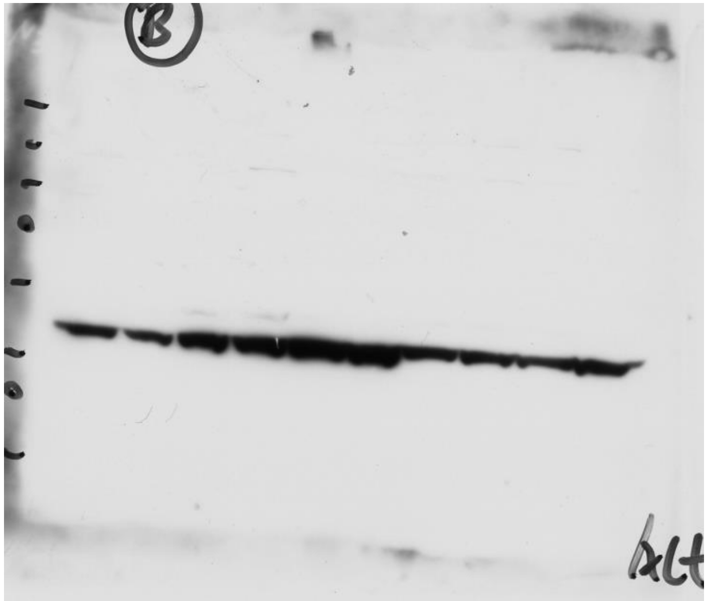

ACTIN

Fig6B

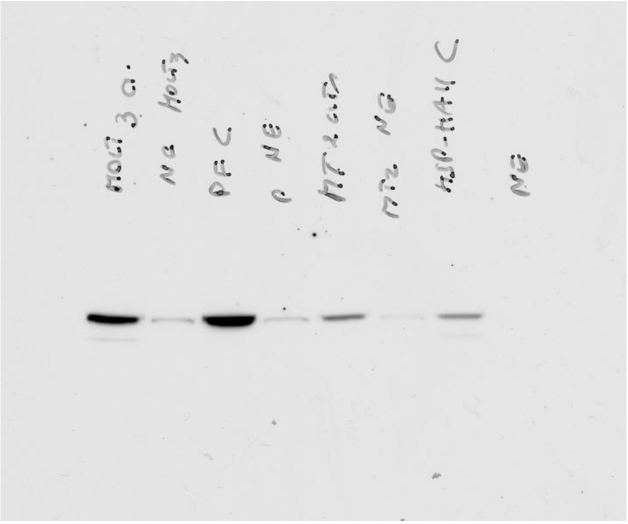

β-Catenin

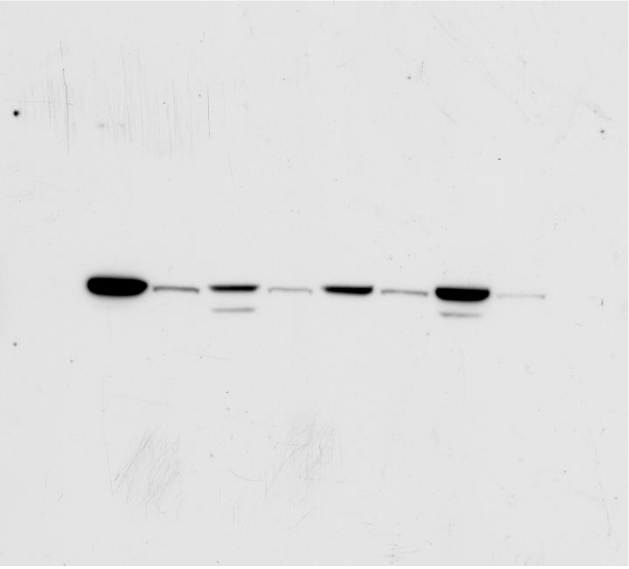

β-Catenin

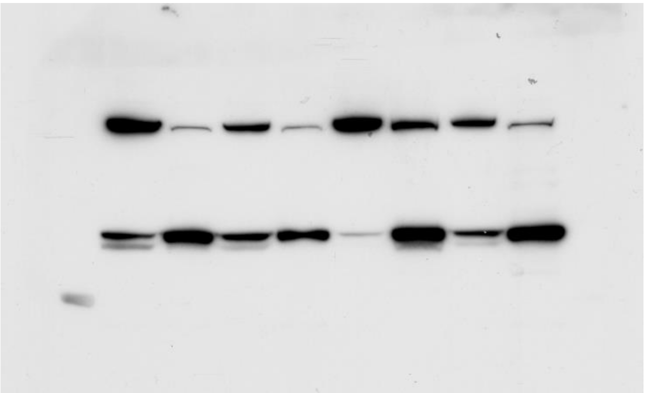

Hsp90&LaminB

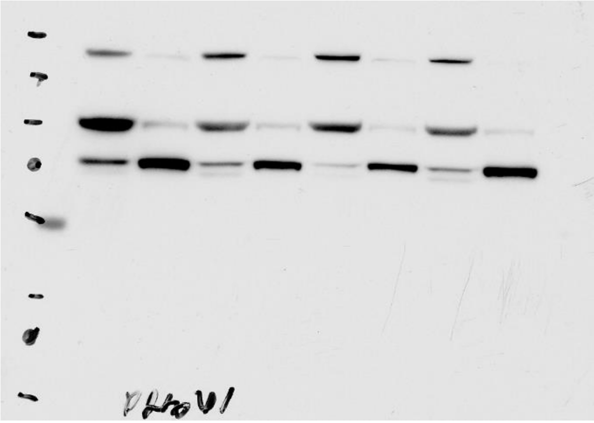

Hsp90&LaminB

Fig6E

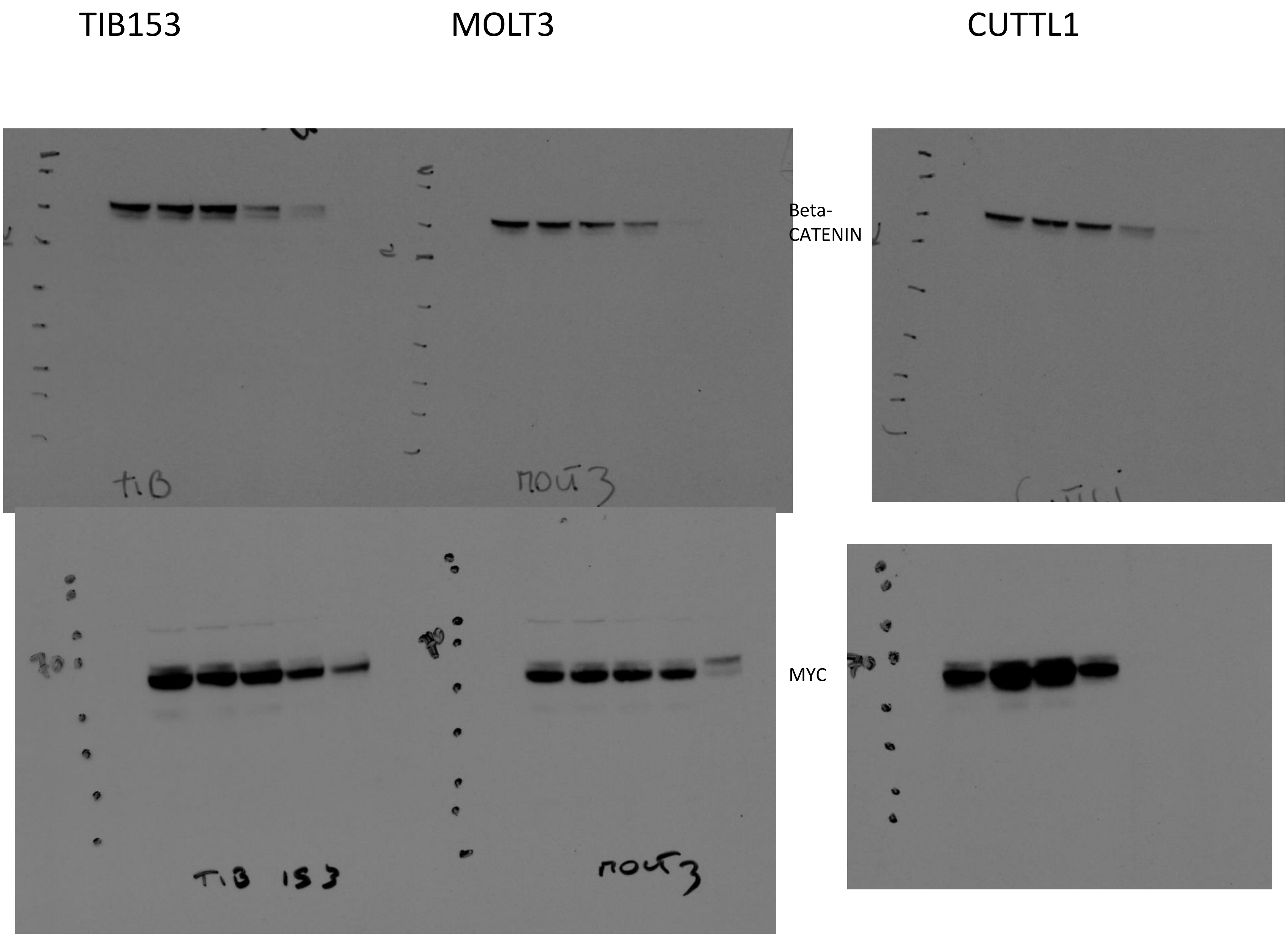

Fig6E

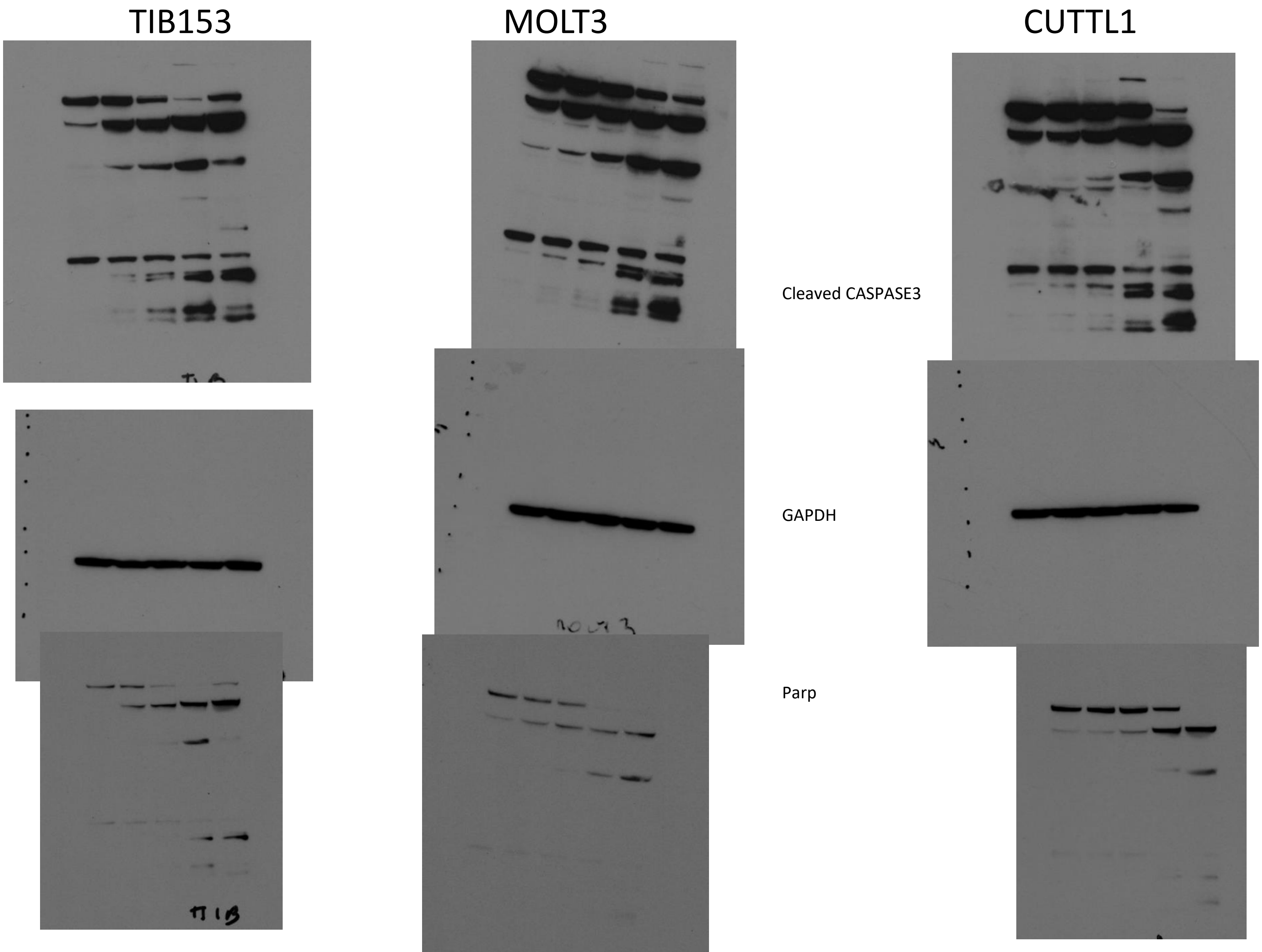

Fig.7C-MOLT3

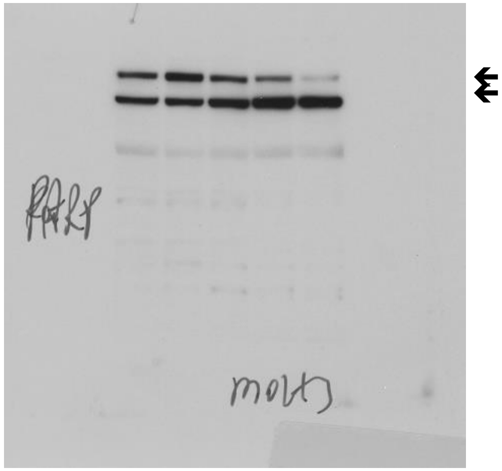

PARP

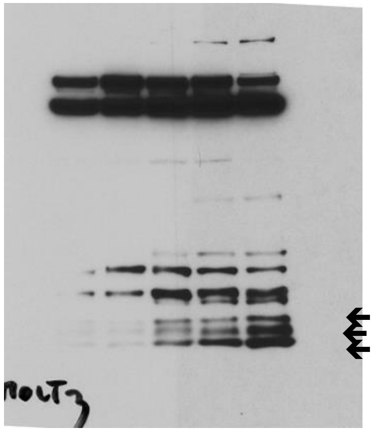

Caspase3

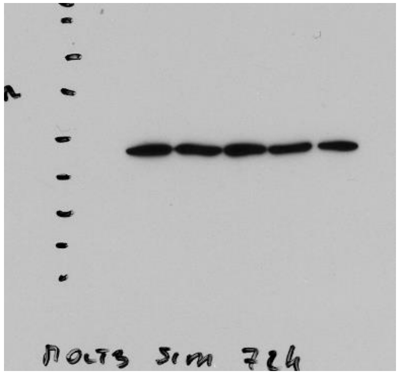

GAPDH

Fig.7C-CUTTL1

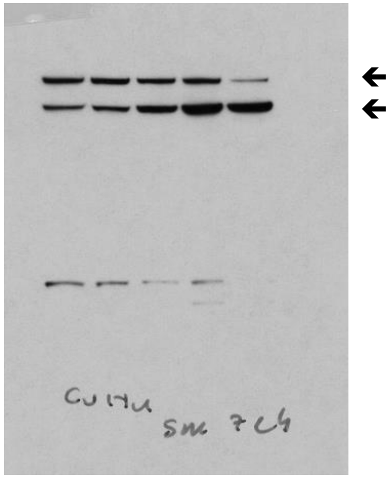

PARP

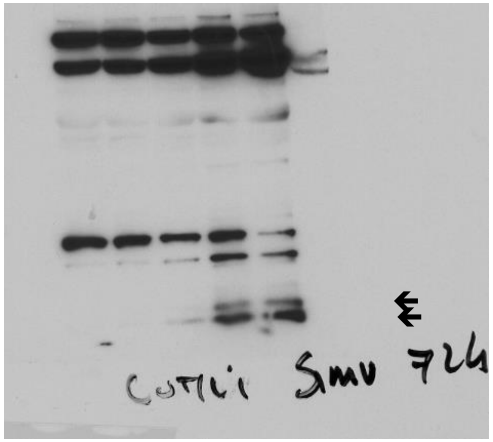

Caspase3

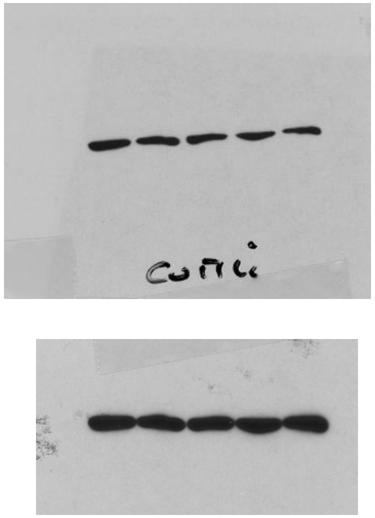

GAPDH

Fig.7C-TIB-153

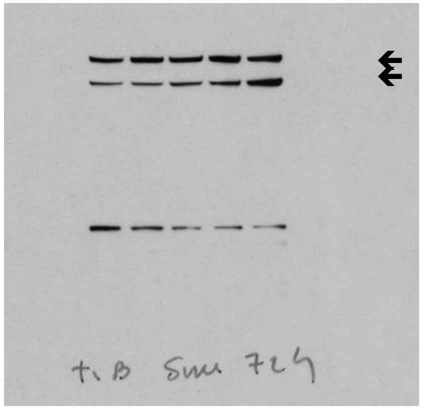

PARP

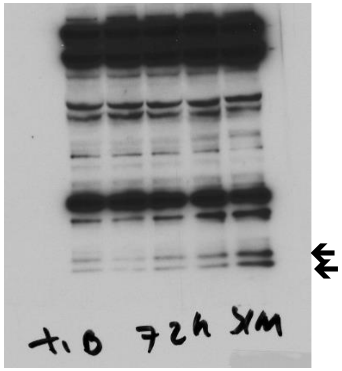

Caspase3

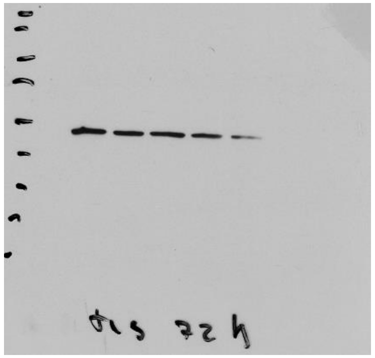

GAPDH

Fig.7E-MOLT3

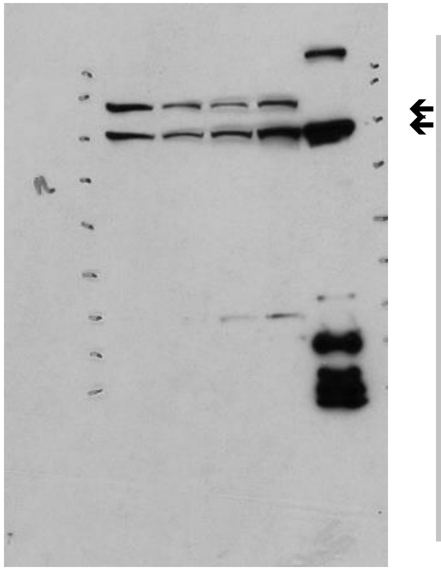

PARP+Caspase3

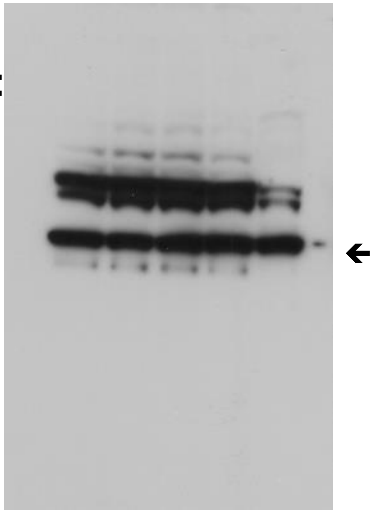

GAPDH

Fig.7E-TIB-153

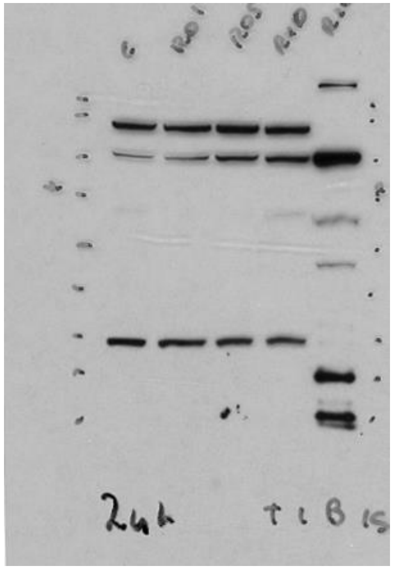

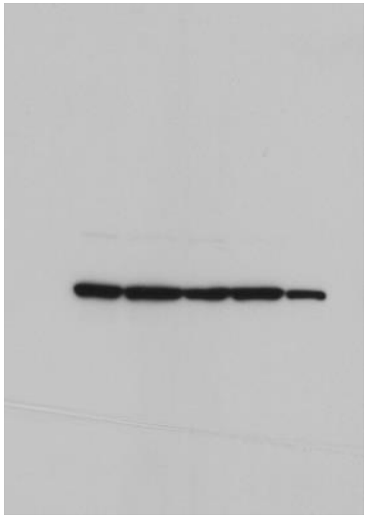

PARP+Caspase3

GAPDH

Fig.7E-CUTTL1

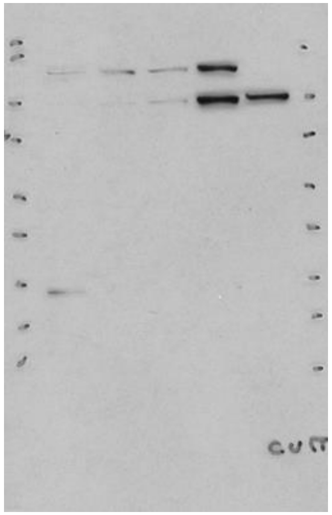

PARP+Caspase3

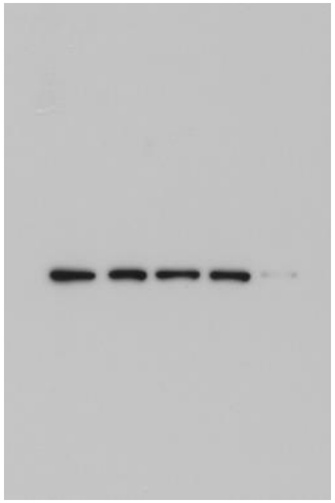

GAPDH

Fig.7G-CUTTL1

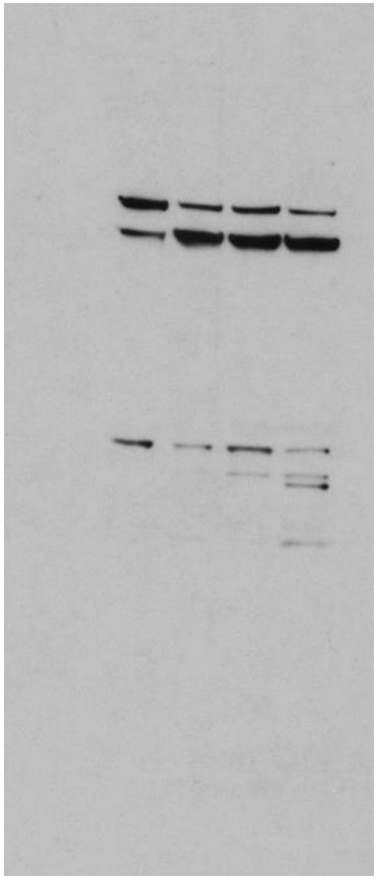

PARP

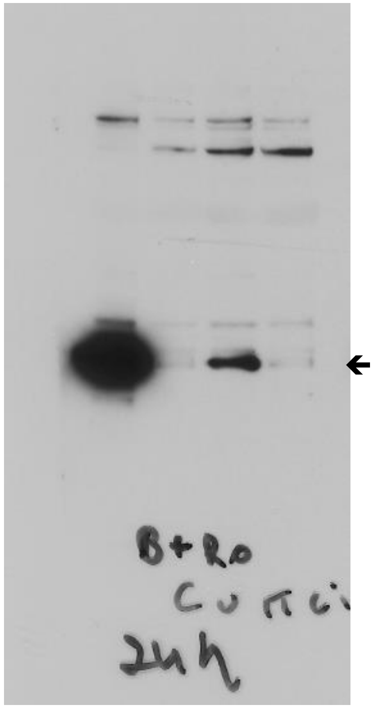

p-S6

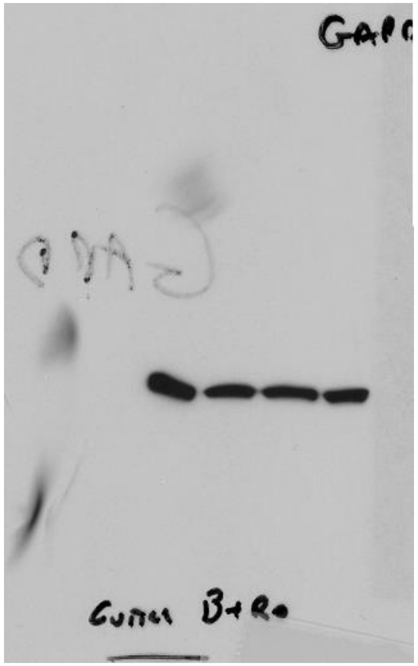

GAPDH

Fig.7G-TIB-153

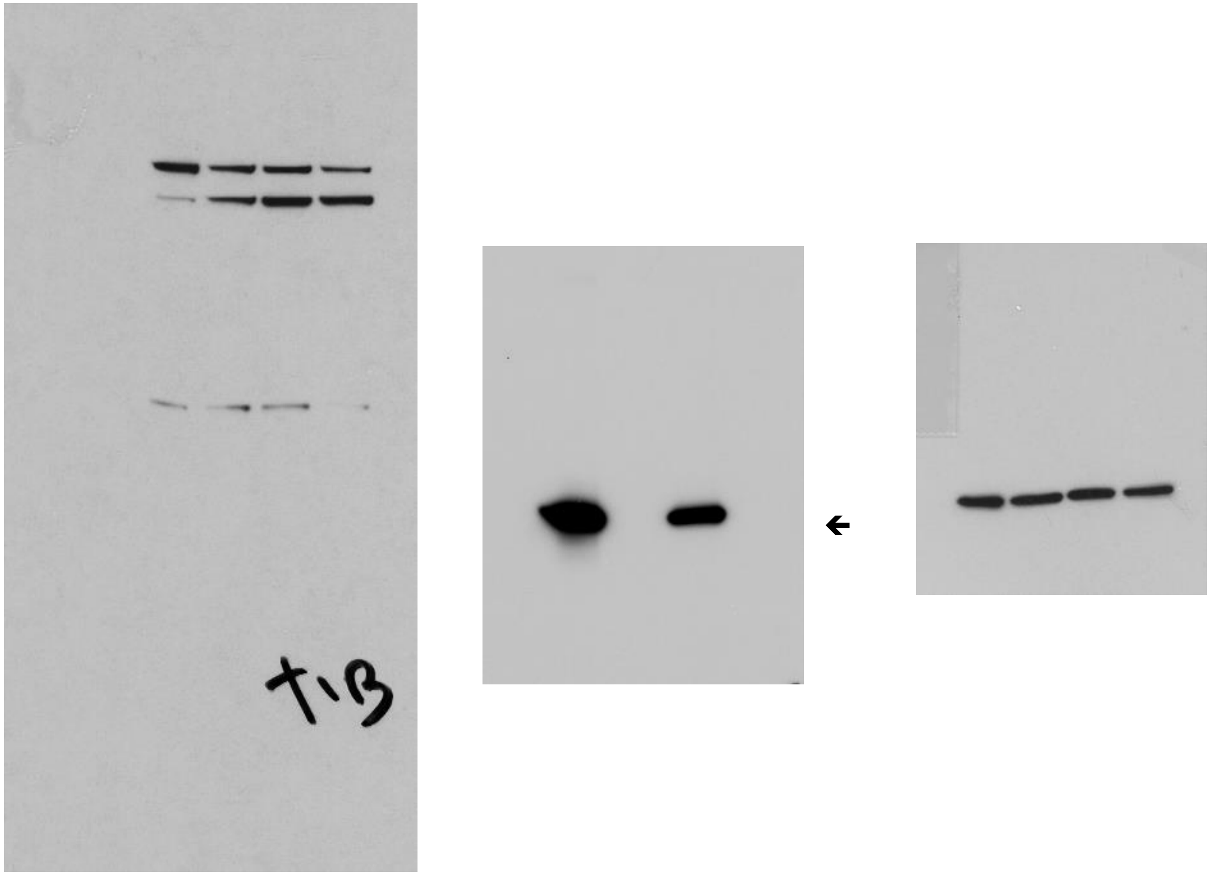

PARP p-S6 GAPDH

Fig.7G-MOLT3

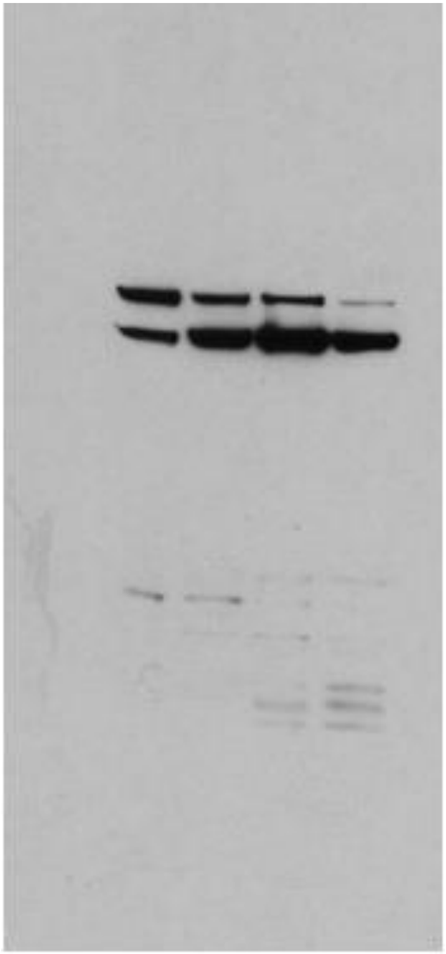

PARP

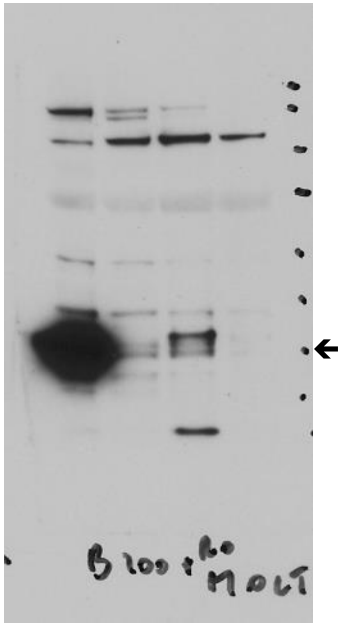

p-S6

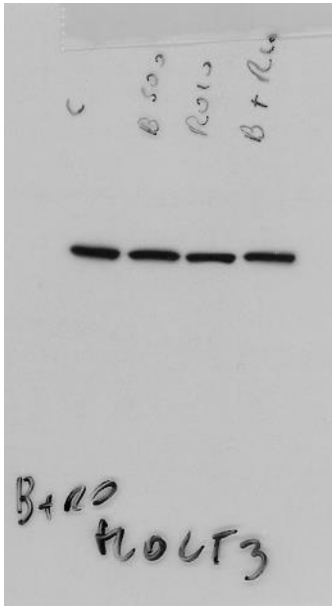

GAPDH
